# BiGGEsTS: integrated environment for biclustering analysis of time series gene expression data

**DOI:** 10.1186/1756-0500-2-124

**Published:** 2009-07-07

**Authors:** Joana P Gonçalves, Sara C Madeira, Arlindo L Oliveira

**Affiliations:** 1 Knowledge Discovery and Bioinformatics (KDBIO) group, INESC-ID, Rua Alves Redol, Apartado 13069, 1000-029 Lisboa, Portugal; 2 Instituto Superior Técnico, Technical University of Lisbon, Av. Rovisco Pais, 1049-001 Lisboa, Portugal; 3 University of Beira Interior, Rua Marquês d'Ávila e Bolama, 6201-001 Covilhã, Portugal

## Abstract

**Background:**

The ability to monitor changes in expression patterns over time, and to observe the emergence of coherent temporal responses using expression time series, is critical to advance our understanding of complex biological processes. Biclustering has been recognized as an effective method for discovering local temporal expression patterns and unraveling potential regulatory mechanisms. The general biclustering problem is NP-hard. In the case of time series this problem is tractable, and efficient algorithms can be used. However, there is still a need for specialized applications able to take advantage of the temporal properties inherent to expression time series, both from a computational and a biological perspective.

**Findings:**

BiGGEsTS makes available state-of-the-art biclustering algorithms for analyzing expression time series. Gene Ontology (GO) annotations are used to assess the biological relevance of the biclusters. Methods for preprocessing expression time series and post-processing results are also included. The analysis is additionally supported by a visualization module capable of displaying informative representations of the data, including heatmaps, dendrograms, expression charts and graphs of enriched GO terms.

**Conclusion:**

BiGGEsTS is a free open source graphical software tool for revealing local coexpression of genes in specific intervals of time, while integrating meaningful information on gene annotations. It is freely available at: . We present a case study on the discovery of transcriptional regulatory modules in the response of *Saccharomyces cerevisiae *to heat stress.

## Background

Extracting relevant biological information from expression data provides important insights into the relations between genes participating in biological processes. This information can be used to identify co-regulated genes corresponding to transcriptional regulatory modules, thus contributing to the challenging goal of regulatory network inference.

Processing expression data is time and resource consuming. In this context, the development of novel computational algorithms and tools for expression data analysis is primarily focused on efficiency and robustness. Clustering techniques have been extensively applied to both dimensions of expression matrices separately, focusing on either gene or sample expression patterns. However, many patterns are common to a subset of genes only in a specific subset of experimental conditions. In fact, our general understanding of cellular processes leads us to expect subsets of genes to be coexpressed only in certain experimental conditions, but to behave almost independently in other. These local expression patterns can only be discovered using biclustering techniques [1,2], which may be the key for uncovering many regulatory mechanisms that are not apparent otherwise [3]. Although the majority of the biclustering formulations are NP-hard [1], the biclustering problem becomes tractable when expression levels are measured over time, restricting the analysis to biclusters with consecutive time points [4-7].

BiGGEsTS (BiclusterinG Gene Expression Time Series) is a free and open source graphical application using state-of-the-art biclustering algorithms specifically developed for analyzing gene expression time series. The current version integrates the methods proposed by Zhang et al. [5] and Madeira and Oliveira [6,7]. An alternative approach from Ji and Tan [5] was not included due to complexity issues [6]. The integration of additional algorithms pursuing similar goals is straightforward. In addition, BiGGEsTS offers well-known preprocessing techniques to filter genes, treat missing values and to smooth, normalize, and discretize expression data. A visualization module supports the analysis of both data and results. Graphical representations include colored matrices (heatmaps), expression and pattern charts, and dendrograms. Biclusters can be studied using Gene Ontology (GO) annotations. BiGGEsTS is also able to generate ontology graphs representing enriched GO terms, and filter and/or sort biclusters according to several numerical and statistical criteria.

### Related tools

Several applications are available for the analysis of gene expression data using clustering [8-15], biclustering [16,17] or both [18,19] approaches. For clustering we highlight Genesis [8], which implements hierarchical clustering, *k*-means, self-organizing maps (SOMs), principal component analysis and support vector machines, together with filtering and normalization methods.

Expander [18] and BicAT [19] offer both clustering (hierarchical and *k*-means) and biclustering techniques. Expander also performs clustering using SOMs and CLICK [18]. Regarding biclustering, Expander uses SAMBA [20], and BicAT integrates the Cheng and Church approach [2], the Iterative Signature algorithm (ISA) [21], the Order-preserving Submatrix method (OPSM) [3], xMotif [22] and BiMax [19]. Both tools include preprocessing methods such as filtering, normalization, log transformation and discretization. Expander further evaluates the biological relevance of clusters/biclusters by computing the functional enrichment of GO terms and retrieves information on promoter signals.

Few applications actually address the problem of analyzing time series and they typically apply clustering [11-14]. CAGED [11] and GQL [12] model expression profiles using Markov chains. CAGED applies agglomerative clustering to group genes with similar expression profiles, while GQL combines the individual Markov models into a mixture model. STEM [13] uses a greedy clustering algorithm.

TimeClust [14] offers hierarchical clustering and SOMs, together with Bayesian and temporal abstraction approaches. To our knowledge, only PAGE [17] provides a biclustering algorithm specifically designed for expression time series, which is a modified version of the approach of Ji and Tan [4]. Most of these applications miss essential preprocessing steps useful to clean and prepare data for analysis.

## Methods

**Figure 1:**
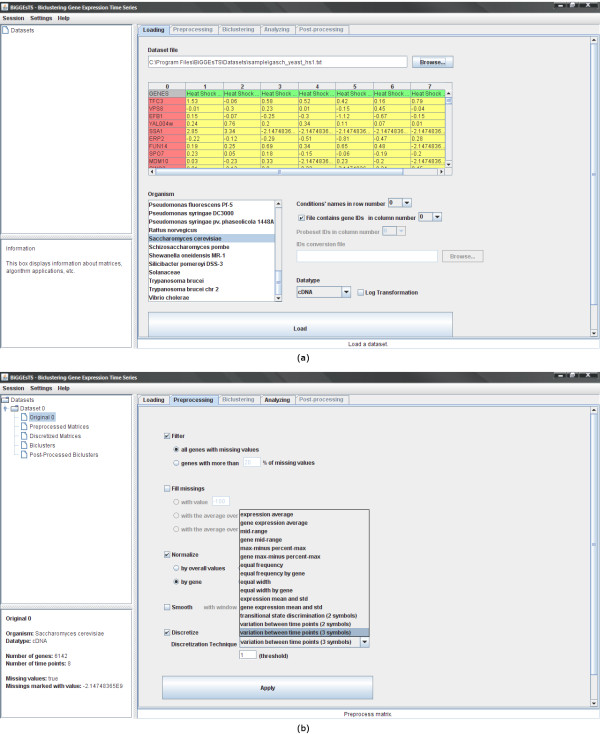
**Input and preprocessing modules**. This figure shows: (a) The main window of BiGGEsTS and its input module for loading time series gene expression data. The graphical user interface (GUI) includes a set of tabs, for functionality selection, and three panels: a top-left panel displaying the dataset tree, where expression matrices and biclusters are organized; a bottom-left panel displaying a box with information about the selected node in the dataset tree; and a right panel, whose content depends on both the selected node and functionality tab. The navigation on the dataset tree, as well as on the tabs, is intuitive and straightforward. A session can be saved anytime to keep record of data and results. Saved sessions can be loaded later enabling researchers to recover previous stages of their analysis. The input of expression time series is performed using a standard text file. The file contains the elements of the gene expression matrix delimited by a specific character (usually tab), together with additional information about the data, including the organism, and the row and column specifying the names of the time points and the genes, respectively. When the names of the genes used in the biological experiments and their corresponding symbol approved by the Human Genome Organization (HUGO) Gene Nomenclature Committee (HGNC) differ, the researcher may want to provide an additional file. This input file is optional, since it is only required for retrieving the gene annotations and assessing the biological relevance of the biclusters. (b) The preprocessing module for filtering genes, filling missing values, normalizing, smoothing and/or discretizing gene expression data. Available preprocessing techniques are described in the Quickstart Guide [see Additional file 2].

This section describes the functionalities of BiGGEsTS from a biology/medical researcher's perspective, providing further insight into the underlying methods [see Additional file 1] [see Additional file 2] [see Additional file 3]. The graphical user interface (GUI) includes a set of tabs and three panels (Figure 1). Main functionalities are directly selected in the tabs and guide the researcher through a complete process of analysis: 1) input and preprocessing of expression data, 2) identification of coexpressed genes in specific subsets of time points using biclustering (biclusters), 3) post-processing of biclusters in order to rank the results, 4) usage of exploratory analysis tools (visualization options, GO annotations and functional enrichment of GO terms).

### Input and preprocessing of time series gene expression data

The input of expression time series is straightforward (Figure 1(a)) and is usually followed by a set of preprocessing steps (Figure 1(b)). These handle occasional and systematic errors, reduce noise, and prepare data to be analyzed.

Occasional errors may occur when measuring the abundance of mRNA in cells, leading to missing values, not always supported by biclustering algorithms. This can be addressed by filtering all genes with missing values, thus eliminating all rows with at least one missing value, and may be regarded as a good strategy to reduce noise. However, when analyzing a small number of genes, removing some of them can lead to a significant reduction in the dimension of the dataset, potentially compromising further analysis. The tradeoff between the elimination of missing values and the dimension of the dataset is usually mitigated by establishing an upper bound for the percentage of missing values allowed per gene. Genes with percentages higher than a user-defined threshold are filtered. The remaining missing values must be filled.

Systematic errors, on the other hand, affect every measurement action and are associated with the differences between the experimental settings of each trial. Sources of this kind of errors include the different incorporation efficiency of dyes, and the different scanning and processing parameters of distinct experiments. Normalization is a widely used technique, which attempts to compensate for these systematical differences between time points and highlight the similarities and differences in the expression profiles. Additionally, a smoothing algorithm acts as a low-pass filter, attenuating the effect of outliers. Depending on the biclustering algorithm, it may be necessary to discretize data, reducing the range of expression values to an adequate set of discrete values.

### Biclustering

**Figure 2:**
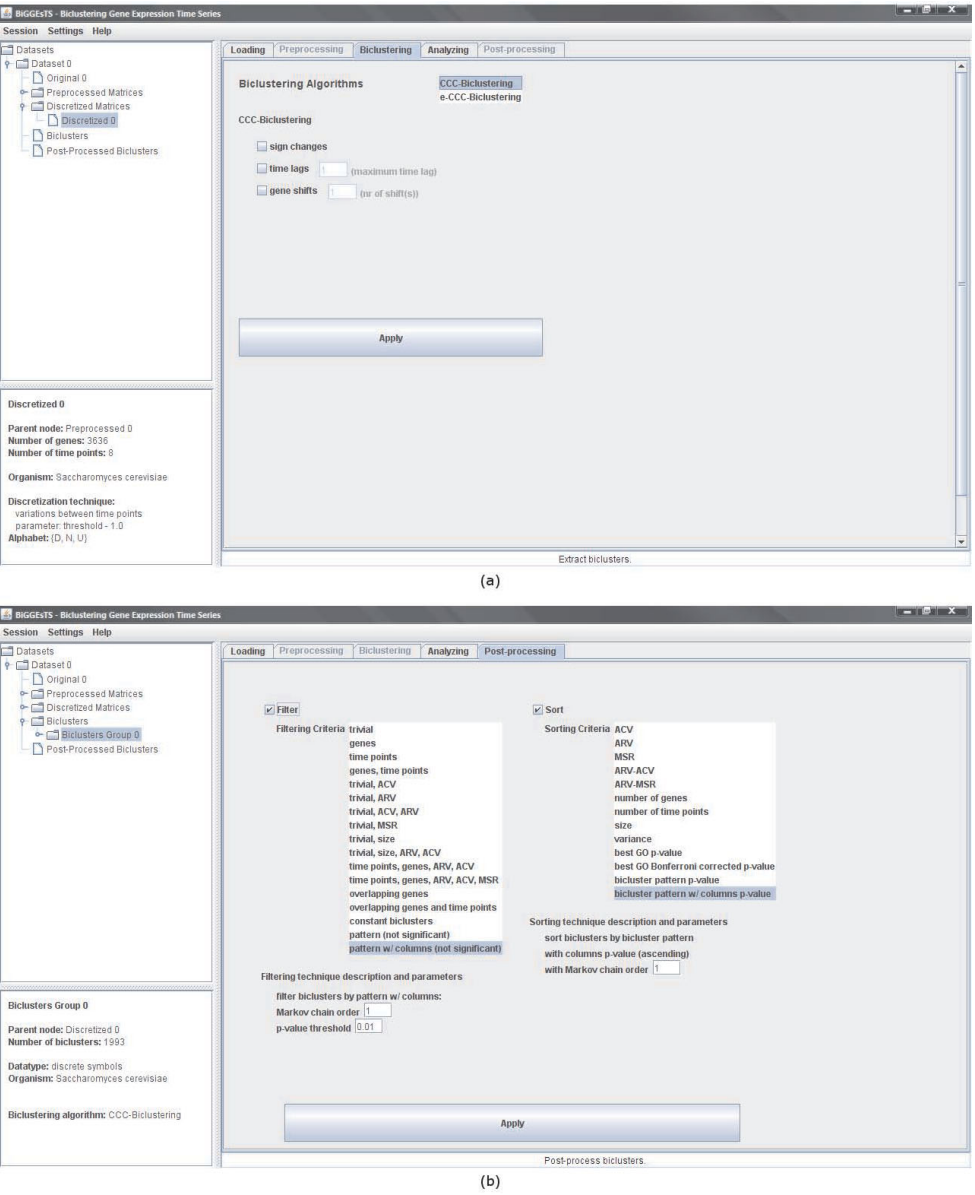
**Biclustering and post-processing modules**. This figure shows: (a) biclustering and (b) post-processing modules. The biclustering module is used to select the biclustering algorithm to be applied to the expression matrix. Additional extensions enabling shifted, anti-correlated and time-lagged patterns are available in CCC-Biclustering and *e*-CCC-Biclustering. Different types of errors are supported in *e*-CCC-Biclustering. The post-processing module enables the researcher to select and apply filtering and sorting techniques to groups of biclusters. Biclusters can be filtered by setting a threshold for the number of genes and/or conditions, size, average column variance, average row variance, mean-squared residue score, and overlapping percentage of genes and/or conditions. It is also possible to remove biclusters with constant or statistically non significant patterns. Biclusters may additionally be sorted using their best functional enrichment *p*-value, statistical significance of expression pattern, average column or row variance, mean-squared residue score and a number of other measures available for selection. Details on biclustering and post-processing techniques are described in the Quickstart Guide [see Additional file 2].

Three biclustering algorithms are available: CCC-Biclustering [6], *e*-CCC-Biclustering [7] and CC-TSB [5] (Figure 2(a)). The first two process discretized matrices, while the third uses real-valued data. CCC-Biclustering uses a generalized suffix tree to identify, in time linear on the size of the expression matrix, all maximal biclusters with contiguous columns that exhibit coherent expression evolutions over time. In a CCC-Bicluster, all genes have exactly the same discretized expression pattern.

*e*-CCC-Biclustering extracts all maximal CCC-Biclusters with approximate expression patterns in time polynomial on the size of the expression matrix. The expression patterns in an *e*-CCC-Bicluster may vary from one gene to another, as long as the number of errors between each pattern and the pattern profile does not exceed a predefined value. Two kinds of errors are supported: general and restricted. General errors identify measurement errors and allow symbols to be substituted by any other symbol in the discretization alphabet. Restricted errors identify discretization errors and only consider as valid the substitutions of symbols by a predefined number of neighbors in the discretization alphabet.

Both CCC-Biclustering and *e*-CCC-Biclustering are provided with three additional extensions that identify biclusters with shifted/scaled, anti-correlated and time-lagged patterns [23]. Sometimes, distinct genes exhibit similar expression evolutions at different expression levels, thus not reflecting a similar pattern after discretization. This problem is addressed by identifying biclusters with shifted patterns. Anti-correlation allows genes with opposite expression patterns, in a set of consecutive time points, to be included in the same bicluster. The time-lagged approach identifies genes that exhibit similar expression patterns starting at different time points, enabling the identification of activation/inhibition delays.

CC-TSB is an adaptation of the biclustering algorithm proposed by Cheng and Church [2]. This heuristic approach uses the mean squared residue (MSR) as merit function and iteratively alternates the addition/removal of genes/time points, forcing the MSR to reduce. The addition/removal of time points is restricted to discover only biclusters with contiguous columns.

### Post-processing

Applying biclustering to expression data often yields a large number of biclusters. Since analyzing all is usually prohibitive, post-processing techniques are performed in order to rank biclusters according to their relevance. Several methods are available to filter and sort biclusters based on numerical and statistical criteria (Figure 2(b)).

### Biological analysis

Biclustering groups genes and conditions based on relations inferred from data, relying strictly on computational methods. Researchers are usually interested in analyzing the results looking for statistically significant biological phenomena. This significance can be assessed using Ontologizer's term-for-term analysis [24], which computes the functional enrichment of the genes in the biclusters by identifying the overrepresented GO terms. In a first step, GO annotations are extracted requiring two distinct files (downloadable from the GO repository if not available) containing the complete ontology and organism-dependent annotations. Term-for-term analysis is then applied to compute a *p*-value for each GO term. Such *p*-value is calculated with respect to the null hypothesis that the inclusion of genes follows a hypergeometric distribution, and measures the statistical significance of each term by computing the ratio of the frequencies of annotated genes in the bicluster and in the complete dataset. The Bonferroni correction for multiple testing is applied. The lower the *p*-value, the more significant the term is. According to standard statistical practice, terms with a corrected *p*-value lower than 0.05 and 0.01 are considered significant and highly significant, respectively.

### Visualization

A visualization module provides different graphical representations of expression time series, enhancing their most important features: tables of values, colors and symbols; dendrograms; expression charts; pattern charts; tables of GO terms and functional enrichment; and graphs of enriched terms.

### Expression matrices and heatmaps

**Figure 3:**
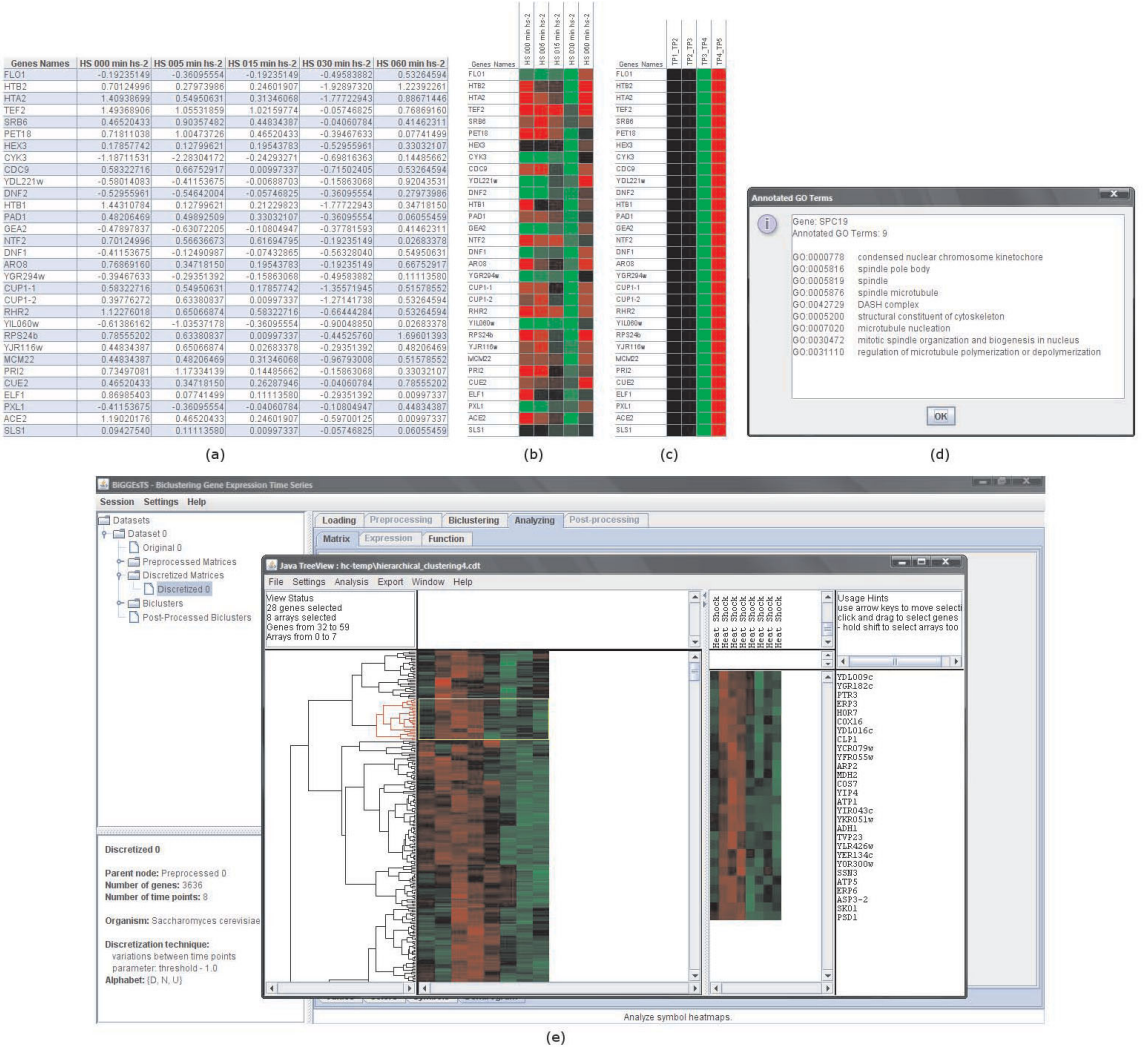
**Expression matrix, heatmaps, GO annotations, and dendrograms**. This figure shows: (a) tables of values, (b) tables of colors, (c) tables of symbols, (d) list of GO terms annotating a gene, and (e) dendrograms. In the tables of values, the names of the experimental conditions appear in the first row and usually correspond to consecutive instants in time. The first column displays the names of the genes. Each remaining cell in the table contains the expression value of a given gene in a specific condition. In the tables of colors, cells with high expression values are, by default, colored red, while the ones with low expression values are given a green color. Cells holding the mean value are colored black. The intensity of the color is set according to the actual expression value of each cell, thus generating a scale of reds and greens for all possible expression values. Cells with no expression value, that is, a missing value, are given a yellow color. Tables of symbols resemble tables of colors and are computed using a discretized version of the expression matrix. The GO terms listed as annotations of a given gene correspond to the most specific GO terms (before applying the true path rule). Dendrograms are visualized using Java TreeView [25] in a separate window. They are displayed together with the expression matrix and enable the researcher to individually select clusters, which are then displayed in a separate panel. The researcher may further change the settings of the dendrogram, search for genes or conditions within the data, compare with other hierarchical clustering results and export both the dendrogram and the gene expression matrix as vector (PS) or raster (PNG, PPM, JPG) image files.

Expression matrices are displayed as tables of values (Figure 3(a)). Tables of colors are commonly known as heatmaps. They resemble tables of values, although each cell is given a different color according to the expression value it contains (Figure 3(b)). Tables of symbols are heatmaps for expression matrices with discrete values. Each cell is given a different color depending on the discretization symbol (Figure 3(c)). Expression tables share additional functionalities. They can be sorted by the values of a given column by clicking on the corresponding table header, provide access to the GO terms that annotate each gene by clicking on its row, and be exported as PNG or JPEG image files by clicking the right button of the mouse over the table and selecting the "Export as image" menu item. GO terms annotating a given gene are displayed in a popup (Figure 3(d)). Since this information is automatically parsed from the GO files (using Ontologizer), no annotations are shown if the GO files are not available. In the latter case, GO files can be downloaded from the GO repository when an Internet connection is available.

### Dendrograms

Dendrograms are branching tree-like diagrams used for representing similarity relationships between the genes/time points in the expression data (Figure 3(e)). The similarity hierarchy is obtained using agglomerative hierarchical clustering to group genes and/or time points. At each step, the cluster pairwise similarity is used to decide which clusters to merge. For single element clusters, this similarity is computed using a distance measure (Euclidean or cityblock) or a correlation coefficient (uncentered, Pearson's, absolute uncentered, absolute Pearson's, Spearman's or Kendall's correlation). Otherwise, a single-linkage, complete-linkage, average-linkage or centroid-linkage approach is used. Java TreeView [25] is used to interpret hierarchical clustering results and display the dendrograms.

### Expression and pattern charts

**Figure 4:**
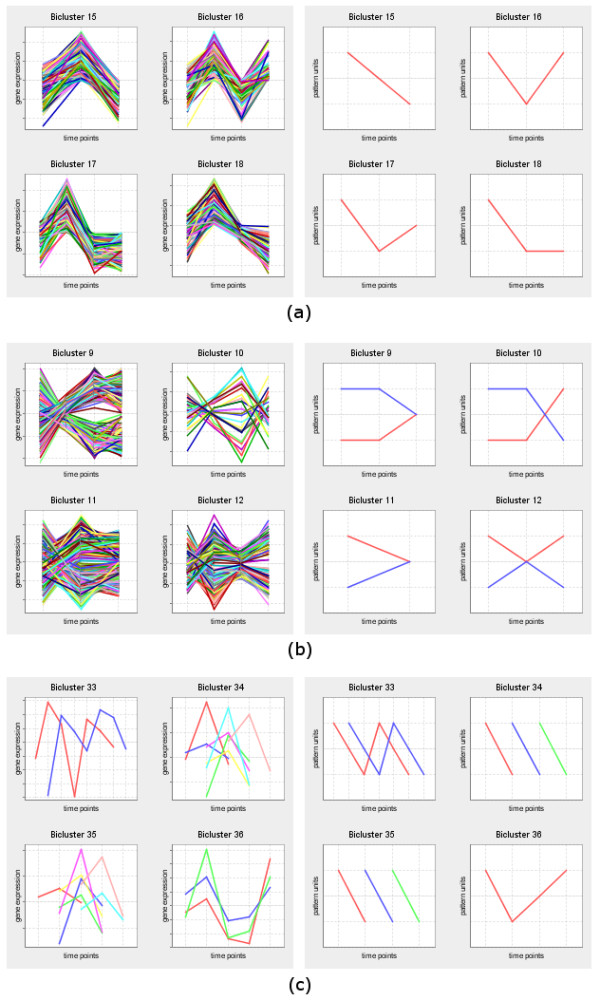
**Expression and pattern charts**. This figure shows examples of expression and pattern charts of (a) CCC-Biclusters, (b) CCC-Biclusters with anti-correlated patterns, and (c) CCC-Biclusters with time-lagged patterns. In expression charts, expression values can be normalized on the fly by checking the "Normalize to mean 0 and std 1" checkbox.

Expression and pattern charts show the evolution of the expression level of the genes in the biclusters over time, using the corresponding submatrices. Expression charts are obtained using real-valued matrices, while pattern charts are generated from discretized matrices (Figure 4).

Expression charts can be displayed using either the subset of time points in the bicluster, or all the time points in the dataset. The latter are particularly suitable for highlighting the coherent behavior of the genes in the bicluster time points as opposed to the uncorrelated behavior in the remaining time points. Both expression and pattern charts provide a context menu with extra functionalities, including displaying and modifying chart properties, saving chart to an image file, printing, and zooming in or out.

### Go terms and functional enrichment

**Figure 5:**
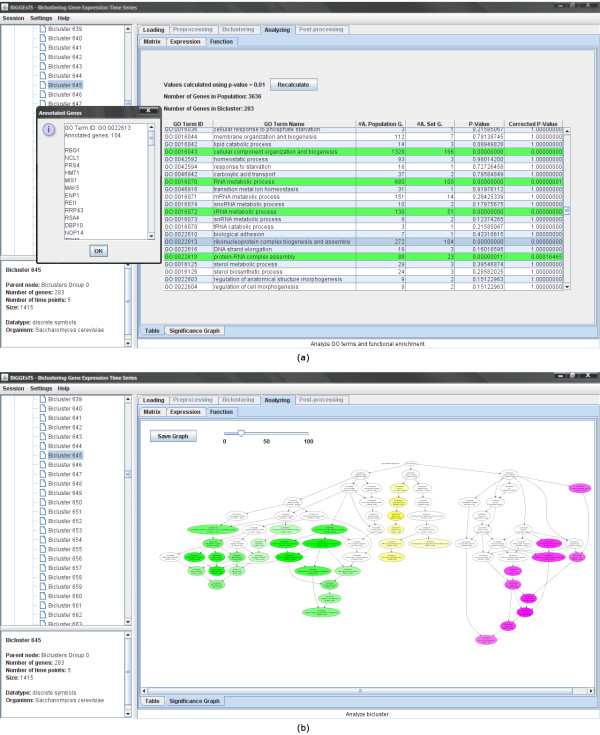
**Term-for-term analysis and graph of enriched GO terms**. This figure shows: (a) A summary of the results of the term-for-term analysis applied to a given bicluster. The list of genes annotated with GO term highlighted in blue is displayed at left. The GO terms highlighted in green correspond to highly significant terms. (b) A graph displaying the distribution of the biological terms in the ontology of GO terms. Enriched terms are colored in purple, yellow or green whether they specialize from cellular component, molecular function or biological process, respectively. The intensity of the color depends on the Bonferroni corrected *p*-value of the corresponding term: the lower the *p*-value, the more intense the color of its node. Arrows define specialization relations: each arrow goes from a more general term to its specialization(s).

The GO terms that annotate the genes in the dataset can be displayed in a table, where each row corresponds to a GO term and contains: the GO term ID, the term name, and the total number of genes annotated with it. In the case of a bicluster (Figure 5(a)), each row in the table further includes the number of genes in the bicluster annotated with the term, the *p*-value computed using the term-for-term analysis, and its Bonferroni corrected *p*-value. Enriched terms, given a threshold (default value is 0.01), are highlighted. Additionally, the list of genes annotated with each term is displayed in a popup window by clicking the corresponding row using the left button of the mouse.

### Graphs of enriched terms

Term-for-term results can be used to generate tree structured graphs highlighting the enriched terms in each of the three GO ontologies (Figure 5(b)). Graphs of enriched terms are generated using Ontologizer [24], which outputs the structure of the graph into a text file. Graphviz [26] is used to convert the text file into an SVG file describing the same graphical structure using the XML standard. Finally, the Batik SVG Toolkit [27] is used to interpret the SVG file and display the corresponding image. The graph with the enriched terms can be zoomed in or out and saved as a raster (PNG) or vector (SVG) image file.

## Conclusion

BiGGEsTS is a software for analyzing gene expression time series using biclustering. It was designed to comply with the broad specifications of a software tool, essentially focused on user-friendliness, platform independence, modularity, reusability and efficiency.

Additional material includes a case study describing how to use the software to discover transcriptional regulatory modules in a dataset containing the response of *Saccharomyces cerevisiae *to heat stress [28], reproducing the results published in [6] [see Additional file 4] [see Additional file 5] [see Additional file 6].

## Availability and requirements

• Project name: BiGGEsTS – BiclusterinG Gene Expression Time Series

• Project home page: 

• Operating systems: Platform independent

• Programming language: Java

• Other requirements: Java 1.5 or higher, 1024 MB of RAM, Graphviz (in OSs other than Windows and Mac OS)

• License: GNU GPL version 3 or higher

## Competing interests

The authors declare that they have no competing interests.

## Authors' contributions

SCM implemented the techniques for preprocessing gene expression data, the biclustering algorithms and the post-processing approaches. JPG and SCM designed the software. SCM supervised the implementation of the software. JPG implemented the software and integrated the preprocessing, biclustering, and post-processing methods. Additionally, JPG produced the documentation, created the website, and wrote the first draft of the manuscript. All authors worked together in the final version of the manuscript. All authors read and approved the final manuscript.

## Supplementary Material

Additional file 1 **Multi-platform distribution of BiGGEsTS**. A multi-platform distribution of BiGGEsTS. The archive biggests.zip contains a directory with several files, including installation files, the application, sample datasets and sessions, the Quickstart Guide to the software ("BiGGEsTS Quickstart.pdf") and a simple text file with installation instructions ("readme.txt"). In Windows (or Mac OS X) run the "install.bat" (or "install.sh" in Mac OS X) file, for installing the Graphviz dot application and the GO files, and then the "biggests.bat" ("biggests.sh") file, for running the software. For Linux and other operating systems, please install the Graphviz dot application first and edit the "install.sh" file to append the path of the dot executable file (typically /usr/bin/dot) to the last line. Then run the "install.sh" script followed by "biggests.sh". Detailed instructions on how to install and use BiGGEsTS are also available in the Quickstart Guide [see Additional file 2]. The latest version of the software is available at the official website. Click here for file

Additional file 2 **BiGGEsTS Quickstart Guide**. This document introduces users to BiGGEsTS, providing instructions on how to install and use this software, to analyze time series gene expression data using biclustering. Click here for file

Additional file 3 **Source code of the BiGGEsTS software**. The source code of BiGGEsTS. The archive contains two directories, named "biggests" and "smadeira", inside a main directory, named "src". Each of the directories contained in "biggests" and "smadeira" contains the source files of the classes included in the packages identified by the same names. The Javadoc documentation of the source code is available at the official website. Click here for file

Additional file 4 **Sample expression dataset (from Gasch et al**. [28]**)**. This file contains a sample time series gene expression matrix corresponding to a short subset of a real dataset from Gasch et al. [28], concerning the yeast response to heat shock. The original dataset analyzes 6142 genes from *Saccharomyces cerevisiae *in 8 time points (5', 10', 15', 20', 30', 40', 60', 80'). The gasch_ yeast_hs1_short.txt file is also included in the multi-platform distribution. To load this dataset into BiGGEsTS, run the software and use the "Browse..." button on the panel on the right to browse the file in the file system. Once it is found, press the "Open" button followed by the "Load" button (a detailed description of the parameters is available in the Quickstart Guide [see Additional file 2]). Click here for file

Additional file 5 **Archive of a sample BiGGEsTS session**. This file contains a BiGGEsTS session with matrices and biclusters obtained by manipulating the time series gene expression data also provided as additional material [see Additional file 4], using BiGGEsTS. The session was exported into this file also using BiGGEsTS. To load the session into BiGGEsTS and explore its contents, run the software, select the "Session" menu, and then the "Load session" menu item from the menu bar (on the top of the window). You will be prompted to provide the path to the session file, this file (gasch_yeast_hs1_short.zip). Click the "Open" button. Once the data is loaded into BiGGEsTS, you can see that the dataset tree (on the panel on the left) has grown and that it has new items. Read the Quickstart Guide [see Additional file 2], for details on how to use BiGGEsTS. Click here for file

Additional file 6 **Case study: discovering transcriptional modules using BiGGEsTS**. Case study describing how to use BiGGEsTS to discover transcriptional regulatory modules using the transcriptional response of *Saccharomyces cerevisiae *to heat stress. The results published in [6] are reproduced. Click here for file
