# Supplementary material for: BiGGEsTS: integrated environment for biclustering analysis of time series gene expression data
Source: BMC Res Notes. 2009 Jul 7;2:124. doi: 10.1186/1756-0500-2-124 (PMC2720980; doi:10.1186/1756-0500-2-124)
Supplement: Additional file 1 — Multi-platform distribution of BiGGEsTS. A multi-platform distribution of BiGGEsTS. The archive biggests.zip contains a directory with several files, including installation files, the application, sample datasets and sessions, the Quickstart Guide to the software ("BiGGEsTS Quickstart.pdf") and a simple text file with installation instructions ("readme.txt"). In Windows (or Mac OS X) run the "install.bat" (or "install.sh" in Mac OS X) file, for installing the Graphviz dot application and the GO files, and then the "biggests.bat" ("biggests.sh") file, for running the software. For Linux and other operating systems, please install the Graphviz dot application first and edit the "install.sh" file to append the path of the dot executable file (typically /usr/bin/dot) to the last line. Then run the "install.sh" script followed by "biggests.sh". Detailed instructions on how to install and use BiGGEsTS are also available in the Quickstart Guide [see Additional file 2]. The latest version of the software is available at the official website. [file 1756-0500-2-124-S1.zip › biggests_1.0.2/BiGGEsTS Quickstart_1.0.2.pdf]

BiGGEsTS

BiclusterinG Gene Expression Time Series

Quickstart Guide for v1.0.2

BiGGEsTS is a software tool for time series gene expression data analysis, based on biclustering algorithms particularly suited for this kind of data. It is open source and freely available at: http://kdbio.inesc-id.pt/software/biggests/. The purpose of this quickstart document is to provide simple instructions on how to install and use BiGGEsTS. It is written under the assumption that the researcher knows what microarrays and expression data are. However, anyone who wants to try or start using BiGGEsTS without any previous knowledge on these concepts will be able to run the software using this guide. For obtaining help, sending feedback and improvements and/or reporting issues related to BiGGEsTS software, use the following e-mail: biggests.software@gmail.com.

Note that BiGGEsTS is free software: you can redistribute it and/or modify it under the terms of the GNU General Public License as published by the Free Software Foundation, either version 3 of the License, or any later version. BiGGEsTS is distributed in the hope that it will be useful, but WITHOUT ANY WARRANTY; without even the implied warranty of MERCHANTABILITY or FITNESS FOR A PARTICULAR PURPOSE. See the GNU General Public License for more details. A copy of the GNU General Public License is included with BiGGEsTS. For detailed information about the license see the GNU licenses at http://www.gnu.org/licenses/.

Pre-Requisites

If you want to install BiGGEsTS, please check if you have a JVM (Java Virtual Machine) with JDK/JRE 1.5 or higher installed on your system.

We recommend a minimum of 1024 MB of RAM and 300MB of available disk space for installing and running the BiGGEsTS software.

Index

1. Installation 3
2. Running BiGGEsTS
2.1. Menu bar 6
2.2. Loading a dataset 7
2.3. Analyzing matrices 10
2.4. Visualizing hierarchical structure with dendrograms 12
2.5. Preprocessing time series gene expression data 13
2.5.1. Filtering of missing values 14
2.5.2. Filling of remaining missings 14
2.5.3. Normalization 14
2.5.4. Smoothing 14
2.5.5. Discretization 15
2.6. Biclustering time series expression data 17
2.6.1. CCC-Biclustering 17
2.6.2. *e*-CCC-Biclustering 17
2.6.3. CCC-Biclustering and *e*-CCC-Biclustering extensions 17
2.6.4. CC-TSB 18
2.7. Visualizing biclustering results 19
2.8. Analyzing GO terms 24
2.9. Post-processing groups of biclusters 26
3. Formal Definitions 29
3.1. Expression matrix 29
3.2. Bicluster 29
3.3. Coherent biclusters with contiguous columns 29
3.4. Contiguous column coherent biclusters with exact patterns 29
3.5. Contiguous column coherent biclusters with approximate patterns 29
3.6. Sign-changes (anticorrelated patterns) 30
3.7. Gene-shifts (scaled patterns) 30
3.8. Time-lags (time lagged patterns) 31
3.9. Post-processing metrics 32

1. Installation

On Windows, using the installer:

1. Double-click on the installer file and click **Next**.

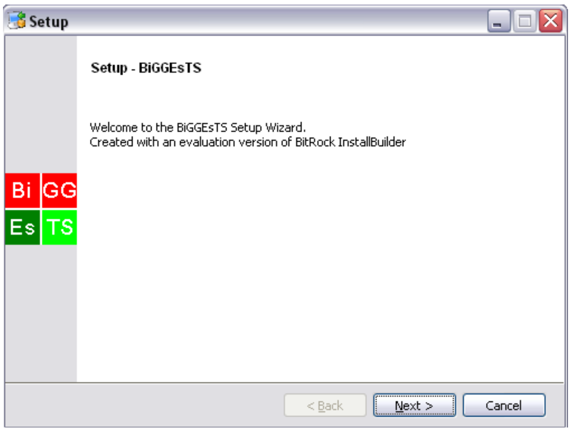

2. After reading the terms of the license, select the ***I accept the agreement*** option and click ***Next***.

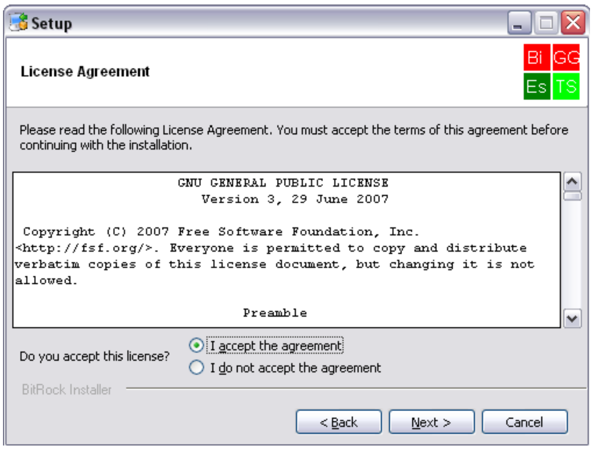

3. Specify the directory where BiGGEsTS should be installed and click ***Next***.

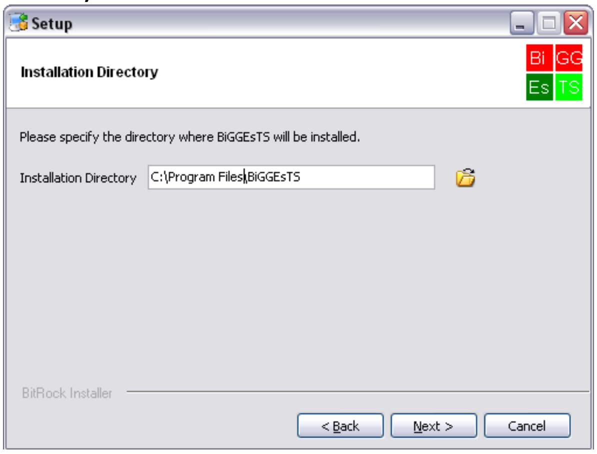

4. After a successful installation you will be prompted to finish the process. You can do this by clicking the ***Finish*** button. BiGGEsTS is now ready to be used. You will find shortcuts to BiGGEsTS on both the **Desktop** and the **Start** menu.

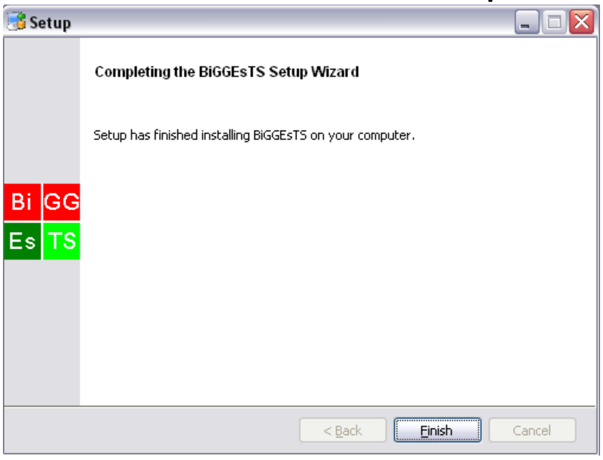

On Windows or Mac OS, using the multi-platform distribution:

1. After downloading the zip or tar.gz file from BiGGEsTS website, decompress it to a suitable location.
2. Execute the installer file inside the resulting directory (double-click the install.bat file on Windows or run the install.sh file on Mac OS). Wait for the installation to conclude.
3. You may then execute BiGGEsTS anytime by executing the biggests script file (double-click the biggests.bat file on Windows or run the biggests.sh on Mac OS).

On Linux and other OSs, using the multi-platform distribution:

1. Install Graphviz on your system. You'll find the source code and binaries, as well as documentation, at http://www.graphviz.org/.
2. Download the zip or tar.gz file from BiGGEsTS website and decompress it to a suitable location.
3. Edit BiGGEsTS installer file (install.sh), appending the path to the dot binary file (usually /usr/bin/dot), preceeded by a space, to the last line (e.g. "java -classpath biggests.jar biggests/utils/BiggestsInstall /usr/bin/dot").
4. Execute the install.sh script file.
5. You may now execute BiGGEsTS whenever by simply running biggests.sh script.

Important notice: When executing BiGGEsTS through biggest.bat or biggest.sh, the parameters of the Java Virtual Machine (JVM) are automatically tuned according to the characteristics of the system in which the application is being executed. In some cases, however, it is not possible to automatically figure a good combination of parameters.

A common problem concerns the object heap size. Specifically, when an error occurs, a message similar to the following is displayed (the values after –Xms and –Xmx may be different):

```
java -Xss5M -Xms1073204k -Xmx1073204k -jar biggests.jar
Error occurred during initialization of VM
Could not reserve enough space for object heap
Could not create the Java virtual machine.
```

The most direct solution to overcome this is to edit the biggest.bat (Windows) or biggest.sh (UNIX) file and replace the Xms and Xmx parameters by values lower than the automatically determined ones (highlighted in green). You should also guarantee that the new values do not exceed the amount of physical memory available in your system. In 32-bit Windows machines with more than 2GB of RAM, these values should not exceed 1500000KB, that is, approximately 1.5GB.

The biggest.bat and biggest.sh files are also available in the version of BiGGEsTS for Windows provided with the installer. They are usually contained in the installation directory (typically C:\Program Files\BiGGEsTS\).

2. Running BiGGEsTS

2.1. Menu bar

The menu bar contains three menus: ***Session***, ***Settings*** and ***Help***. Functionalities accessible via this menu bar can always be performed, regardless of the node and/or the tab selected in the dataset tree and the tabbed panes.

The ***Session*** menu contains the following items:

- ***Load dataset***: it basically selects the ***Loading*** tab.
- ***Load session***: enables loading a session previously saved using the functionality ***Save session***, also available in this menu. The user is always prompted if the current session should be saved before loading a new one.
- ***Save session***: saves a session. The user is prompted for specifying the location for the resulting session file, compressed in a zip file.
- ***Exit***: Prompts the user if the current session should be stored and terminates the application.

The ***Settings*** menu contains the following item:

- ***Color scheme***: enables the selection of one of two color schemes for heatmaps (tables of colors and symbols). Available options are ***Red and green*** and ***Yellow and blue***. The red and yellow and the green and blue colors are used for the higher and lower expression values, respectively, in the corresponding schemes.

The ***Help*** menu contains the ***About…*** item, which displays a popup with information about the application, such as the authors and the version.

2.2. Loading a dataset

When you start BiGGEsTS, the main window looks like this (program starts on the ***Loading*** functionality):

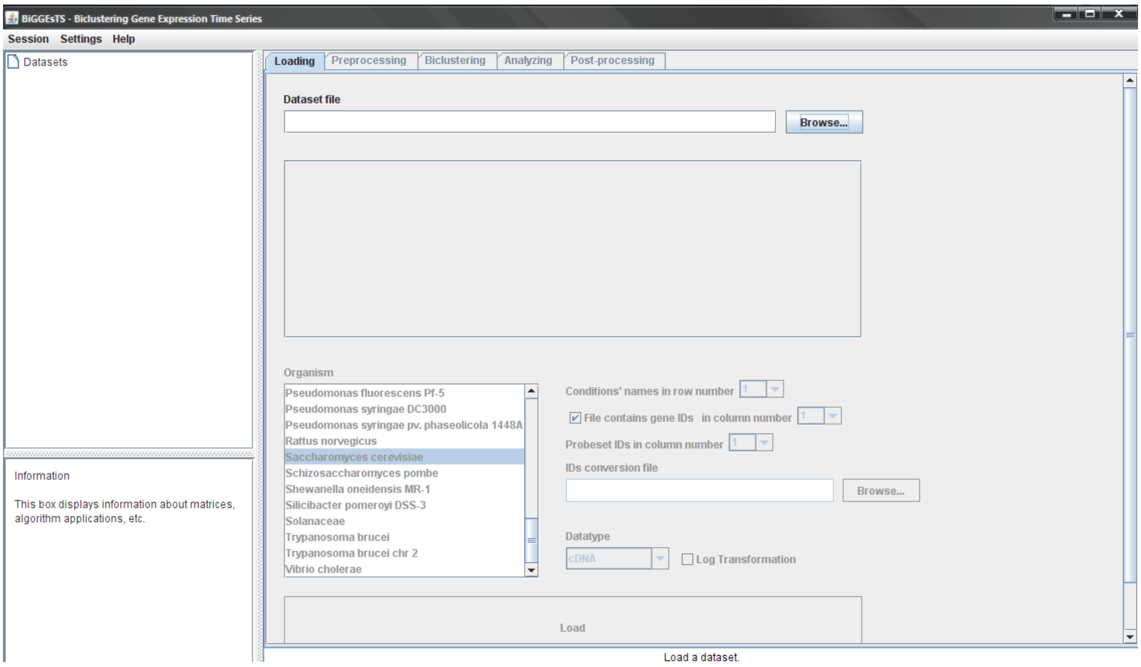

The BiGGEsTS window.

You may want to **load a dataset** from a character delimited text file. Type the **path** to the file to load (or click ***Browse…*** button and select the dataset file in file system instead; sample files are also available in the Datasets directory, the default location for raw time series expression data, within the directory where BiGGEsTS is installed; see the readme.txt file in the same directory for specific details on the contents of these sample files). You'll be presented a preview of the data in your file.

The dataset text file must be structured like this:

	C_1	C_2	C_3	C_4	C_5	C_6	…
G_1	E_{11}	E_{12}	E_{13}	E_{14}	E_{15}	E_{16}	…
G_2	E_{21}	E_{22}	E_{23}	E_{24}	E_{25}	E_{26}	…
G_3	E_{31}	E_{32}	E_{33}	E_{34}	E_{35}	E_{36}	…
…	…	…	…	…	…	…	…

In which each G_X is a name of a gene, each C_Y is a name of a condition (time-point) and each E_{XY} is the expression value of gene G_X in condition C_Y. Values must be delimited by a specific character, which may be a **tab**, regular **space** or a **semicolon** (;). The gene expression data may also have some missing values (blank gaps). BiGGEsTS will deal with them later.

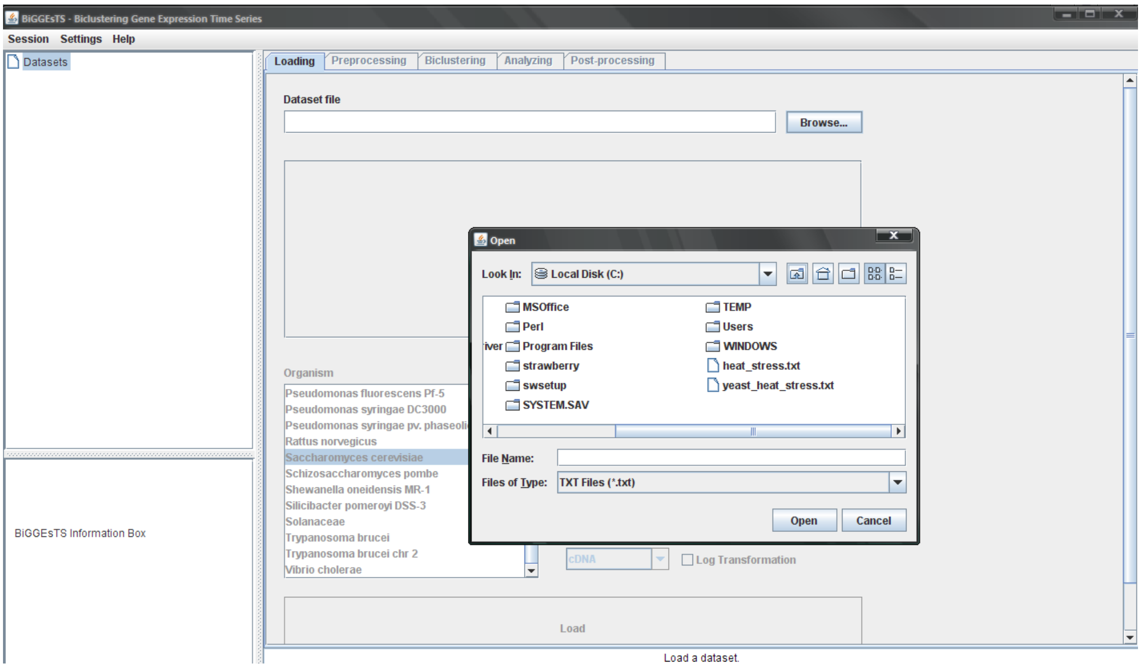

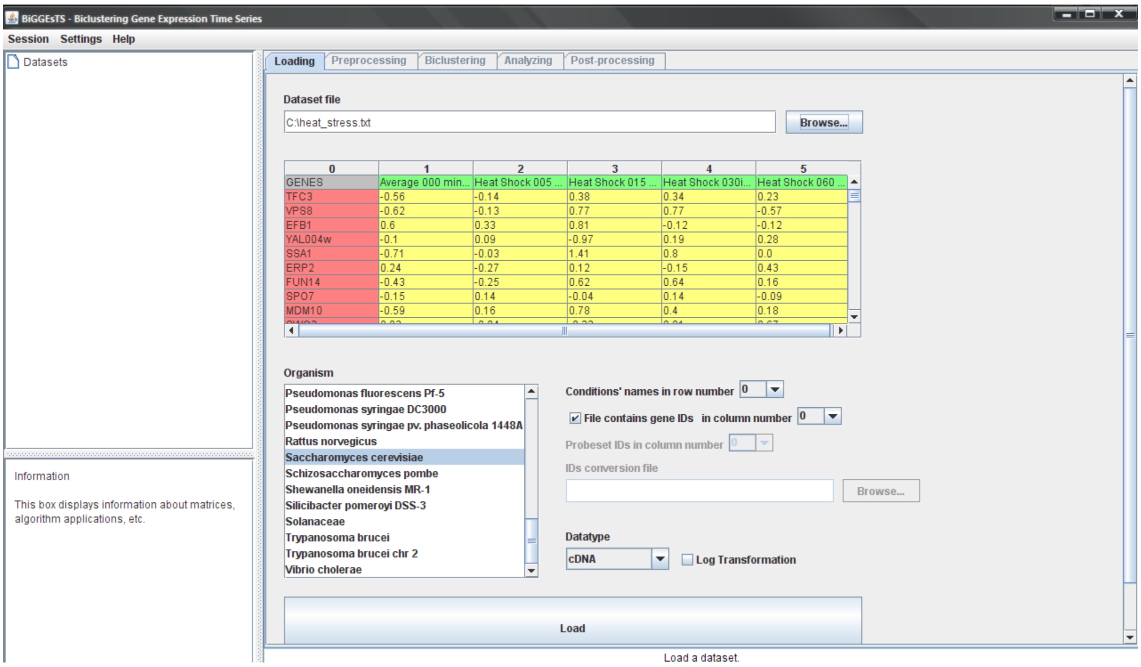

The input of time series gene expression data.

If the names of the genes in your file comply with an identification nomenclature system other than HGNC (HUGO Gene Nomenclature Committee), then you must uncheck the ***File contains gene IDs in column number*** checkbox, specify which column contains the names of the genes and provide an ID conversion file. This should be a character delimited text file (allowed delimiters are the same as the ones for dataset files) containing two columns: the first with the probeset IDs (the names of the genes used in the experiment) and the second with the corresponding HGNC names. You may also use BiGGEsTS with probeset IDs without converting them by leaving the ***File contains gene IDs in column number*** checkbox checked. Just be aware that, if you do this, function analysis won't produce valid results, since such names can't be matched with the ones used in the Gene Ontology files.

Select the number of the row which contains the **names of the experimental conditions** and the correct **data type**.

To perform a **log transformation** on the loaded data, check the ***Log Transformation*** checkbox. In that case, both **Original** and **Preprocessed** matrices containing the original and the log transformed data matrix, respectively, will be added to the tree.

Finally, click the ***Load*** button. BiGGEsTS will check if the Gene Ontology files are available for the organism that you have selected and if that is the case, it retrieves the GO terms that annotate the genes of the loaded dataset. This may take a few seconds but once it is done, one is able to check which GO terms annotate a given gene, by clicking on the corresponding row in the matrix. Note that the GO terms retrieved correspond to the most specific GO terms annotating the gene (before applying the true path rule).

2.3. Analyzing matrices

After loading the sample dataset, a new dataset is created in the dataset tree (on the left side of the window). An **Original** matrix is added to this new dataset, containing the loaded gene expression data. By default, BiGGEsTS shows the colored expression matrix (***Colors*** tab), but you may also visualize the matrix with the original data by clicking on the ***Values*** tab (bottom of the window).

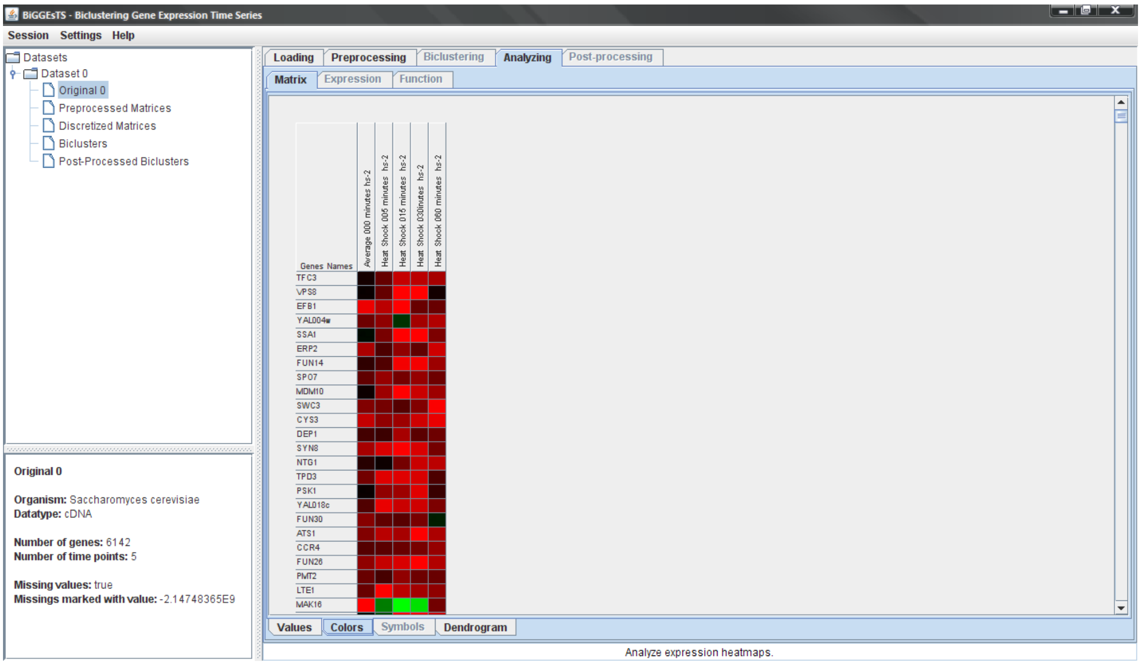

Table of colors for the original matrix. Lower values are colored green or blue, higher values are colored red or yellow and missing values are set to yellow or white, depending on whether the *Red and green* or the *Yellow and blue* color scheme is in use.

All kinds of matrices are **sortable**. This means that you can have the genes (rows) of a matrix sorted by their expression values/symbols for a given condition (column) in increasing or decreasing order. This is done by clicking the cell that corresponds to the condition, that you want the rows of the table to be sorted by, in the header of the table. A first click sorts the values in increasing order, a second one does it in decreasing order and a third causes the rows to return to their original order (resets).

Moving your mouse over one of the cells of the matrix displays a specific **text tip** according to the content of the cell.

Matrices can be **exported as image files**. The access to this functionality is provided via the right button of the mouse. When exporting a matrix as an image, you are prompted to provide a proper file name, location and format (PNG and JPG available).

As mentioned before, BiGGEsTS is able to **extract the GO terms that annotate the genes** of the dataset. If such information is available, it can be obtained by clicking on a row of the table (left button of the mouse). The GO terms that annotate the corresponding gene are then displayed in a popup window, which additionally allows for selecting and copying the text content to paste on other documents.

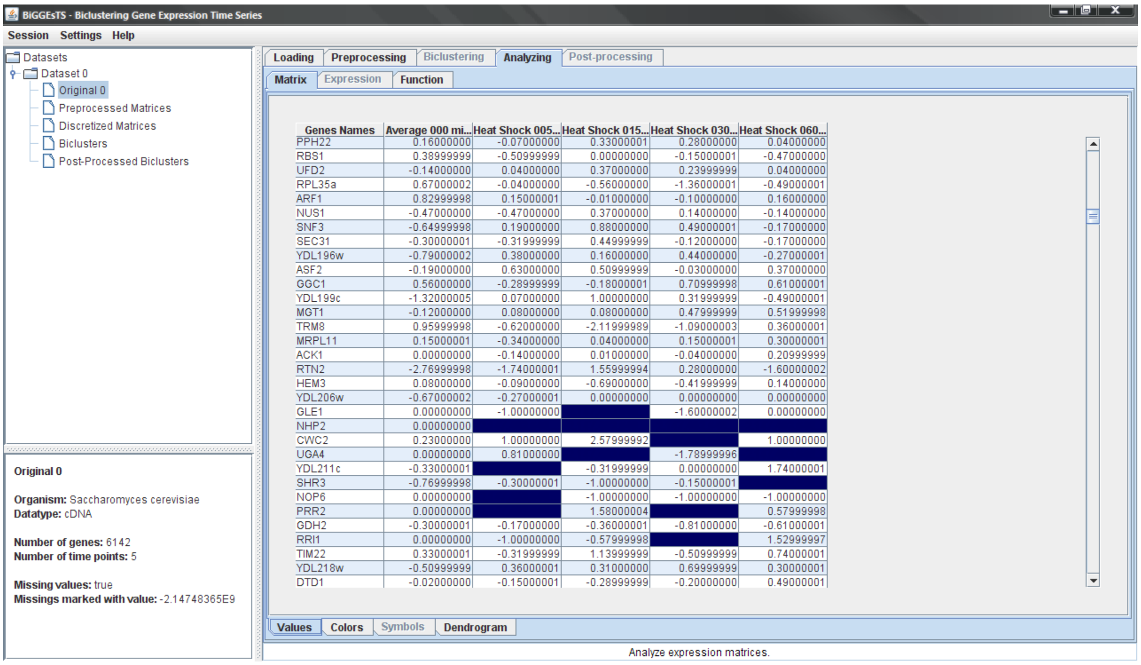

Table of values for the original matrix. Missing values are colored in dark blue.

After having loaded the time series gene expression data, BiGGEsTS is quite intuitive to use. For every operation there are some basic steps to follow: select some data matrix or bicluster in the dataset tree and then choose the functionality that you want to perform on the tabs at the top (and bottom) of the window. The **input of gene expression data is always available** for every selected node in the dataset tree. Below is a summary of the additional main features, available upon the selection of a given type of node in the dataset tree together with a given set of tabs:

Original or **Preprocessed** matrices – (i) Preprocessing; (ii) Biclustering (CC-TSB, only available if the matrix has no missing values); (iii) Analyzing -> Matrix -> Values; (iv) Analyzing -> Matrix -> Colors; (v) Analyzing -> Matrix -> Dendrogram.

Discretized matrices – (i) Biclustering (CCC-Biclustering and e-CCC-Biclustering); (ii) Analyzing -> Matrix -> Values; (iii) Analyzing -> Matrix -> Colors; (iv) Analyzing -> Matrix -> Symbols; (v) Analyzing -> Matrix -> Dendrogram.

Biclusters Group or **Post-Processed Biclusters Group** – (i) Analyzing -> Matrix -> Values; (ii) Analyzing -> Matrix -> Colors; (iii) Analyzing -> Matrix -> Symbols (only for groups of biclusters obtained from discretized matrices); (iv) Analyzing -> Expression -> Bicluster time-points; (v) Analyzing -> Expression -> Bicluster pattern (only for groups of biclusters obtained from discretized matrices); (vi) Analyzing -> Function -> Table; (vii) Post-Processing.

Bicluster – (i) Analyzing -> Matrix -> Values; (ii) Analyzing – Matrix -> Colors; (iii) Analyzing -> Matrix -> Symbols (only for biclusters obtained from discretized matrices); (iv) Analyzing -> Expression -> Bicluster time-points; (v) Analyzing -> Expression -> All time-point; (vi) Analyzing -> Expression -> Bicluster pattern (only for biclusters obtained from discretized matrices); (vii) Analyzing -> Function -> Table.

2.4. Visualizing hierarchical structure with dendrograms

Hierarchical clustering can be useful for deriving hierarchical relationships based on the degree of similarity between the elements of the gene expression data, either genes or conditions. This technique is available via the ***Analyzing***, ***Matrix*** and ***Dendrogram*** tabs, selected in this order.

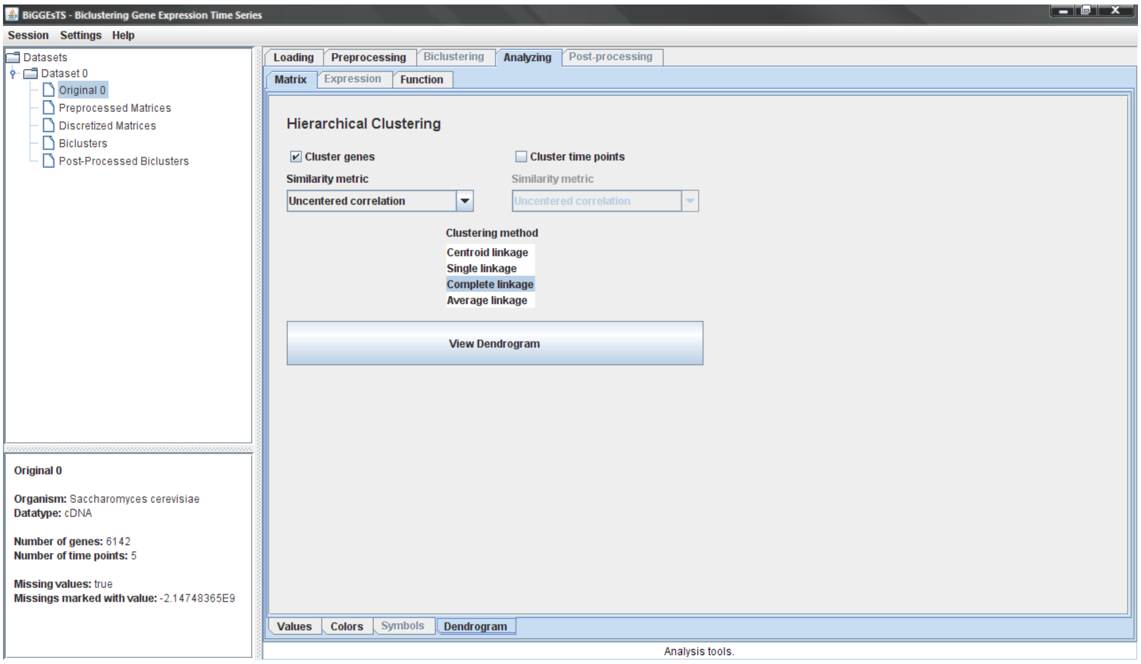

Analyzing matrix dendrogram panel with the options for selecting and applying a hierarchical clustering algorithm to the gene expression data.

The clustering of both genes and conditions dimensions is available. We however note that when analyzing time series gene expression data, clustering conditions is not very meaningful, since they correspond to consecutive instants of time. As in the Cluster 3.0 software (http://bonsai.ims.u-tokyo.ac.jp/~mdehoon/software/cluster/software.htm), BiGGEsTS lets you select the metric used for measuring the similarity between the elements of the gene expression matrix (either genes or conditions), either based on their correlation or distance, from the following list: (i) uncentered correlation, (ii) Pearson's correlation, (iii) uncentered absolute correlation, (iv) Pearson's absolute correlation, (v) Spearman's rank correlation, (vi) Kendall's tau correlation, (vii) Euclidean distance, and (viii) Cityblock distance (for details on these distances see http://bonsai.ims.u-tokyo.ac.jp/~mdehoon/software/cluster/manual/Distance.html). You may also specify the approach followed by the algorithm when computing the cluster-pairwise similarity: (i) centroid linkage, (ii) single linkage, (iii) complete linkage, and (iv) average linkage (for details on these techniques see http://bonsai.ims.u-tokyo.ac.jp/~mdehoon/software/cluster/manual/Hierarchical.html).

Once you have set up the correct parameters and clicked the ***View Dendrogram*** button, the results of the hierarchical clustering analysis are presented in a dendrogram displayed by a new Java TreeView window (for details on this application, please visit the official site of Java TreeView: http://jtreeview.sourceforge.net/).

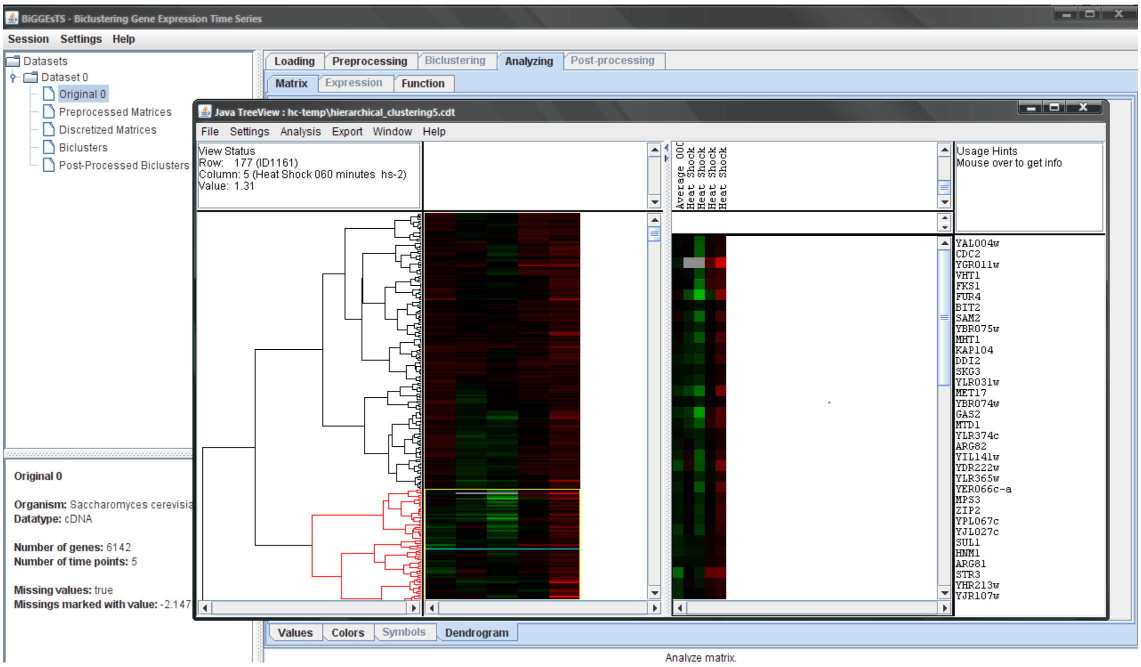

A dendrogram displayed (on the left) by the Java TreeView window superimposed on the BiGGEsTS window. The figure further shows a cluster of genes selected (highlighted in red in the hierarchical structure, on the left). The cluster itself is displayed in the panel on the middle. The list of genes included in the cluster is displayed in the panel on the right.

2.5. Preprocessing time series gene expression data

The next step we want to exemplify involves the preprocessing of the **Original** expression matrix. Select the ***Preprocessing*** tab (on the top of the window), after having the matrix that you want to preprocess selected on the dataset tree. Preprocessing includes the following techniques: (i) **gene filtering**, for filtering genes with missing values and only available for matrices with missing values; (ii) **missing values filling**, for filling missing data with real values; (iii) **data normalization**, to compensate for systematical differences between data measured by the several microarrays/conditions; (iv) **smoothing**, for reducing the impact of the noise in the analysis; and (v) **discretization**, for reducing the infinite set of real gene expression values to an adequate range of discrete values. Note that the data normalization, smoothing and discretization techniques can be applied to matrices with missing values with no previous treatment, because they use an appropriate approach to minimize the impact of the missing values. Once you have selected the preprocessing techniques and set their corresponding parameters, you can click the ***Apply*** button.

Upon the selection of any or several of the first four preprocessing options (filter, fill, normalize, smooth) and disabling of the discretization one, only a **Preprocessed** node is added to the dataset tree. If the discretization is enabled, an additional **Discretized** node is also created and added to the dataset tree. When selecting more than one preprocessing option, the several options are applied one at a time by BiGGEsTS to the gene expression data following the order of the options displayed, from top to bottom, in the preprocessing panel.

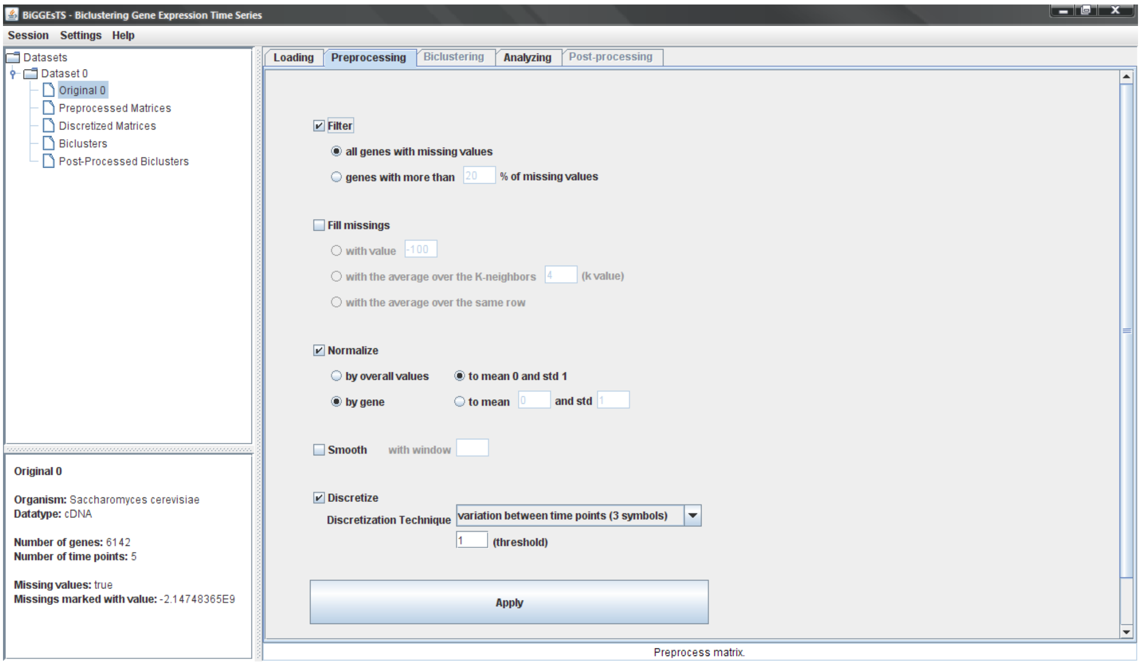

Preprocessing panel displaying the preprocessing options.

The names of the preprocessing steps are quite explicit, but we also list them here, including a brief description of their parameters:

Filtering of missing values

Includes two options for removing genes (rows of the matrix) with missing values: (i) **all genes with missing values** eliminates all rows which contain missing values; (ii) **genes with more than x % of missing values** eliminates all rows whose percentage of missing values exceeds the value of x.

Filling of remaining missings

Provides three alternatives to fill the values missing from the expression matrix with: (i) a given **value**, which has to be typed in the corresponding text field; (ii) the **average of the values of the k-neighbor cells** of the same gene (row); (iii) the **average over all the values of the same gene** (row).

Normalization

Normalizes the expression values. They can be normalized altogether using the (i) **by overall values** option to mean 0 and standard deviation 1 or to a given mean and standard deviation, which have to additionally be specified in the corresponding text fields; or by row using the (ii) **by gene** option to mean 0 and standard deviation 1.

Smoothing

Acts like a low-pass filter for attenuating the negative effect of outliers; requires a parameter: the **length of a window** of neighbors to consider when computing the new value for substituting an outlier (note that you must provide an odd value, since the window includes the outlier value).

Discretization

Provides a number of different discretization techniques for replacing each absolute expression value by a symbol of a given alphabet. Alphabets of two or three symbols are the most common, containing the symbols {D, U} and {D, N, U}, respectively, where D means down-regulation, N is no-regulation and U means up-regulation. A brief description of the available methods follows. For more details see [1].

A first group of methods may be applied to the **overall values** of the matrix or **by gene** and computes the corresponding discrete value for each real element based on its absolute expression value:

(i) **Expression average** is a binary discretization (alphabet {D, U}). Each element of the expression matrix is transformed into a D, if its absolute value is lower than the mean, or into a U otherwise. The mean value can be computed using all the expression values in the matrix or by gene and is calculated by summing all the values and dividing by their number.

(ii) **Mid-range** is also a binary discretization, similar to expression average, except for the fact that the mid-range expression value is used as the criterion for choosing the symbol, instead of the mean. The mid-range is obtained by subtracting the minimum expression value to the maximum and dividing by two.

(iii) **Max-minus percent-max** is also a binary discretization, similar to the previous two. In this case, the criterion for choosing the discrete symbol is computed as the difference between the maximum expression value and a given percentage of it. Such percentage must be specified by the researcher in the application.

(iv) **Equal frequency** can be applied with an alphabet containing an arbitrary number of symbols. It selects the symbols in such a way that, after discretization, each symbol contains the same number of occurrences in the matrix.

(v) **Equal width** can also be applied to an alphabet containing an arbitrary number of symbols. It divides the range of expression values, between the minimum and the maximum values in the matrix, into as many equal width intervals as the number of symbols in the alphabet. Then, every expression value in the matrix is substituted by the corresponding symbol.

(vi) Expression **mean and standard deviation** uses an alphabet of three symbols {D, N, U} and a parameter alpha defined by the researcher. Symbol D is used to replace the expression values below the difference between the mean value and the product of alpha and the standard deviation. U is used for expression levels higher than the sum of the mean value and the product of alpha and the standard deviation. N is used for the remaining expression values.

The second group of methods computes the discrete values based on the variation of the expression level between every pair of consecutive conditions:

(vii) **Transitional state discrimination** uses a binary alphabet {D, U}. In a first step, each value in the matrix is normalized to a z-score, computed as the difference between the expression value and the mean then divided by the standard deviation value. Each element in the matrix is then replaced by an U if the difference between its z-score and the z-score in the same row and previous condition exceeds 0. Otherwise, it gets the symbol D.

(viii) **Variation between time points** can be used with an alphabet of two or three symbols, {D, U} or {D, N, U}, respectively. In the binary case, a parameter alpha must be specified by the researcher. A threshold is calculated as the product of alpha and the standard deviation of the expression values of all genes in time point 0. Then, each element in the matrix is replaced by a U if the difference between its expression value and the value in the same row and previous condition exceeds the computed threshold. Otherwise, it gets the symbol D. In the case of the three-letter alphabet, the threshold is directly chosen by the researcher in the application. Each element in the matrix is then replaced by a U if the difference between its expression value and the value in the same gene and previous time point are higher than the threshold, by a D if such difference is lower than the symmetric of the threshold value, and N otherwise.

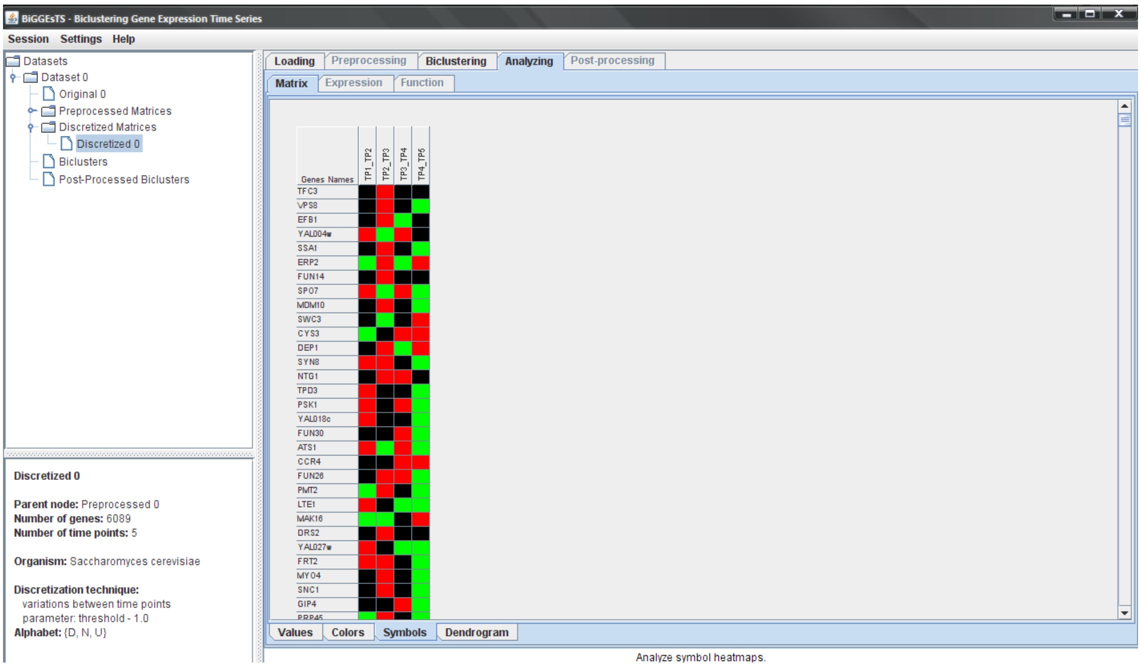

Heatmap of a discretized matrix (obtained from the discrete symbols instead of the real values). You may check which symbol is actually contained in each cell by moving the mouse over it (text tip).

2.6. Biclustering time series expression data

Accessing the ***Biclustering*** tab, you are able to choose the biclustering algorithm and parameterize it.

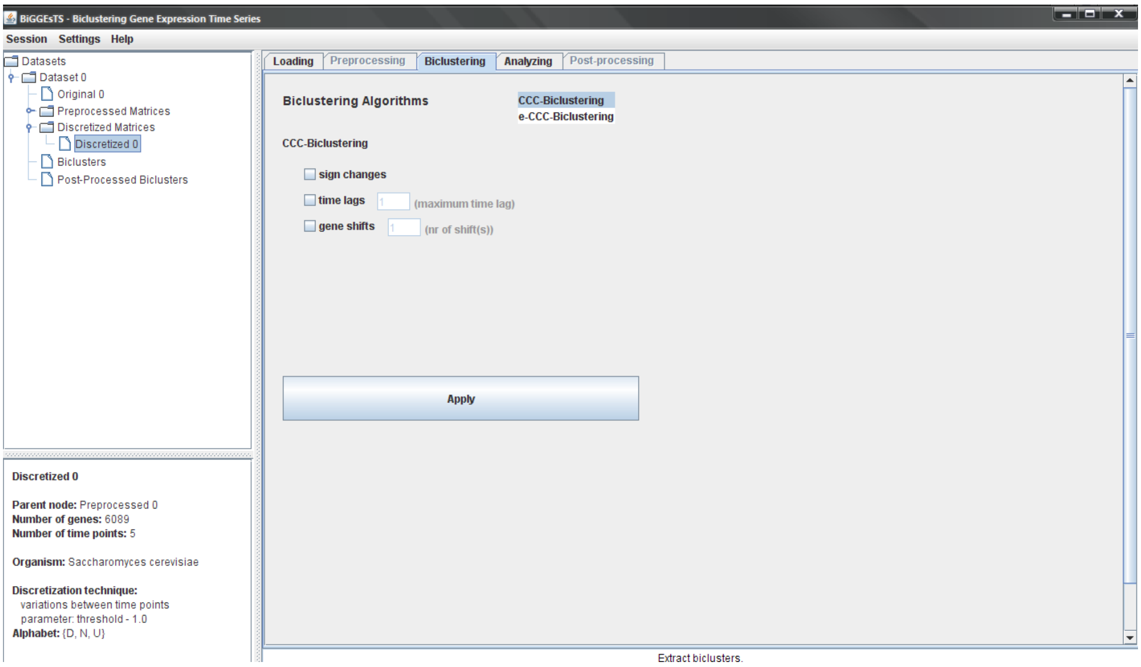

CCC-Biclustering with the default parameterization.

BiGGEsTS integrates three biclustering algorithms: one for real data with no missing values, CC-TSB-Biclustering; and two for discretized matrices that are also able to compute in the presence of missing values, CCC-Biclustering and e-CCC-Biclustering. In our example, we will apply the CCC-Biclustering algorithm to the previously mentioned **Discretized** matrix. We select the CCC-Biclustering algorithm, take the default parameterization and click the ***Apply*** button. Next, we also apply the CCC-Biclustering algorithm, but this time enabling the sign-changes option.

The available biclustering algorithms and parameterizations are:

> **CCC-Biclustering** – for finding biclusters with exact expression patterns in discrete data; when in the presence of missing values, the algorithm uses an appropriate approach for disregarding them from the analysis of the gene expression values.
>
> ***e*-CCC-Biclustering** – for finding biclusters with approximate expression patterns (maximum of *e* errors) in discrete data; required parameters: the **maximum number of errors** allowed per pattern; when activated, the **restricted errors** variation considers as valid errors only the substitutions of symbols which are on a given neighborhood in the alphabet of discretization. When in the presence of missing values, the algorithm may follow one of two approaches: ignore them, as in the case of the CCC-Biclustering algorithm, or consider them as errors. The default behavior is to ignore the missing elements.

Common variations of both CCC-Biclustering and *e*-CCC-Biclustering:

(i) **gene-shifts**, for grouping in biclusters genes with similar expression evolutions, but at different expression levels; parameter: the **number of shifts**, that is, of different expression levels to consider;

(ii) **sign-changes**, for including in biclusters genes with symmetric expression patterns;

(iii) **time-lags**, for considering in the same biclusters genes with similar expression evolutions, but starting at different points in time, in a predefined order as generated by temporal programs; parameter: the **maximum time lag** allowed between expression patterns;

Note: gene-shifts cannot be combined with sign-changes or time-lags.

CC-TSB – for finding biclusters with approximate expression patterns in real data; parameters: (i) **delta**, the threshold for the MSR of each bicluster; (ii) **alpha**, the ratio between the MSR of a row and the MSR of the matrix; (iii) the **number of biclusters to extract**; (iv) the **maximum number of iterations**.

Note that the computation may take time, especially when a large matrix is involved. Sometimes, if the dimension of the matrix is really demanding, computation may also end up aborting, usually due to memory issues.

2.7. Visualizing biclustering results

Upon a successful application of a biclustering algorithm, a group of biclusters is added to the tree, the ***Analyzing***, ***Matrix*** and ***Colors*** tabs are selected and the colored matrices of the first 5 biclusters of the group are displayed by default. This number can be changed using the combo box available in the ***Colors*** panel. The matrices of values and symbols are accessed by selecting the ***Values*** or ***Symbols*** tab, respectively.

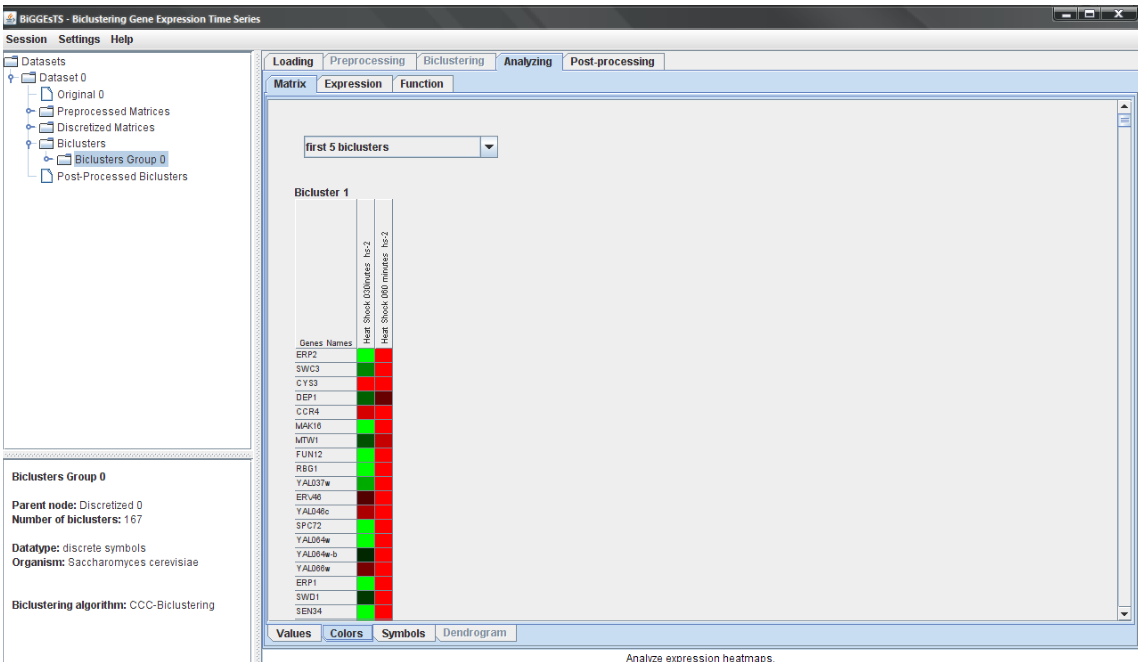

Heatmap (matrix of colors) of the first 5 CCC-biclusters (only the first matrix is visible).

BiGGEsTS enables the display of miniaturized expression charts of biclusters in a group.

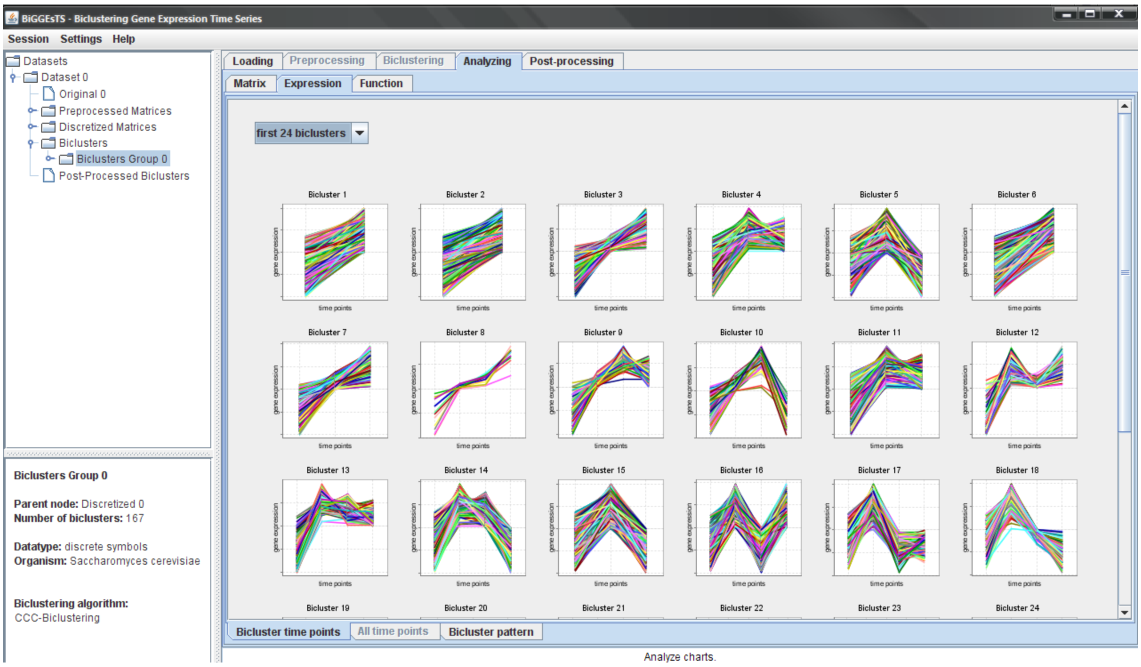

Miniatures of the time-points charts of the first 24 CCC-biclusters identified using the default CCC-Biclustering algorithm.

The expression charts and pattern charts are available upon selection of the ***Analyzing***, ***Expression***, ***Bicluster time-points*** tabs (in this order) and the ***Analyzing***, ***Expression***, ***Bicluster pattern*** tabs (in this order), respectively. Note that when biclusters are large, that is, composed of many genes and/or conditions, or when you are trying to display the charts of a considerable number of biclusters in a group, BiGGEsTS may take some time to draw all these data. By default, only the first 12 biclusters are displayed. You may change this number in the corresponding combo box on the top of the panel.

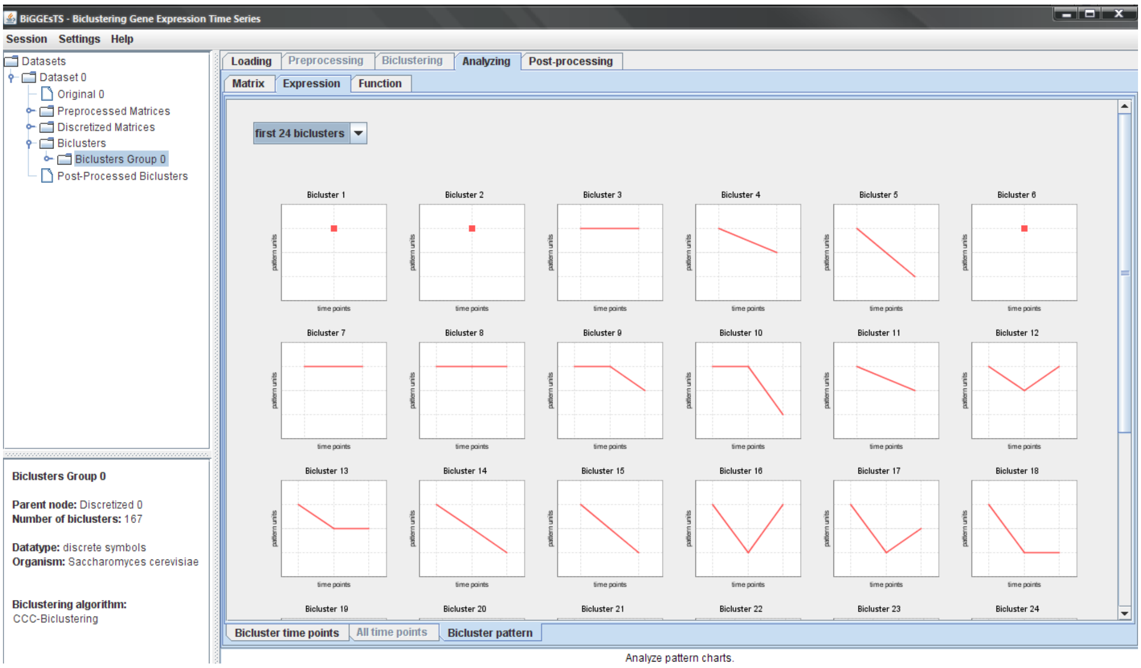

Miniatures of the pattern charts of the first 24 biclusters identified using the default CCC-Biclustering algorithm.

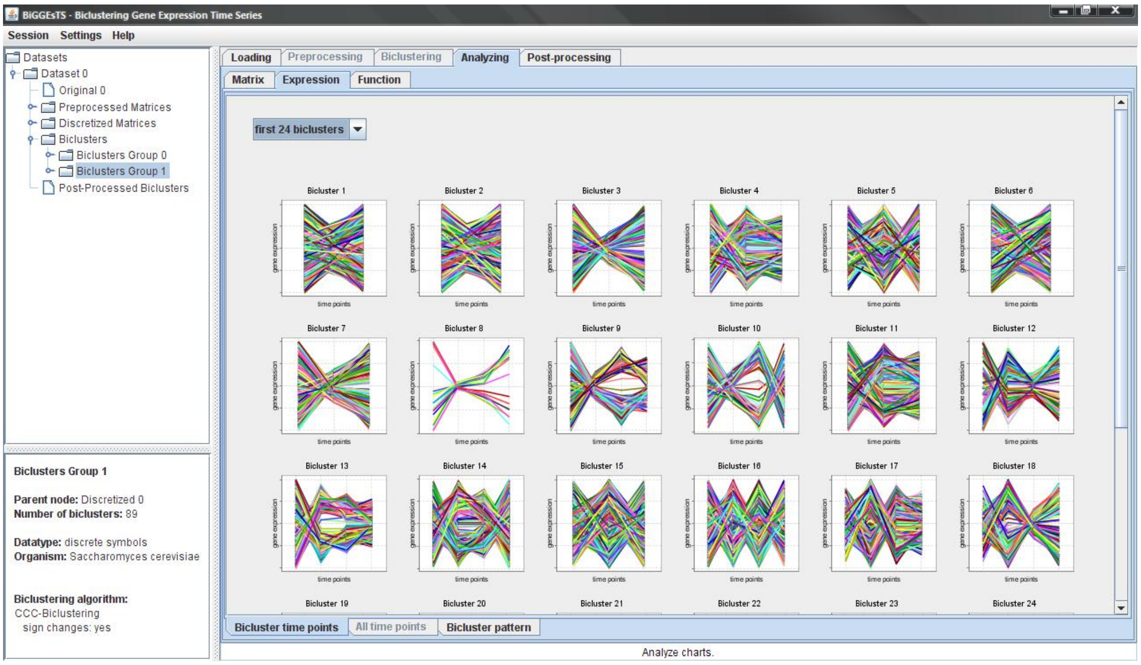

Miniatures of the pattern charts of the first 24 biclusters identified by CCC-Biclustering with sign-changes (anticorrelated patterns).

To access the individual biclusters you must open the folder of the group of biclusters in the dataset tree (try clicking the “key” on the left of the group folder). Once the group is opened you may select a specific bicluster and view its corresponding information. Matrices of values, colors and symbols are displayed in a similar fashion to the one used for original, preprocessed and discretized matrices. Bicluster expression charts, all time-points expression charts and bicluster pattern charts are upon selection of the ***Bicluster time-points***, ***All time-points*** and ***Bicluster pattern*** tabs, respectively (after selecting ***Analyzing*** and ***Expression*** tabs).

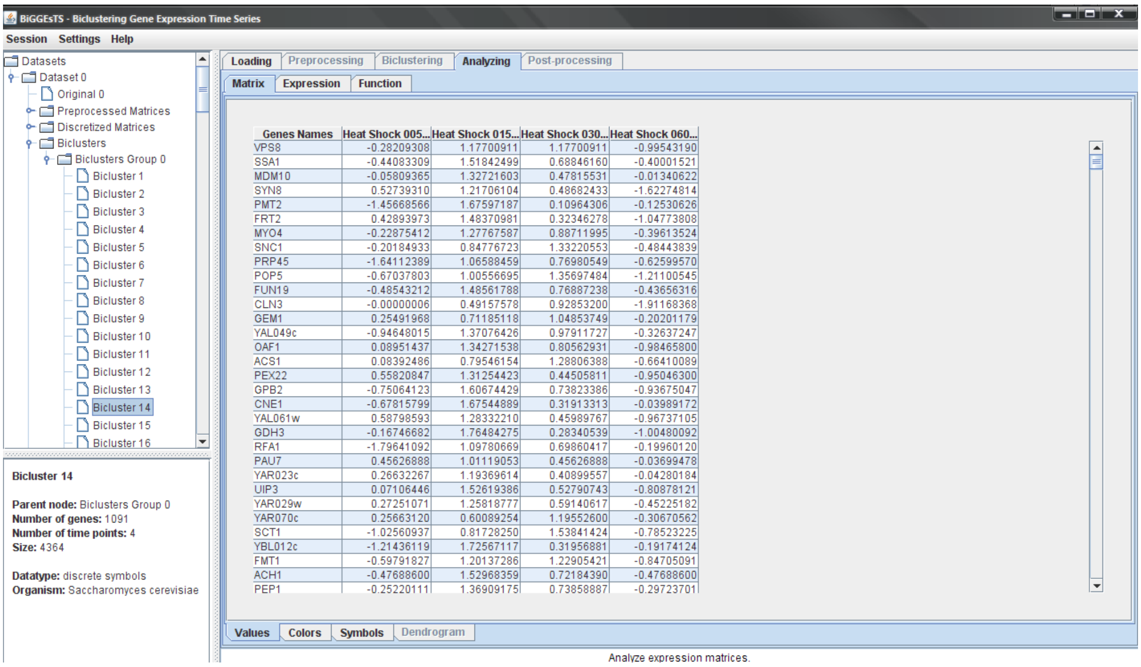

Table of values of the bicluster 14 identified using the default CCC-Biclustering algorithm.

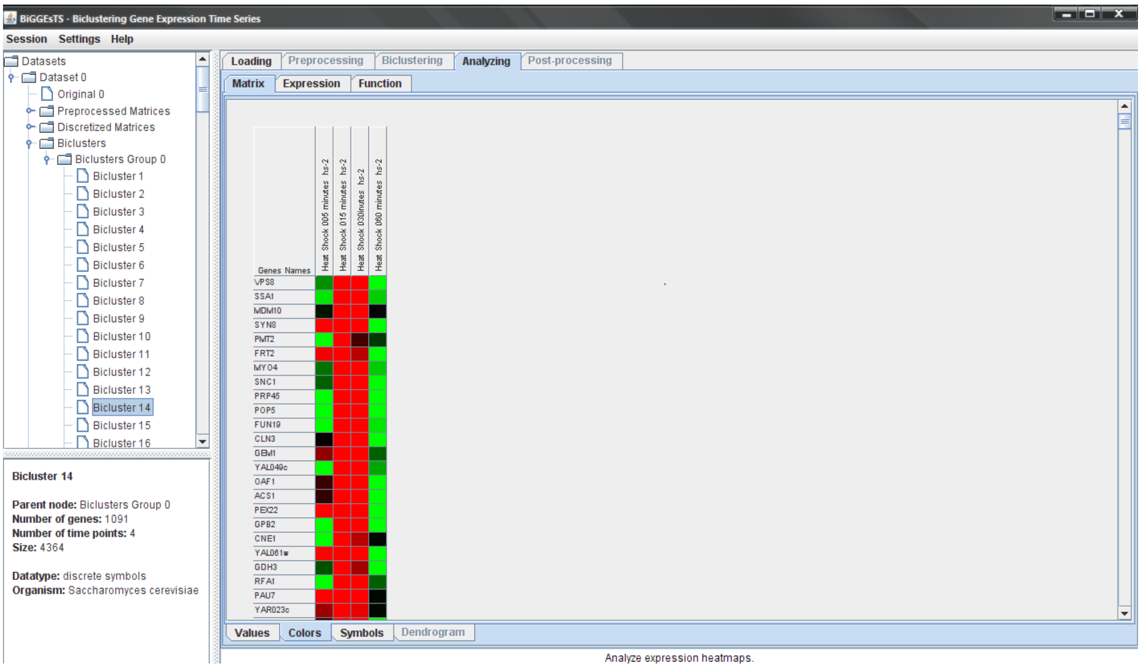

Table of colors of the bicluster 14 identified by the default CCC-Biclustering algorithm.

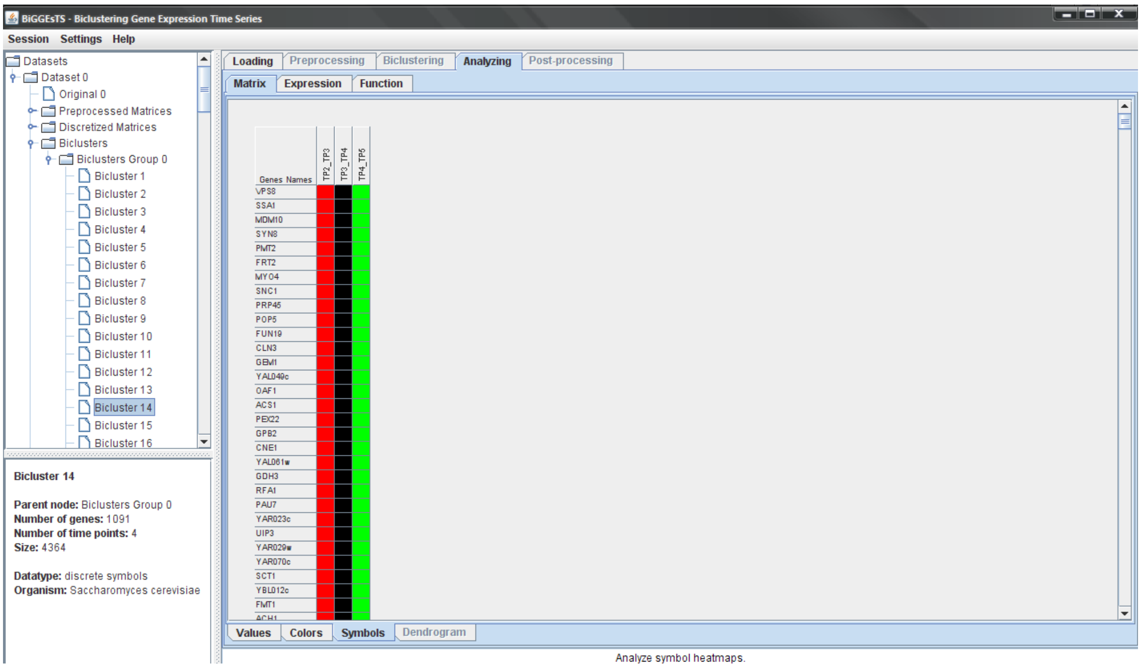

Table of symbols of the bicluster 14 identified using the default CCC-Biclustering algorithm.

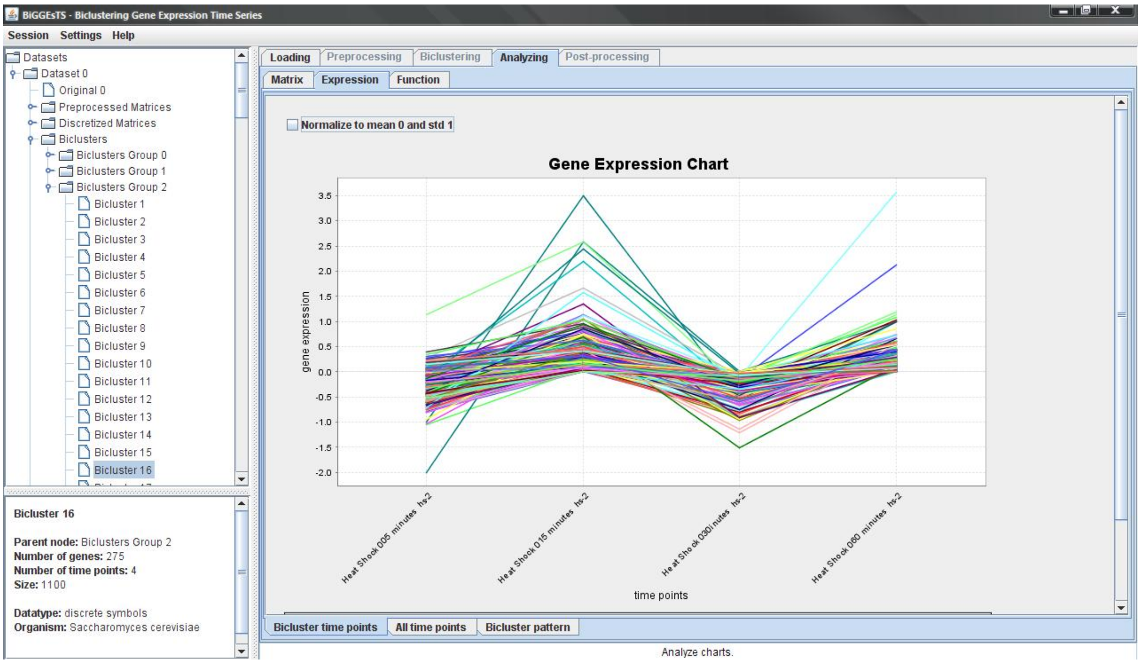

Expression chart showing the evolution of the expression level of the genes in the CCC-bicluster 16 (obtained using the CCC-Biclustering on another matrix resulting from the discretization of unnormalized expression data) along the corresponding conditions of the bicluster. It is possible to normalize the expression levels by checking the ***Normalize to mean 0 and std 1*** checkbox.

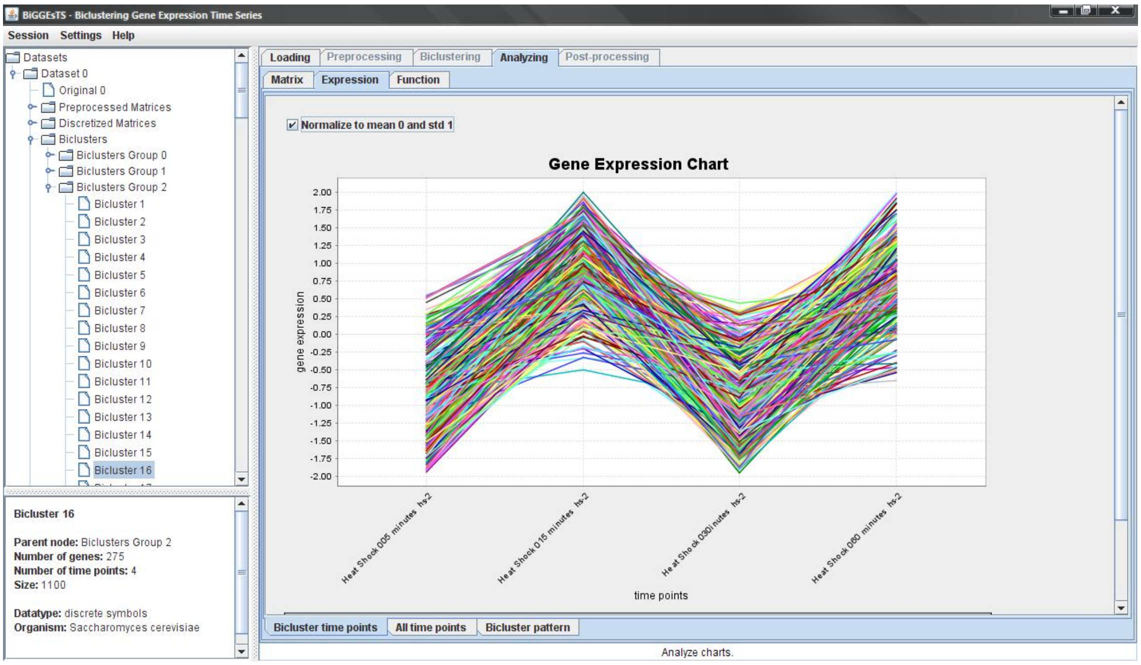

Expression chart showing the evolution of the expression level of the genes in the CCC-bicluster 16 along all the conditions of the dataset. It is possible to normalize the expression level by checking the ***Normalize to mean 0 and std 1*** checkbox.

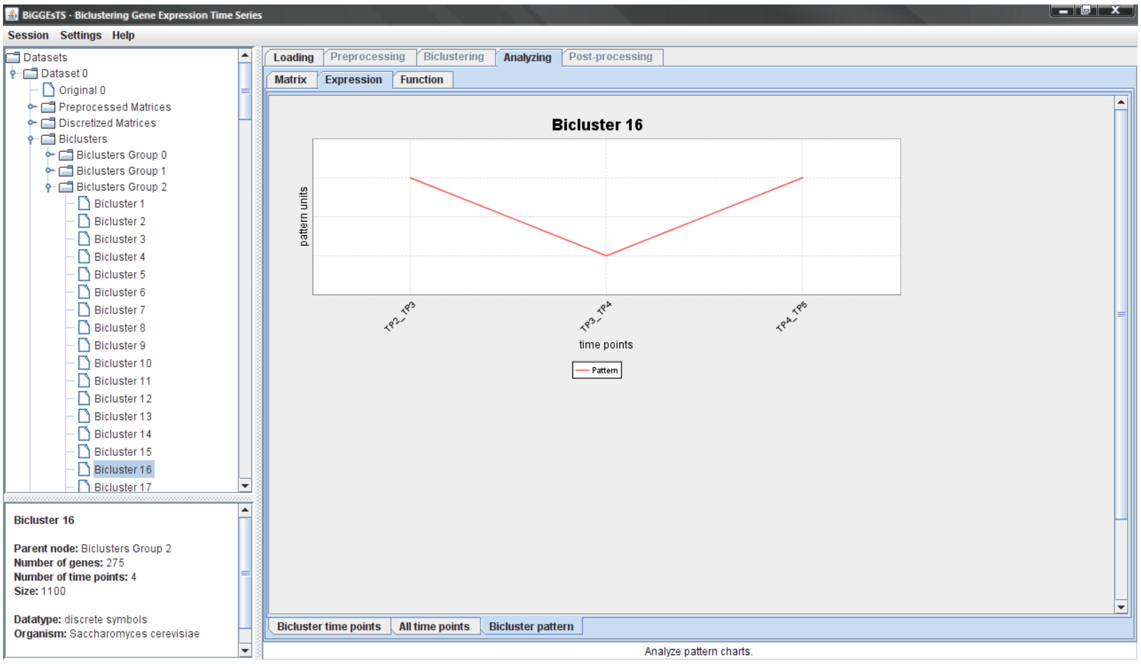

Pattern chart displaying the expression pattern of the CCC-bicluster 16.

All charts, except the miniature ones, allow **saving as image**, **printing** and **zooming**. These options can be accessed by clicking the right button of the mouse on the chart.

2.8. Analyzing GO terms

BiGGEsTS automatically extracts the GO terms that annotate the genes of the dataset when the Gene Ontology files are available. For biclusters and groups of biclusters, it is additionally possible to perform functional enrichment using the term-for-term analysis, which computes the statistical significance of each GO term that annotates the genes in the biclusters. The term-for-term analysis is available by selecting the ***Analyzing***, ***Function*** and ***Table*** tabs in this order.

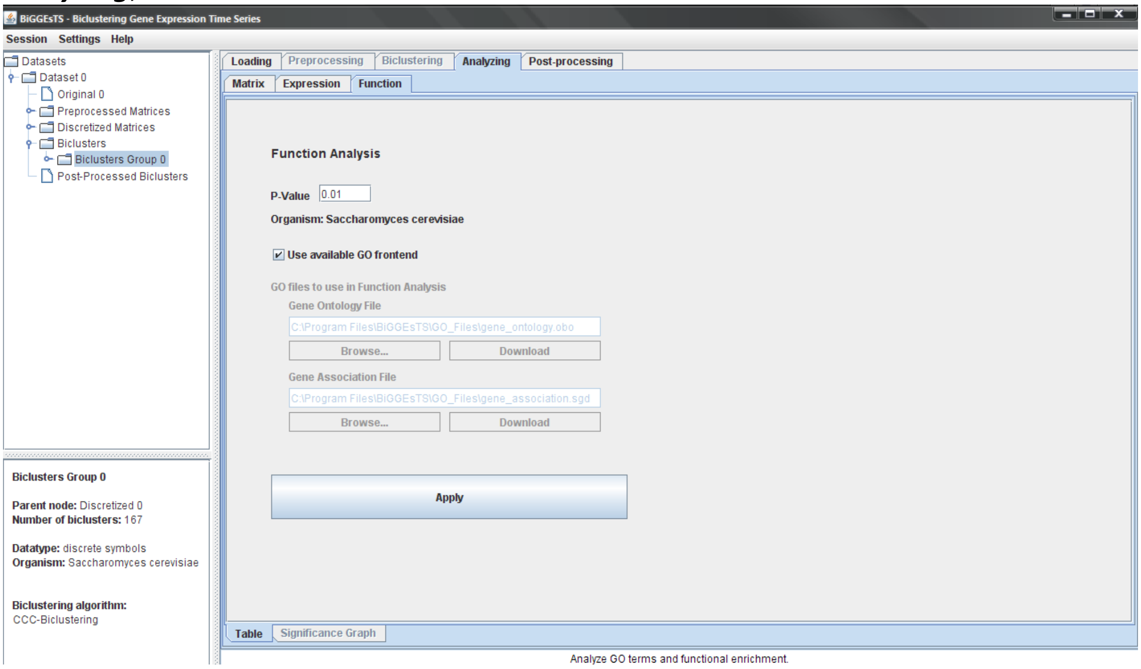

Parameters for the term-for-term analysis.

The required parameters for term-for-term analysis are: a **p-value**, the threshold below which the Bonferroni corrected p-values computed for the GO terms are considered statistically significant; the general **ontology** and specific organism **annotation files**. If these files are available at the proper location (the GO_Files directory within the BiGGEsTS installation directory), their corresponding file paths are displayed in the text boxes. Also note that when this is the case, most likely BiGGEsTS has already used these files to extract the GO terms that annotate the genes in a previous step. When such happens, the ***Use available GO frontend*** check box appears selected, which means that the annotations have already been retrieved and the term-for-term analysis can use them, avoiding to repeat the parsing of the Gene Ontology files. This accelerates the computation process of the term-for-term analysis.

Additionally, BiGGEsTS enables downloading the files from the Gene Ontology repository by clicking the ***Download*** button below the corresponding file path text box.

The results of the term-for-term analysis are displayed in a table. Significant and highly significant terms are highlighted in green. The threshold for statistical high significance is always 0.01. The one for statistical significance is also 0.01 by default, but can be changed to a different value.

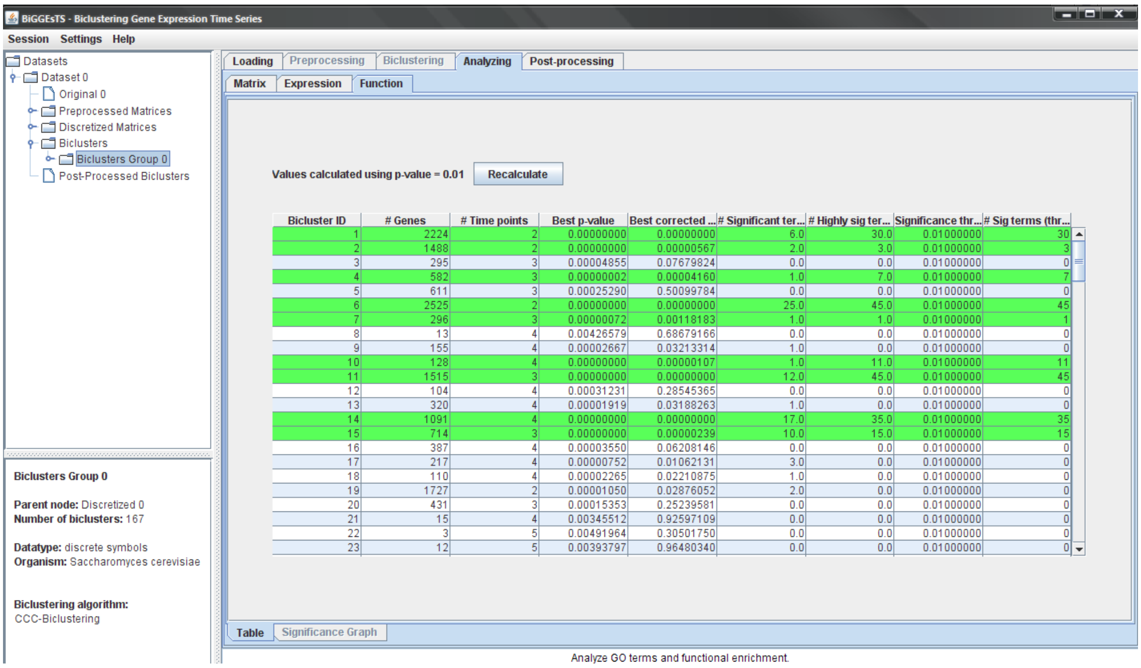

Results of the term-for-term analysis applied to the genes in the group of biclusters. It's possible to recalculate the significance of the GO terms using different Gene Ontology files or p-value threshold by clicking the **Recalculate** button. Clicking a row of this table will select the corresponding bicluster in the dataset tree and redirect the content of the panel to the results of the following selection of tabs: Analyzing, Matrix, Colors.

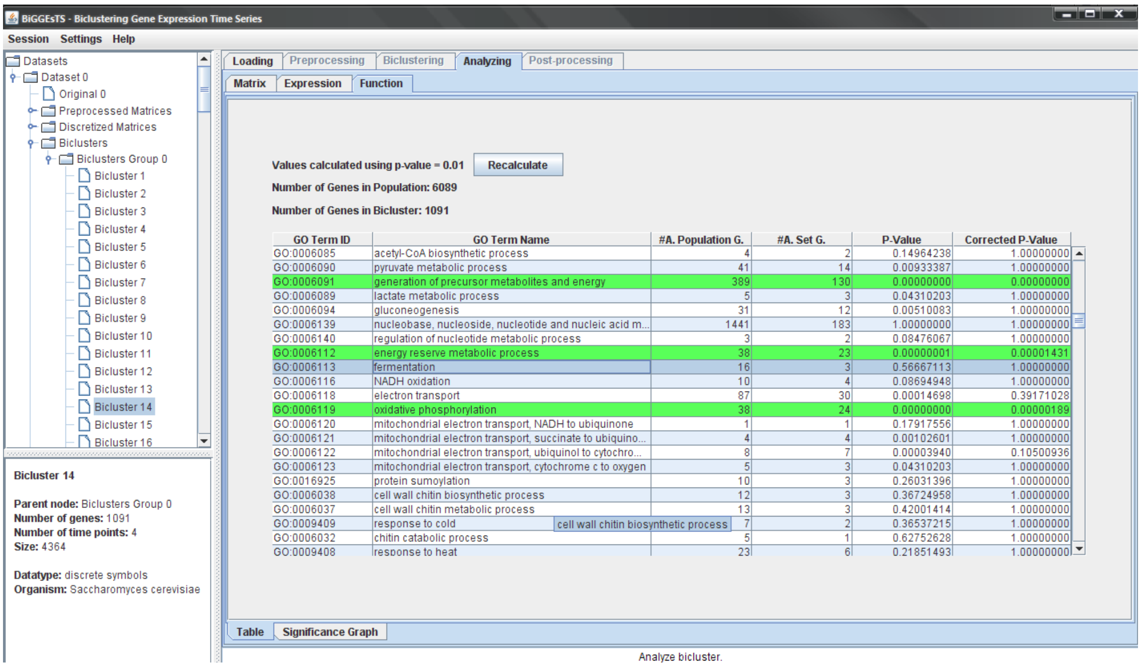

Results of the term-for-term analysis applied to the genes in the bicluster 14.

When the term-for-term analysis reveals significant functions (highlighted in green), the ***Significance Graph*** tab is enabled and the corresponding panel displays a graph with the ontology of the GO terms. The significant terms are highlighted in green, yellow or purple depending on which of the main GO term they specialize from. You may further **zoom** the graph or **save** it as a **PNG** or **SVG** image.

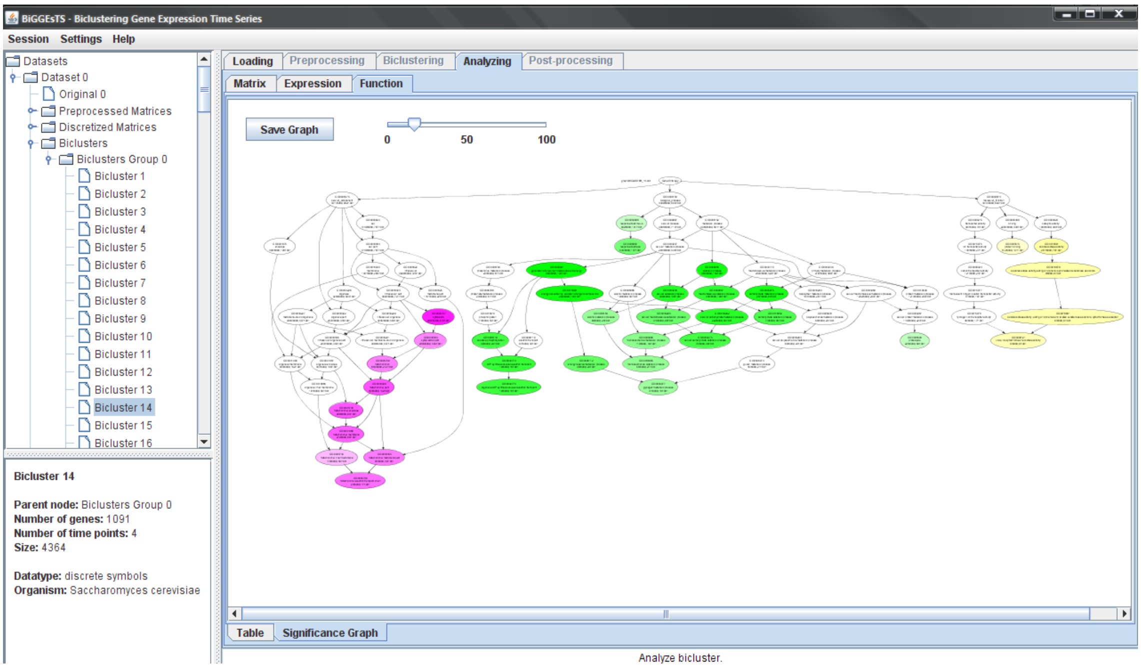

Graph of significant GO terms extracted by the term-for-term analysis for the genes in the bicluster 14.

2.9. Post-processing groups of biclusters

The groups of biclusters can be post-processed (***Post-Processing*** tab). This operation consists in filtering and/or sorting the biclusters of a group of biclusters based on specific criteria, available for selection in the post-processing panel. A short description of each filtering/sorting criterion and parameters is provided below the corresponding list in the panel, upon the selection of a given item. To apply the post-processing operations to the data click on the ***Apply*** button.

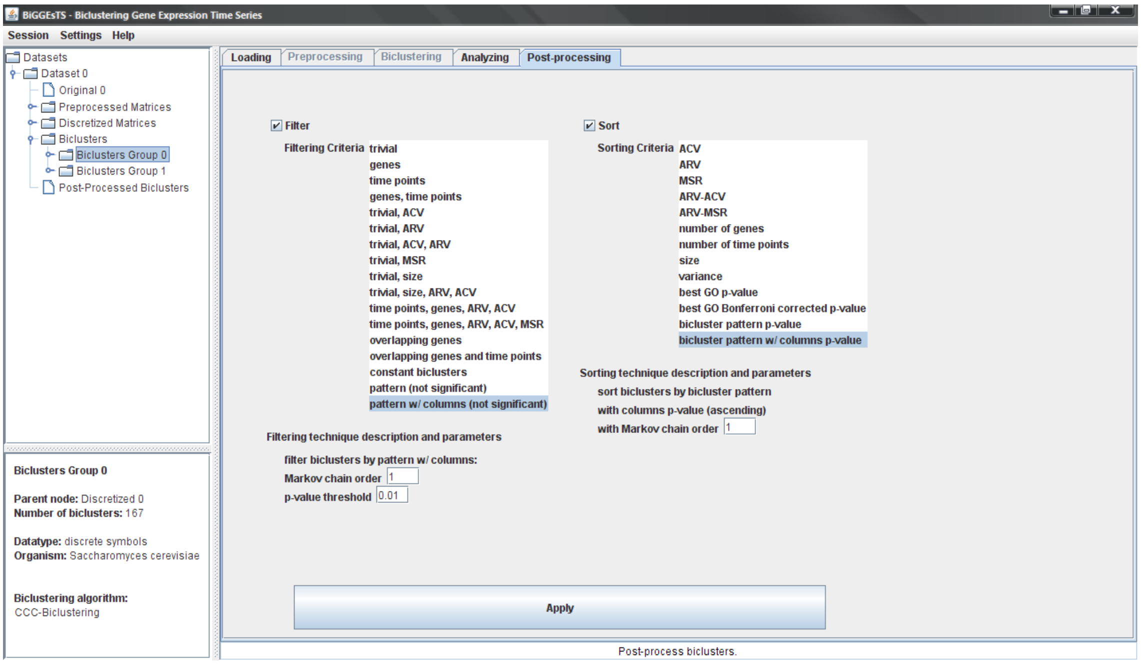

Post-Processing panel: available filtering and sorting options.

Below is a list of the available post-processing criteria for filtering and/or sorting the biclusters in a group of biclusters (if you need a formal definition of the metrics used in

post-processing techniques please see the section with formal definitions, in the end of this document):

Filtering
Filtering processes the group of biclusters and removes biclusters which: (i) are **trivial**, that is, are composed by a single row or a single column; contain (ii) a **number of genes** less than a given threshold, (iii) a **number of conditions** less than a given threshold, (iv) **a number of genes and a number of conditions** less than two corresponding thresholds; have (v) an **average column variance (ACV)** greater than a given threshold, (vi) an **average row variance (ARV)** less than a given threshold, (vii) an ACV greater than and an ARV less than two given thresholds, (viii) a **MSR** greater than a given threshold.

BiGGEsTS can also eliminate biclusters: whose (ix) **size**, the number of genes times the number of conditions, is less than a given threshold; which satisfy a combination of the previously mentioned criteria, such as the thresholds for their (x) **size, ARV and ACV**, (xi) **numbers of conditions and genes, ARV and ACV**, (xii) **numbers of conditions and genes, ARV, ACV and MSR**; which are very similar to other biclusters in the group, according to a given **percentage of similarity** on the dimension(s) of (xiii) the **genes** or (xiv) both **genes and conditions**; which (xv) are **constant**, that is, have no variation of the level of expression from one condition to another.

Two additional options for removing biclusters with **non significant expression patterns** are available for biclusters with discrete data, both using Markov chains to assess the statistical significance of the pattern and computing a p-value based on the (xvi) **overall** or (xvii) **column-wise** background probability of the occurrence of the pattern.

Sorting
Sorting the biclusters in a group reorganizes the biclusters according to: their values of (i) **ACV**, (ii) **ARV**, (iii) **MSR**; the **difference between their** (iv) **ARV and ACV**, (v) **ARV and MSR**. You can also sort biclusters by their **number of** (vi) **genes** or (vii) **conditions**, (viii) **size** or (ix) **variance**. These criteria allow biclusters to be sorted either in decreasing or decreasing order of the considered value(s).

Additional **sorting by the best p-value** obtained for the GO terms that annotate the genes of each bicluster in the group, both (x) **standard** and (xi) **corrected** for multiple testing, is also available. Groups of biclusters with discrete data can further be sorted based on the **significance of their expression pattern**, measured by a p-value computed using Markov models and based on the (xii) **overall** or (xiii) **column-wise** background probability of the occurrence of the pattern.

After post-processing a group of biclusters, a new group of biclusters is generated, which we call a **Post-Processed Biclusters Group**, and added to the dataset tree. This group is similar to a group of biclusters and allows for the very same functionalities. The resulting biclusters in a post-processed group can also be found in the original group. Because biclusters are just filtered and/or sorted, their data does not change in

relation to the original data. However, as a consequence of the filtering operation, the post-processed group may not contain all the original biclusters. Moreover, as a consequence of the sorting operation, the post-processed biclusters may also not be in the same order as in the original group. To address this, the post-processed biclusters are given new identifiers, following their order in the post-processed group, but they also maintain the original identifiers in parenthesis, (), for easier tracking the bicluster in the original group.

3. Formal Definitions

3.1. Expression matrix Let A be a $|R|$ row by $|C|$ column gene expression matrix defined by its set of rows (genes), R, and its set of columns (conditions), C. Let A_{ij} represent the expression level of gene i under condition j. Let A_{iC} and A_{Rj} denote row i and column j of matrix A, respectively.

3.2. Bicluster A bicluster is a submatrix A_{IJ} defined by $I \subseteq R$, a subset of rows, and $J \subseteq C$, a subset of columns. A bicluster with only one row or one column is called trivial.

3.3. COHERENT BICLUSTERS WITH CONTIGUOUS COLUMNS

CC-TSB algorithm Given an expression matrix A with absolute expression values, the number of biclusters to extract, an empirical threshold α for the ratio between the MSR of each row and the MSR of all the rows in the bicluster, an upper limit δ for the MSR of a bicluster and a maximum number of iterations, the goal is to identify and report a predefined number of biclusters with a good MSR value. Due to its heuristic nature, this approach is not guaranteed to find the optimal set of biclusters.

3.4. CONTIGUOUS COLUMN COHERENT BICLUSTERS WITH EXACT PATTERNS

CCC-Bicluster A contiguous column coherent bicluster (CCC-Bicluster) A_{IJ} is a subset of rows $I = \{i_1, \ldots, i_k\}$ and a subset of contiguous columns $J = \{r, r+1 \ldots, s-1, s\}$, such that $A_{ij} = A_{lj}$ for all rows $i, l \in I$ and columns $j \in J$. Each CCC-Bicluster defines a string S that is common to every row in I for the columns in J: the bicluster pattern.

Maximal CCC-Bicluster A CCC-Bicluster A_{IJ} is maximal if no other CCC-Bicluster exists that properly contains it, that is, for every other CCC-Bicluster A_{LM}, $I \subseteq L \wedge J \subseteq M \Rightarrow I = L \wedge J = M$. In other words, A_{IJ} is maximal if it cannot be extended with more genes or more contiguous columns.

CCC-Biclustering algorithm Given a discretized expression matrix A, the goal is to identify and report all maximal CCC-Biclusters.

3.5. CONTIGUOUS COLUMN COHERENT BICLUSTERS WITH APPROXIMATE PATTERNS

***e*-Neighborhood** The e-Neighborhood of a string S of length $|S|$, defined over the alphabet $\sum$ with $|\sum|$ symbols, $N(e, S)$ is the set of strings S_i such that $|S| = |S_i|$ and $Hamming(S, S_i) < e$, where e is an integer such that $e \geq 0$. This means that the Hamming distance between S and S_i is no more than e, that is, we need at most e substitutions to obtain S from S_i.

***e*-CCC-Bicluster** A contiguous column coherent bicluster with e errors per gene (*e*-CCC-Bicluster) is a CCC-Bicluster A_{IJ} where all the strings S_i that define the expression pattern of each gene in I are in the *e*-Neighborhood of an expression pattern S that defines the *e*-CCC-Bicluster: $S_i \in N(e,S), i \in I$. The definition of 0-CCC-Bicluster is equivalent to that of a CCC-Bicluster.

Maximal *e*-CCC-Bicluster An *e*-CCC-Bicluster A_{IJ} is maximal if it is row-maximal, left-maximal and right-maximal. This means that no more rows or contiguous columns can be added to I or J, respectively, maintaining the coherence property in the definition of *e*-CCC-Bicluster (above).

e-CCC-Biclustering algorithm Given a discretized expression matrix A and the integer $e \geq 0$, the goal is to identify and report all maximal *e*-CCC-Biclusters $B_k = A_{I_k J_k}$.

3.6. SIGN-CHANGES (ANTICORRELATED PATTERNS)

Opposite expression pattern Given the alphabet $\sum$ with $|\sum|$ sorted in lexicographic order and the expression pattern $S = [S[1] \dots S[|S|]]$, we define its opposite pattern S^{-1} as follows: $S^{-1} = [S[1]^{-1} \dots S[|S|]^{-1}]$, where $S[j]^{-1} \in \sum, j \in \{1, \dots, |S|\}$ is the opposite symbol of the symbol $S[j] \in \sum$. Assuming that $S[j]^{-1}$ corresponds to the symbol at position p in $\sum$, $\sum[p]$, we compute $\sum[p]^{-1}$ as follows: $\sum[p]^{-1} = \sum[|\sum| - p + 1], \forall p \in \{1, \dots, |\sum|\}$. When $|\sum|$ is odd, $\sum[|\sum|/2]^{-1} = \sum[|\sum|/2]$.

CCC-Bicluster with sign-changes A CCC-Bicluster with sign-changes A_{IJ} is a CCC-Bicluster such that $A_{ij} = A_{lj}$ or $A_{ij} = A_{lj}^{-1}$ for all rows $i, l \in I$ and columns $j \in J$, where A_{lj}^{-1} is the opposite symbol (in $\sum$) of that in A_{lj}.

***e*-CCC-Bicluster with sign-changes** An *e*-CCC-Bicluster with sign-changes A_{IJ} is an *e*-CCC-Bicluster where all the strings S_i that define the expression pattern of each of the genes in I are wither in the *e*-Neighborhood of the expression pattern S that defines the *e*-CCC-Bicluster, or in the *e*-Neighborhood of its opposite expression pattern S^{-1}: $S_i \in N(e,S)$ or $S_i \in N(e,S^{-1}), i \in I$.

3.7. GENE-SHIFTS (SCALED PATTERNS)

CCC-Bicluster with gene-shifts A CCC-Bicluster with gene-shifts A_{IJ} is a CCC-Bicluster where all the strings S_i that define the expression pattern S that defines the CCC-Bicluster, or one of the patterns resulting from shifting the expression pattern S K symbols up or K symbols down, where K is an integer and $K \in [1, \dots, |\sum| - 1]$. This means $S = S_i \vee S_i \in S^{\uparrow} = \{S^{\uparrow 1}, \dots, S^{\uparrow K}\} \vee S_i \in S^{\downarrow} = \{S^{\downarrow 1}, \dots, S^{\downarrow K}\}, i \in I$.

***e*-CCC-Bicluster with gene-shifts** An *e*-CCC-Bicluster with gene-shifts A_{IJ} is an *e*-CCC-Bicluster where all the strings S_i that define the expression pattern of each of the genes in I are either in the *e*-Neighborhood of the expression pattern S that defines

the *e*-CCC-Bicluster, or in the *e*-Neighborhood of the patterns resulting from shifting its expression pattern S K symbols up $S^{\uparrow} = \{S^{\uparrow 1}, \dots, S^{\uparrow K}\}$ or K symbols down $S^{\downarrow} = \{S^{\downarrow 1}, \dots, S^{\downarrow K}\}$, where K is an integer and $K \in [1, \dots, |\Sigma| - 1]$. This means $S_i = N(e, S) \vee S_i = N(e, S^{\uparrow}) \vee S_i = N(e, S^{\downarrow}), i \in I$.

3.8. TIME-LAGS (TIME LAGGED PATTERNS)

CCC-Bicluster with time-lags A CCC-Bicluster with time-lags A_{IJ} is a CCC-Bicluster such that $A_{ij} = A_{lj_l}$ for all rows $i, l \in I$ and columns $j_l \in J_l$. J_l is the set of contiguous columns corresponding to one occurrence of pattern S in row l, S_l, such that $j_l = j + lag_l$ and $lag_l \in \{0, \dots, |C| - 1\}$ is the time lag between S_l and the starting pattern.

Starting pattern (in CCC-Biclusters with time-lags) Given a pattern S and a set of rows I where it occurs at different time-lags, each of these occurrences is specified by a set of contiguous columns J_i. We consider the first (left most) occurrence of pattern S within all these occurrences specified by the contiguous columns in J_i, for all rows $i \in I$ as the starting pattern. This starting pattern is specified by a set of contiguous columns, $j \in J$, which corresponds to the set of contiguous columns where S occurs with time lag zero.

***e*-CCC-Bicluster with time-lags** An *e*-CCC-Bicluster with time-lags A_{IJ} is an *e*-CCC-Bicluster defined by the expression pattern S such that $A_{ij} = A_{lj_l}$, where $A_{lj_l} = S$ or $A_{lj_l} \in N(e, S)$ for all rows $i, l \in I$ and columns $j_l \in J_l$. J_l is the set of contiguous columns corresponding to one occurrence of pattern S, or one pattern in $N(e, S)$, in row l, S_l, such that $j_l = j + lag_l$ and $lag_l \in \{0, \dots, |C| - 1\}$ is the time lag between S_l and the starting pattern.

Starting pattern (in *e*-CCC-Biclusters with time-lags) Given a pattern S in $N(e, S)$ and a set of rows I where these patterns occur at different time-lags, each of these occurrences is specified by a set of contiguous columns J_i. We consider the first (left most) occurrence of pattern S, or a pattern in $N(e, S)$, within all these occurrences specified by the contiguous columns in J_i, for all rows $i \in I$ as the starting pattern. This starting pattern is specified by a set of contiguous columns, $j \in J$, which corresponds to the set of contiguous columns where S, or a pattern in $N(e, S)$, occurs with time lag zero.

3.9. POST-PROCESSING METRICS

Size

$|R| \times |C|$

Variance

$$\frac{\sum_{i=1}^{|R|}\sum_{j=1}^{|C|}\left(A_{ij}-m_A\right)^2}{|R|\times|C|}, \qquad m_A=\frac{\sum_{i=1}^{|R|}\sum_{j=1}^{|C|}A_{ij}}{|R|\times|C|}$$

Average Column Variance (ACV)

$$\frac{\sum_{i=1}^{|R|}\sum_{j=1}^{|C|}\left(A_{ij}-\left(\frac{\sum_{k=1}^{|R|}A_{kj}}{|R|}\right)\right)^2}{|R|\times|C|}$$

Average Row Variance (ARV)

$$\frac{\sum_{i=1}^{|R|}\sum_{j=1}^{|C|}\left(A_{ij}-\left(\frac{\sum_{k=1}^{|C|}A_{ik}}{|C|}\right)\right)^2}{|R|\times|C|}$$

Mean Squared Residue (MSR)

$$\frac{\sum_{i=1}^{|R|}\sum_{j=1}^{|C|}\left(A_{ij}-\left(\frac{\sum_{k=1}^{|R|}A_{kj}}{|R|}\right)-\left(\frac{\sum_{l=1}^{|C|}A_{il}}{|C|}\right)+m_A\right)^2}{|R|\times|C|}$$

Bicluster similarity

In order to compute the similarity measure between two biclusters, $B_1=(I_1,J_1)$ and $B_2=(I_2,J_2)$ we use the Jaccard Index. This score is used to measure the degree of overlap between two biclusters both in terms and conditions and is defined as follows: $S(B_1,B_2)=S\big((I_1,J_1),(I_2,J_2)\big)=\frac{|B_1\cap B_2|}{|B_1\cup B_2|}=\frac{|B_{11}|}{|B_{01}|+|B_{10}|-|B_{11}|}$, where $B_{11}=\{(i,j):(i,j)\in B_1\wedge(i,j)\in B_2\}$, $B_{10}=\{(i,j):(i,j)\in B_1\wedge(i,j)\notin B_2\}$ and $B_{01}=\{(i,j):(i,j)\notin B_1\wedge(i,j)\in B_2\}$, for the genes $i\in I_1\cup I_2$ and the conditions $j\in J\cup J_2$. Similarly, the gene and condition similarities can be computed, respectively, as follows: $S(I_1,I_2)=\frac{|I_1\cap I_2|}{|I_1\cup I_2|}$ and $S(J_1,J_2)=\frac{|J_1\cap J_2|}{|J_1\cup J_2|}$.

Significance of a bicluster pattern

This is a superficial description of the computation of the significance of a bicluster pattern. For more details see [1].

The statistical significance of a CCC-Bicluster B is p-value(B), which is computed by obtaining the probability $P(p_B)$ of a random occurrence of the pattern of the bicluster, p_B, under the null hypothesis k times in n independent trials, where k is the number of genes in the bicluster minus 1 and n is the number of genes in the expression matrix minus 1. The null hypothesis assumes that the expression levels of the genes evolve independently.

The value of this probability is obtained by computing the *tail of the binomial distribution*, which gives the probability of an event with probability p_B occurring k or more times in n independent trials.

We use the simplifying assumption that the probability of occurrence of a specific expression pattern p_B, $P(p_B)$, is adequately modeled by a first order Markov Chain, with state transition probabilities obtained from the values in the corresponding columns in the matrix.

For a CCC-Bicluster with sign-changes, P is defined as: $P(p_B \cup p_B^{-1}) = P(p_B) + P(p_B^{-1})$, where p_B^{-1} is the opposite (symmetric) pattern of p_B.

For a CCC-Bicluster with time-lags, P is defined as: $P((p_B)_{\rightarrow}^{LAG}) = \sum_{lag|B} P\left((p_B)_{\rightarrow}^{lag}\right)$, where $lag \in \{0, \dots, |C|-1\}$ and the values of lag are restricted to those occurring in B, $lag|B$.

For a CCC-Bicluster with time-lags, P is defined as: $P((p_B)_{\rightarrow}^{LAG} \cup (p_B^{-1})_{\rightarrow}^{LAG}) = \sum_{lag|B} P\left((p_B)_{\rightarrow}^{lag}\right) + \sum_{lag|B} P\left((p_B^{-1})_{\rightarrow}^{lag}\right)$, where $lag \in \{0, \dots, |C|-1\}$ and the values of lag are restricted to those occurring in B, $lag|B$.

For a CCC-Bicluster with gene-shifts (scaled patterns), P is defined as: $P\left(p_B \cup p_B^{\uparrow} \cup p_B^{\downarrow}\right) = P(p_B) + \sum_{shift} P\left(p_B^{\uparrow shift}\right) + \sum_{shift} P\left(p_B^{\downarrow shift}\right)$, where $shift \in \{1, \dots, K\}$ and K is the value used in the CCC-Biclustering algorithm with gene-shifts to shift the expression pattern K symbols up and down.

In the case of an e-CCC-Bicluster, the statistical significance or the value of p-value(B), is computed by obtaining the probability of a random occurrence under the null hypothesis of the expression patterns in the e-Neighborhood of the expression pattern p_B defining the e-CCC-Bicluster, $N(e, p_B)$, k times in n independent trials, where k is the number of genes in the bicluster minus 1 and n is the number of genes in the expression matrix minus 1.

This is performed using the simplifying assumption that the probability of occurrence of a specific expression pattern in the e-Neighborhood of the pattern p_B, $N(e, p_B)$, is adequately modeled by a first order Markov Chain, with state transition probabilities obtained from the values in the corresponding columns in the matrix. In the general case, $P(N(e, p_B)) = \sum_{i=1}^{|N(e,p_B)|} P(N(e, p_B)[i])$, where $N(e, p_B)$ and $N(e, p_B)[i]$ are, respectively, the number of patterns and the ith pattern in the e-Neighborhood of the pattern p_B.

When missing values are considered as valid errors, $N(e, p_B)$ is computed using the alphabet $\sum' \cup mv'$, where mv is the symbol used for missing value and each element mv' is obtained by concatenating m and one number in the range $\{1, \ldots, |C|\}$, that is, $mv' = \{mv\} \times \{1, \ldots, |C|\}$.

When only restricted errors are allowed, $N(e, p_B)$ is not computed using all the symbols in $\sum'$. The allowed substitutions for each symbol in p_B are the z neighbors, both to the left and to the right of $\sum'[p]$ that are considered as valid errors, where p is the position of the symbol $p_B[k]$ in $\sum'$.

For an e-CCC-Bicluster with sign-changes, P is computed as $P\left(N(e, p_B) \cup N(e, p_B^{-1})\right) = P\left(N(e, p_B)\right) + P\left(N(e, p_B^{-1})\right)$, where $P\left(N(e, p_B)\right) = \sum_{i=1}^{|N(e,p_B)|} P(N(e, p_B)[i])$, $P\left(N(e, p_B^{-1})\right) = \sum_{i=1}^{|N(e,p_B^{-1})|} P(N(e, p_B^{-1})[i])$. $|N(e, p_B)|$, $|N(e, p_B^{-1})|$, $N(e, p_B)[i]$ and $N(e, p_B^{-1})[i]$ are, respectively, the number of patterns and the ith pattern in the e-Neighborhood of the pattern p_B and p_B^{-1}, respectively.

For an e-CCC-Bicluster with time-lags, P is computed as $P\left(N(e, (p_B)_{\rightarrow}^{LAG})\right) = \sum_{lag|B} P\left(N\left(e, (p_B)_{\rightarrow}^{lag}\right)\right)$.

For an e-CCC-Bicluster with sign-changes and time-lags, P is computed as $P\left(N(e, (p_B)_{\rightarrow}^{LAG}) \cup N(e, (p_B^{-1})_{\rightarrow}^{LAG})\right) = \sum_{lag|B} P\left(N\left(e, (p_B)_{\rightarrow}^{lag}\right)\right) + P\left(N\left(e, (p_B^{-1})_{\rightarrow}^{lag}\right)\right)$.

For an e-CCC-BIcluster with gene-shifts (scaled patterns), P is computed as $P\left(N(e,p_B) \cup N\left(e,p_B^{\uparrow}\right) \cup N\left(e,p_B^{\downarrow}\right)\right) =$

$P\left(N(e,p_B)\right) + \sum_{shift} P\left(N\left(e,p_B^{\uparrow shift}\right)\right) + \sum_{shift} P\left(N\left(e,p_B^{\downarrow shift}\right)\right).$

References

[1] Sara C. Madeira, **Efficient Biclustering Algorithms for Time Series Gene Expression Data Analysis**, PhD Thesis, Instituto Superior Técnico, Technical University of Lisbon, Dec 2008.
