# Supplementary material for: BiGGEsTS: integrated environment for biclustering analysis of time series gene expression data
Source: BMC Res Notes. 2009 Jul 7;2:124. doi: 10.1186/1756-0500-2-124 (PMC2720980; doi:10.1186/1756-0500-2-124)
Supplement: Additional file 6 — Case study: discovering transcriptional modules using BiGGEsTS. Case study describing how to use BiGGEsTS to discover transcriptional regulatory modules using the transcriptional response of Saccharomyces cerevisiae to heat stress. The results published in [6] are reproduced. [file 1756-0500-2-124-S6.pdf]

Discovering transcriptional regulatory modules using BiGGEsTS
A case study

Joana P. Gonçalves*[1,2,3], Sara C. Madeira[1,2,3] , Arlindo L. Oliveira[1,2]

[1]Knowledge Discovery and Bioinformatics (KDBIO) group, INESC-ID, Rua Alves Redol, Apartado 13069, 1000-029 Lisboa, Portugal
[2]Instituto Superior Técnico, Technical University of Lisbon, Av. Rovisco Pais, 1049-001 Lisboa, Portugal
[3]University of Beira Interior, Rua Marquês d'Ávila e Bolama, 6201-001 Covilhã, Portugal

Email: Joana P. Gonçalves*- jpg@kdbio.inesc-id.pt; Sara C. Madeira - smadeira@kdbio.inesc-id.pt; Arlindo L. Oliveira - aml@inesc-id.pt;

*Corresponding author

Abstract

This document provides supplementary material to the manuscript *"BiGGEsTS: integrated environment for biclustering analysis of time series gene expression data"*. We present a case study, describing how to use the software to discover transcriptional regulatory modules in a dataset containing the response of *Saccharomyces cerevisiae* to heat stress, and reproducing the results published in [1].

Dataset

Throughout the case study we analyze the **Yeast Stress** dataset, used by Madeira et al. [1], and directly downloadable from the CCC-Biclustering website [2]. This dataset, derived from the experiment identified as *"heat shock 2"* in the original group of datasets from Gasch et al. [3], concerns the response of 6142 *Saccharomyces cerevisiae* genes to heat shock, and comprises five different time points along the first hour of exposure to 37ºC (0, 5, 15, 30 and 60 minutes).

Loading the dataset and performing a preliminary analysis

Load the file **yeast_stress.txt**, containing the **Yeast Stress** dataset, using the "Loading" tab and the default options. The corresponding table of colors in now displayed in the "Analyzing" tab (Figure 1). The dataset is identified by the node "Dataset 0" in the tree shown in top left panel. The bottom left panel contains information about the dataset: a cDNA expression matrix from *Saccharomyces cerevisiae* with 6142 genes and 5 time points, and contains missing values (colored yellow in the table of colors).

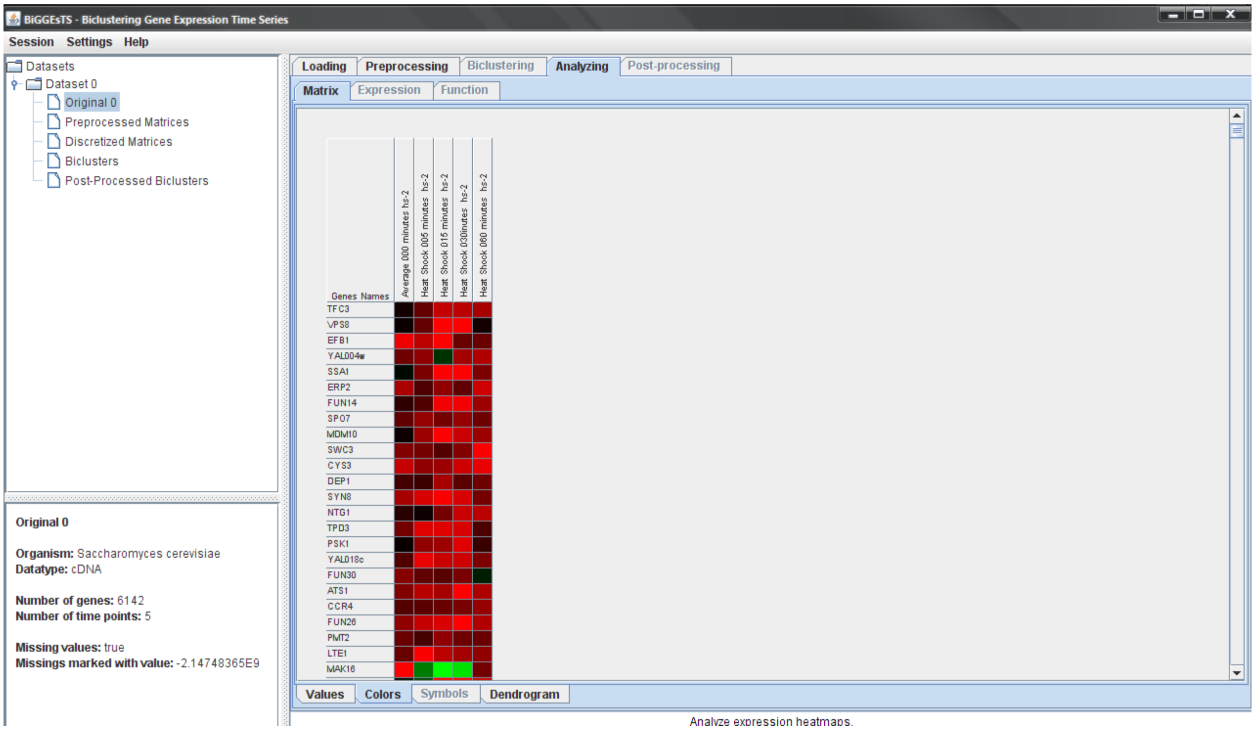

Figure 1: Table of colors.

Perform a preliminary analysis of the dataset, not only by inspecting the table of colors, but also by analyzing the table of values (Figure 2), the dendrogram (Figure 3), and the table with the list of GO terms annotating the genes in the dataset, together with the set of genes annotated with a specific term of interest (Figure 4).

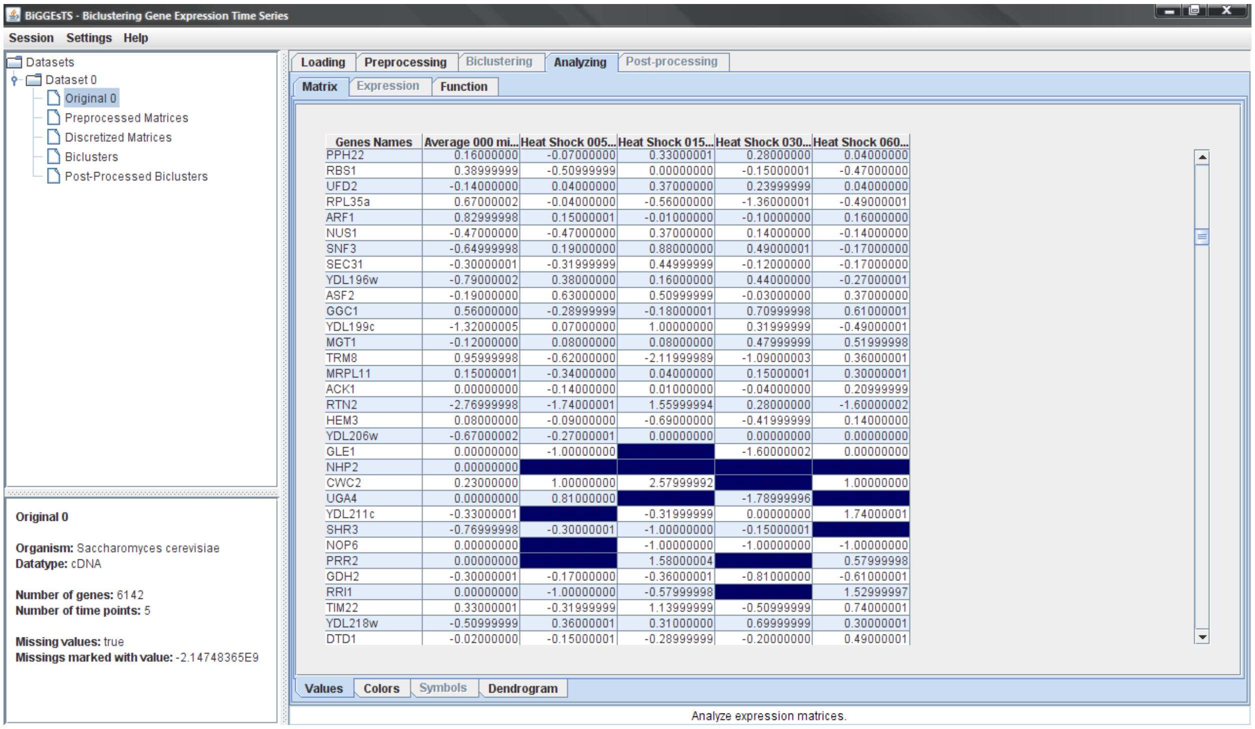

Figure 2: Table of values.

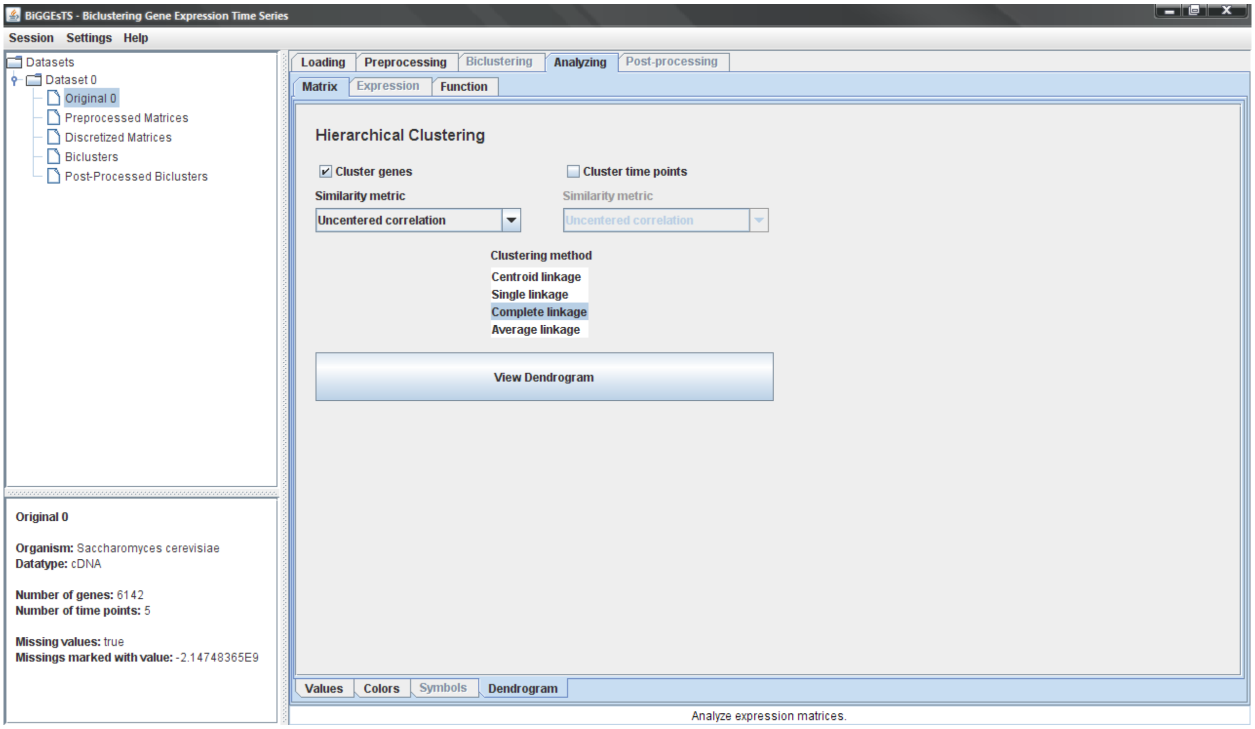

(a)

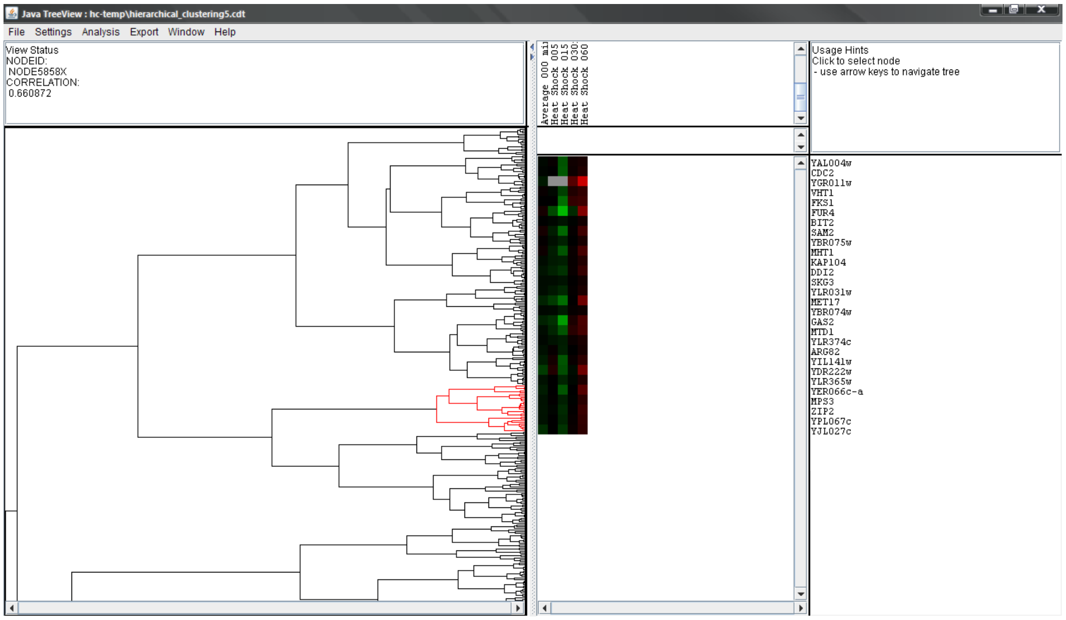

(b)

Figure 3: Dendrogram.

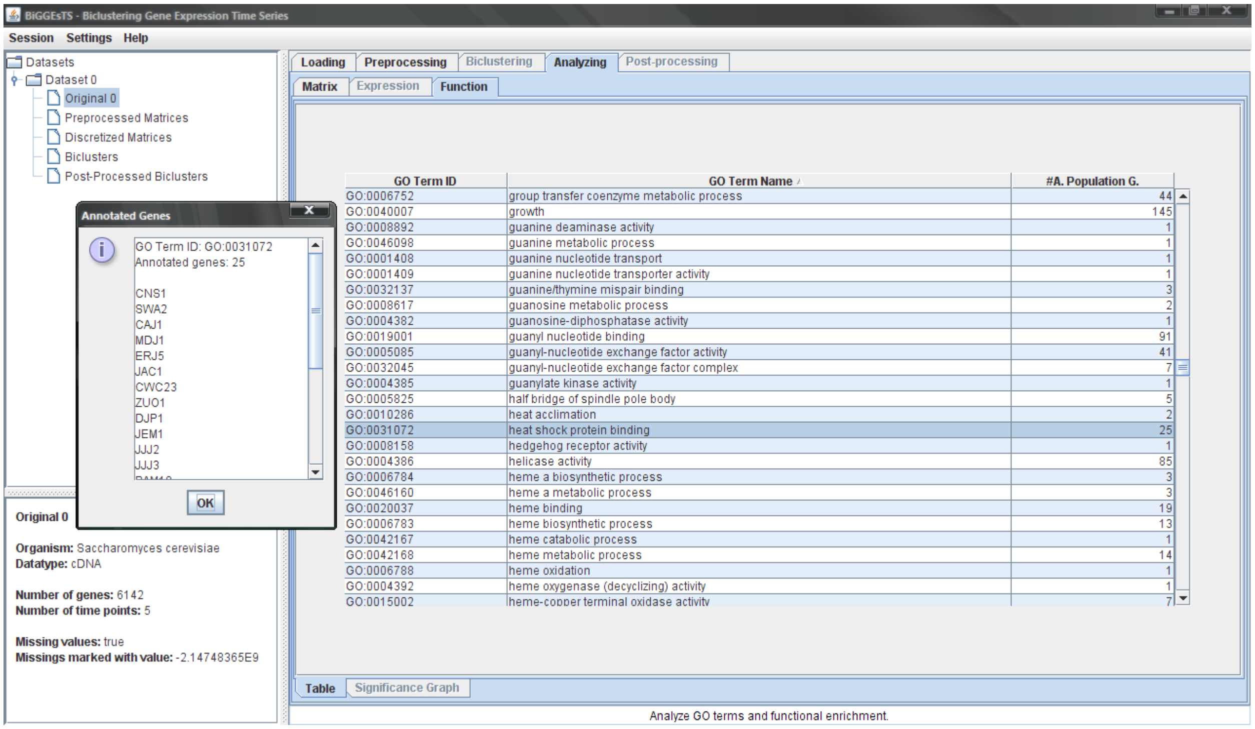

Figure 4: Table with GO terms.

Preprocessing the dataset

Preprocess "Dataset 0" as shown in Figure 5(a). As in [1], the genes with missing values are removed, and the gene profiles are normalized to zero mean and standard deviation. Since the biclustering algorithm used in this case study, CCC-Biclustering, processes discretized matrices, the dataset is discretized using a three symbol alphabet {D, N, U}, where "D", "N" and "U" stand for down-regulation, no-change and up-regulation, respectively. The technique identified as "variation between time points (3 symbols)", originally proposed by Ji and Tan [4], is used with the corresponding threshold set to 1.

Figure 5(b) shows the table of symbols, representing the discretized version of the expression matrix in "Dataset 0", "Preprocessed Matrices", identified as "Preprocessed 0". In this table, the colors green, black, and red, identify the symbols "D", "N" and "U", respectively. Note that a node representing this discretized matrix, named "Discretized 0", was created in the tree on the top left panel.

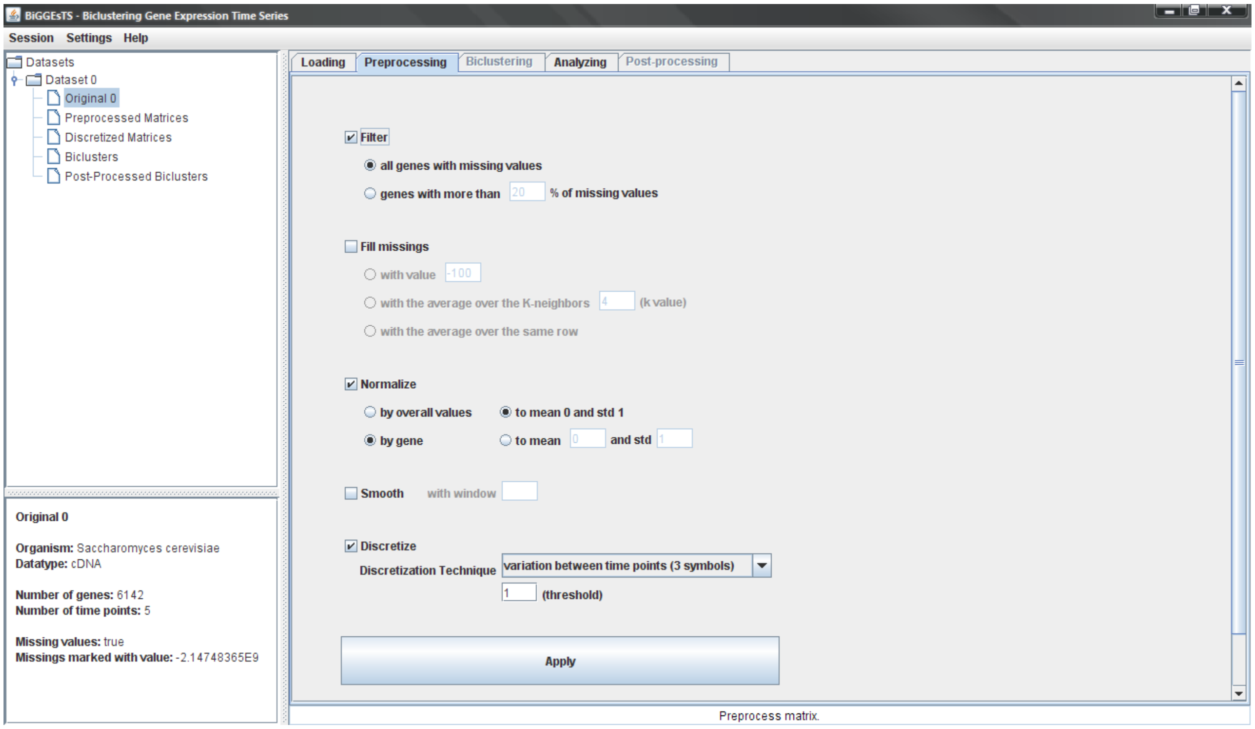

(a)

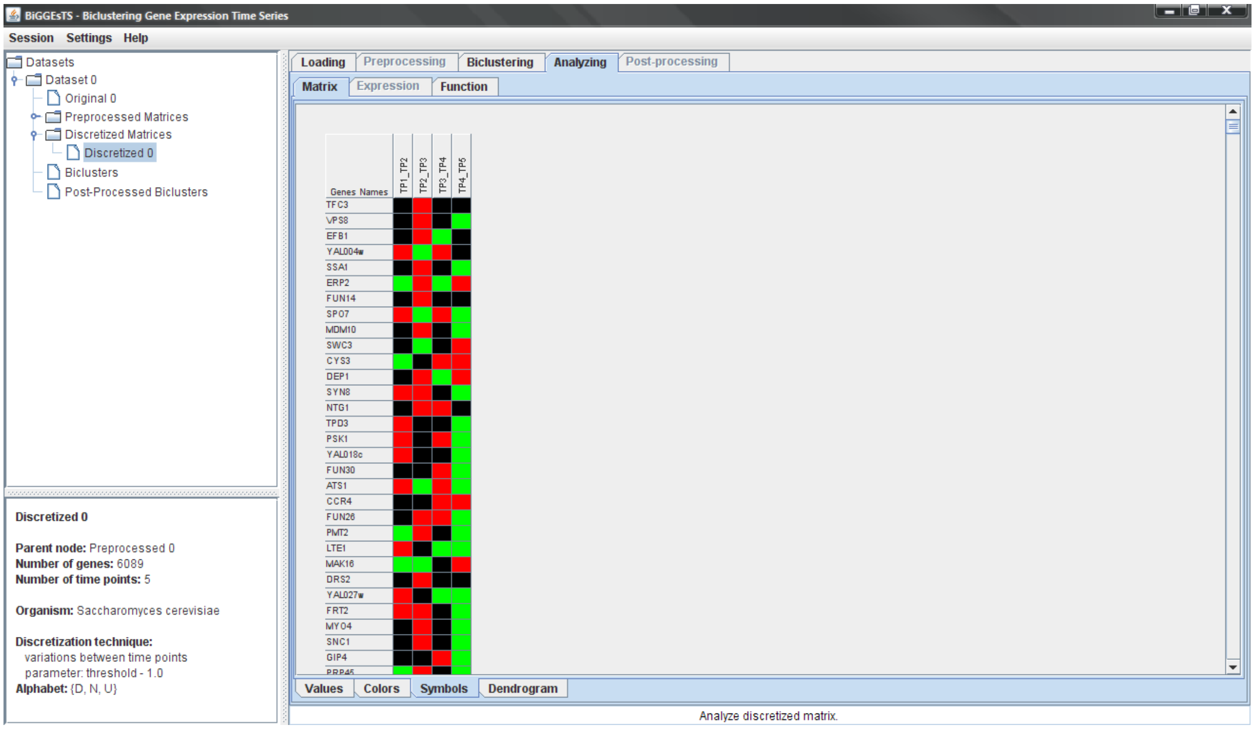

(b)

Figure 5: Preprocessing.

Biclustering yeast stress time series

Apply CCC-Biclustering (without extensions) to the discretized matrix “Discretized 0”, as shown in Figure 6(a). This algorithm identifies 167 biclusters (Figure 6(b)). These biclusters can be visualized by expanding the node identified as “Biclusters Group 0” in the tree on the top left panel, which represents the group of biclusters discovered by CCC-Biclustering when applied to “Discretized 0”. Note that the biclusters are listed according to the order they are extracted by the algorithm. No quality criterion has been applied yet. Still, we can analyze their matrices of values, colors and symbols, and inspect their expression profiles using expression and pattern charts. These charts can be displayed for the group of biclusters or individual biclusters. We can also analyze GO annotations and functional enrichment results. However, we postpone these analyses until we perform the post-processing steps described in [1].

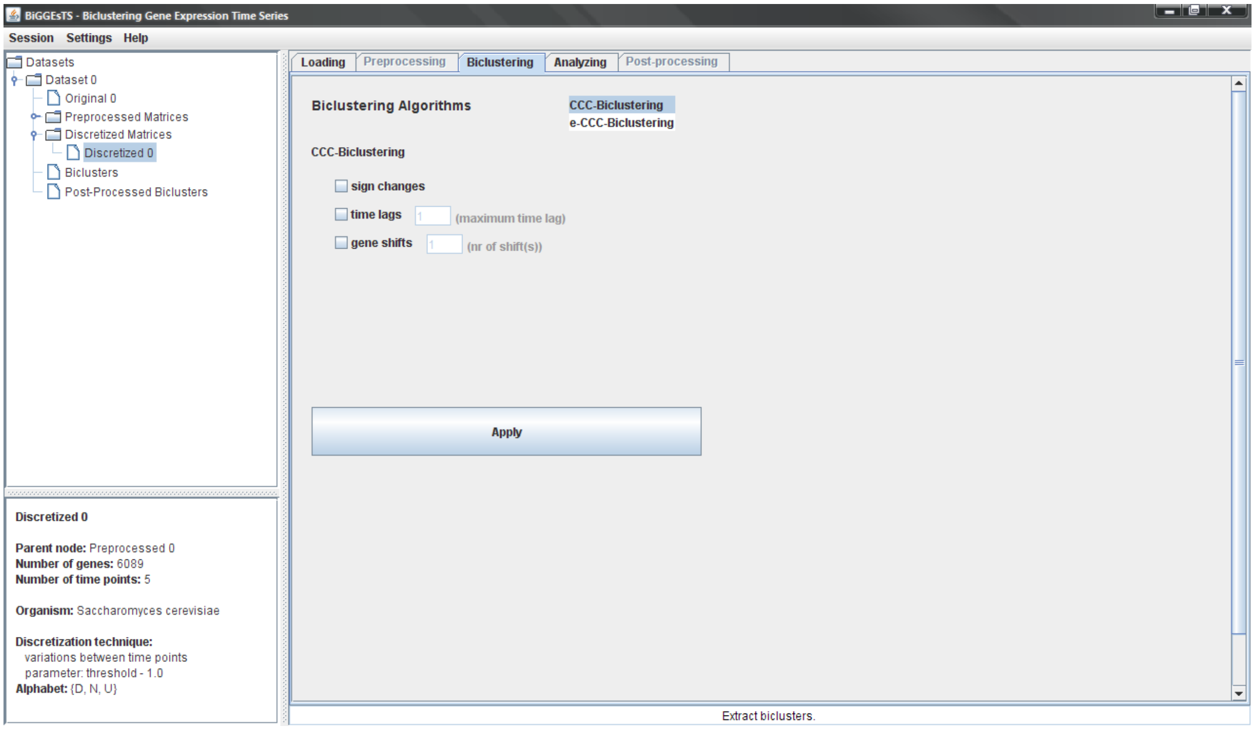

(a)

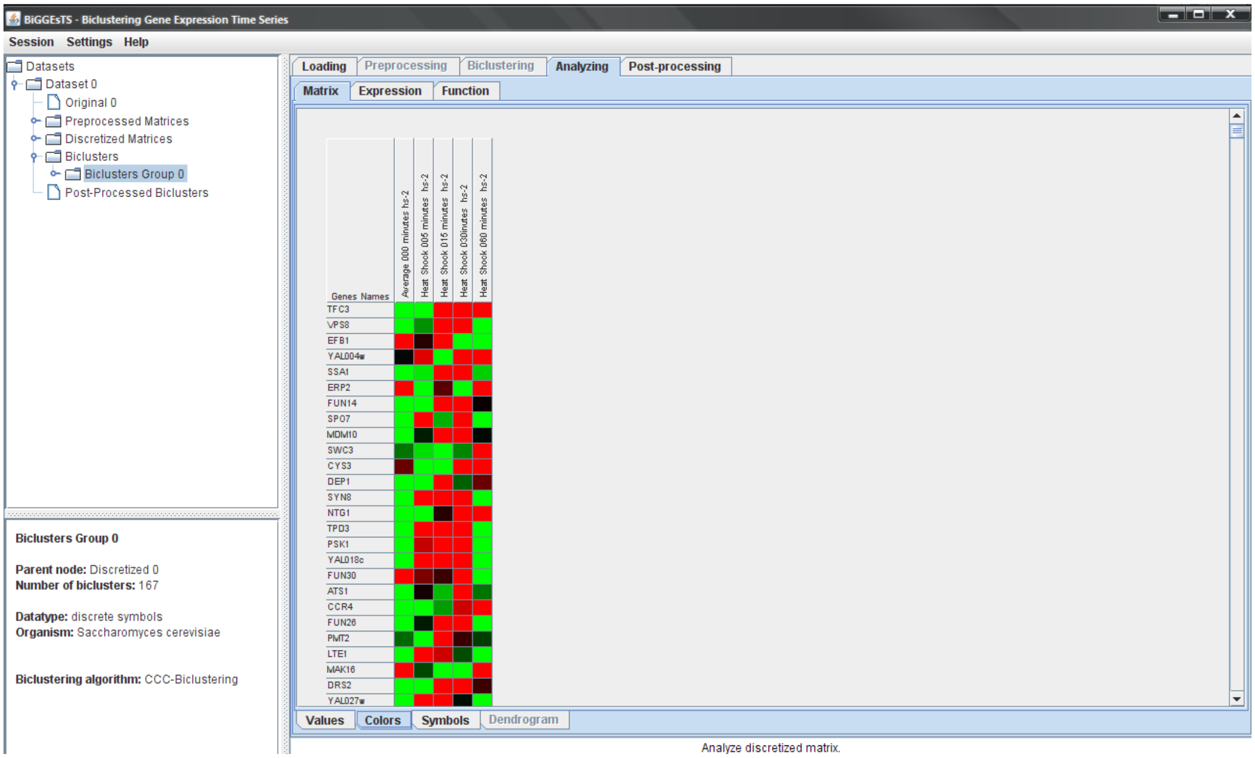

(b)

Figure 6: Biclustering.

Post-processing biclustering results

Post-processing techniques are performed in order to rank biclusters according to their relevance. In [1], the authors were interested in biclusters with high statistical significance. As such, in a first post-processing step, the set of 167 biclusters discovered using CCC-Biclustering were filtered using their p-values, computed as described in [1]. All biclusters which were not highly significant at the 1% level after applying the Bonferroni correction for multiple testing were discarded. The remaining biclusters were then sorted in ascending order of their p-values.

Figure 7 shows how to apply these filtering and sorting steps in BiGGEsTS. The filtering technique "pattern w/columns (not significant)", applied with the parameters Markov chain order and p-value threshold set to 1 and 0.01, respectively, correspond to computing the p-value of each bicluster using a first order Markov chain, and discarding those whose p-value does not pass the statistical significance test at the 1% level, after Bonferroni correction for multiple testing. The sorting technique "bicluster pattern w/ columns p-value" ranks the biclusters surviving the filtering step using their p-values. The resulting group of biclusters is identified as "Post-Processed Group 0" in the tree on the top left panel.

In order to avoid the analysis of highly overlapping biclusters, the similarities between the sorted biclusters were computed using the Jaccard similarity score, and biclusters with similarity greater than 25% were filtered, in a second post-processing step. This can easily be performed in BiGGEsTS by post-processing the "Post-Processed Group 0" using the filtering technique "overlapping genes and time points" and setting the similarity threshold to 25 (Figure 8). The resulting group of biclusters, identified as "Post-Processed Group 1" in the tree on the top left panel, corresponds to the set of biclusters analyzed in [1], and will now be subject to further analysis.

Figure 9 shows the expression profiles of these biclusters using expression and pattern charts. Two types of chronological expression patterns can be identified: transcriptional up-regulation patterns (biclusters 14, 27, 39, 48, 43, 83, 42, 79 and 92) and transcriptional down-regulation patterns (biclusters 107, 163, 151, 142, 147, 148, 159 and 99). A subset of these biclusters were analyzed in detail in [1], using GO annotations together with information about transcriptional regulations.

In this case study, the biological analysis is focused on bicluster 14, the top bicluster in the ranking, which has 1091 genes and 4 time points. Figure 10 shows its expression profile, describing a transcriptional up-regulation pattern with a short delay, between 5 and 15 minutes of exposure to heat shock, classified as "middle up regulation" in [1].

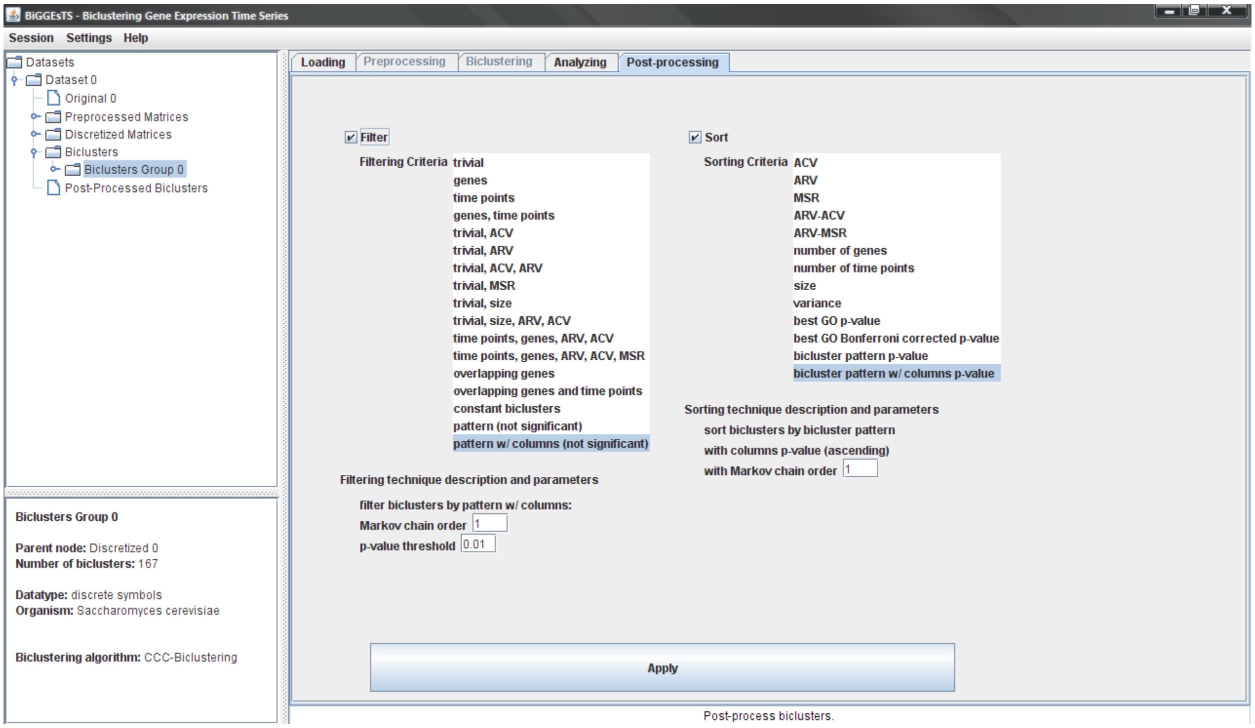

(a)

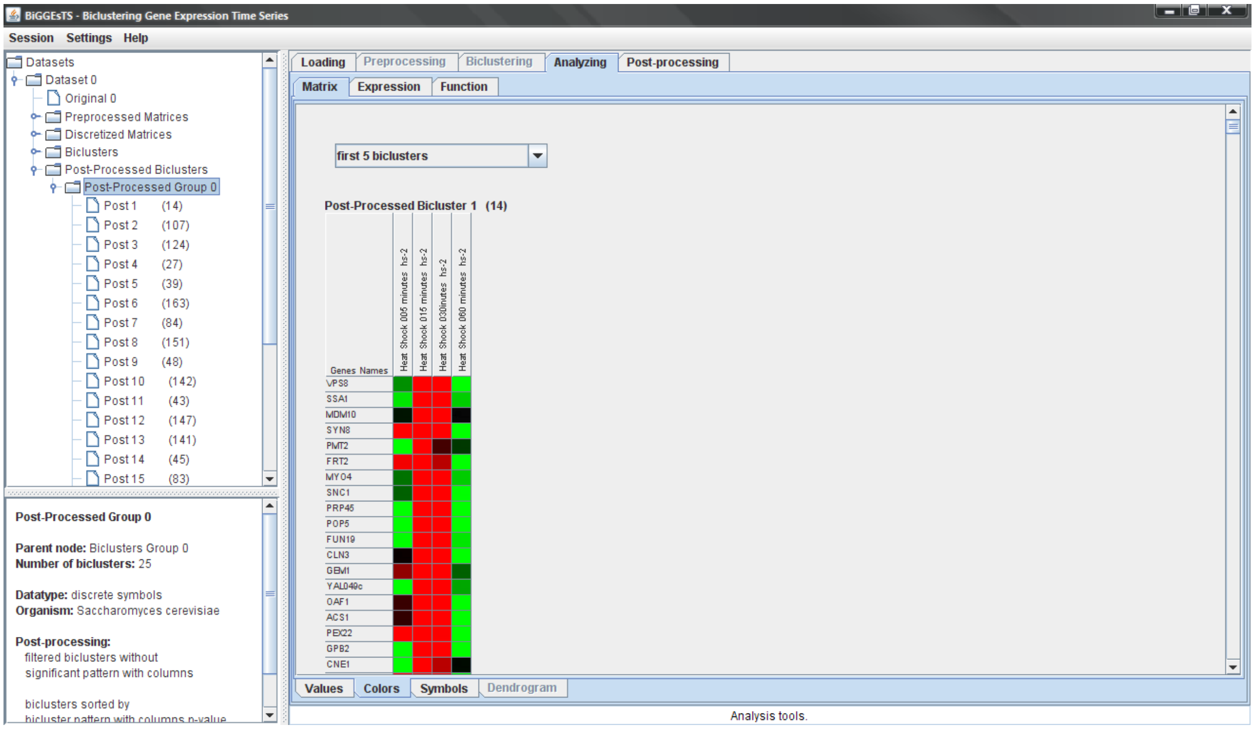

(b)

Figure 7: Post-processing step 1: filtering biclusters with Bonferroni corrected p-values greater than 0.01 and sorting the remaining ones in ascending order of these p-values.

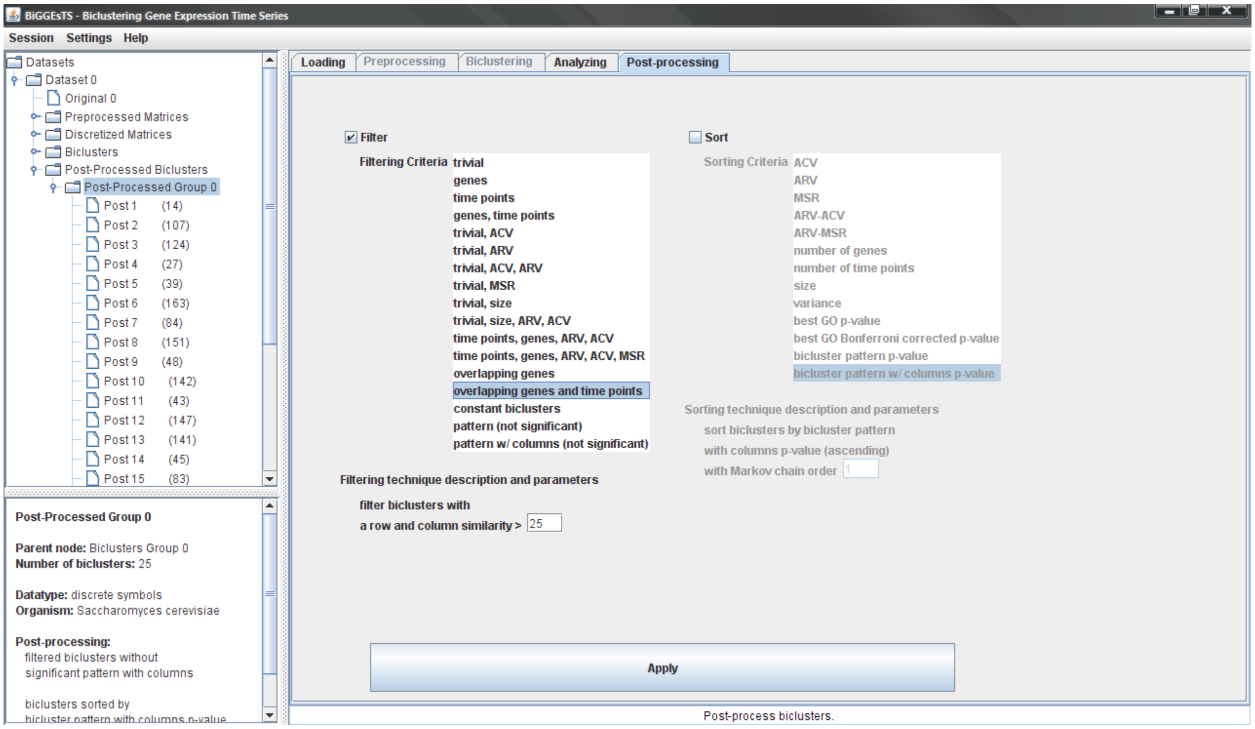

(a)

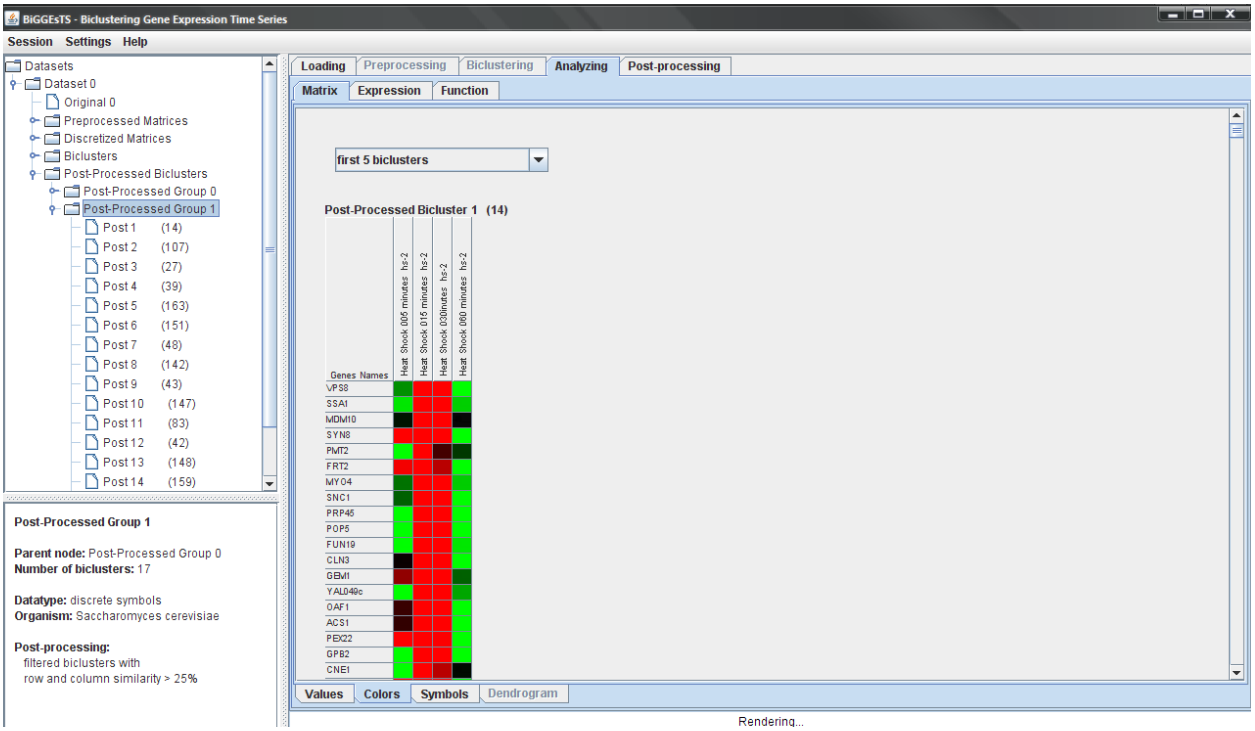

(b)

Figure 8: Post-processing step 2: filtering biclusters with similarities above 25%. This filtering processing is applied to the biclusters resulting from step 1. These are thus sorted in ascending order of p-value before the overlapping filter is applied and keep their ranking after having been filtered.

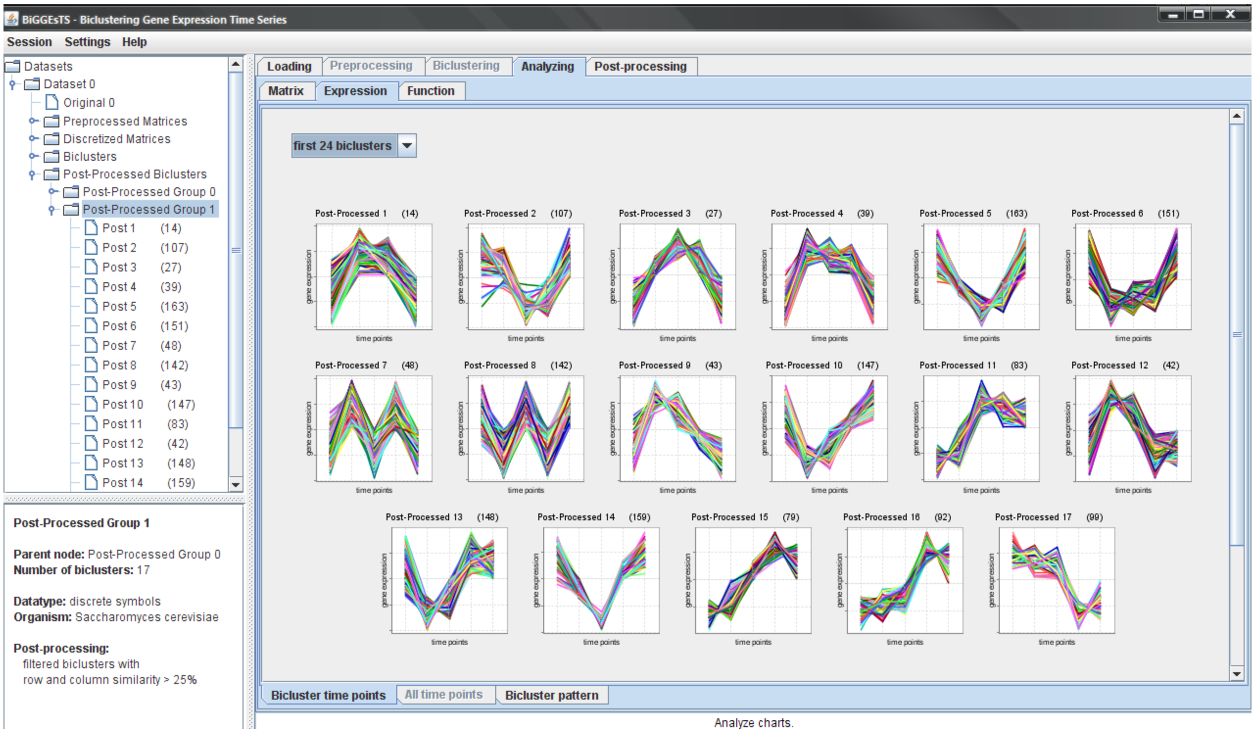

(a)

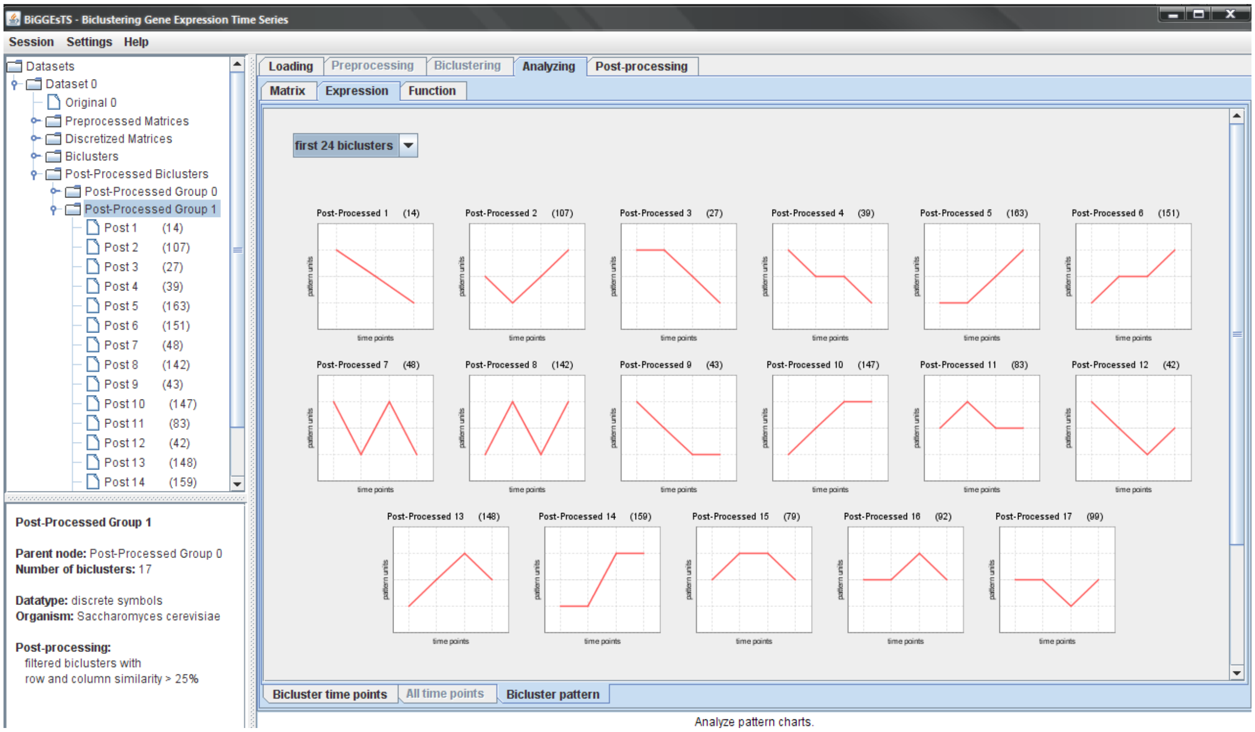

(b)

Figure 9: Biclusters: Expression and pattern charts.

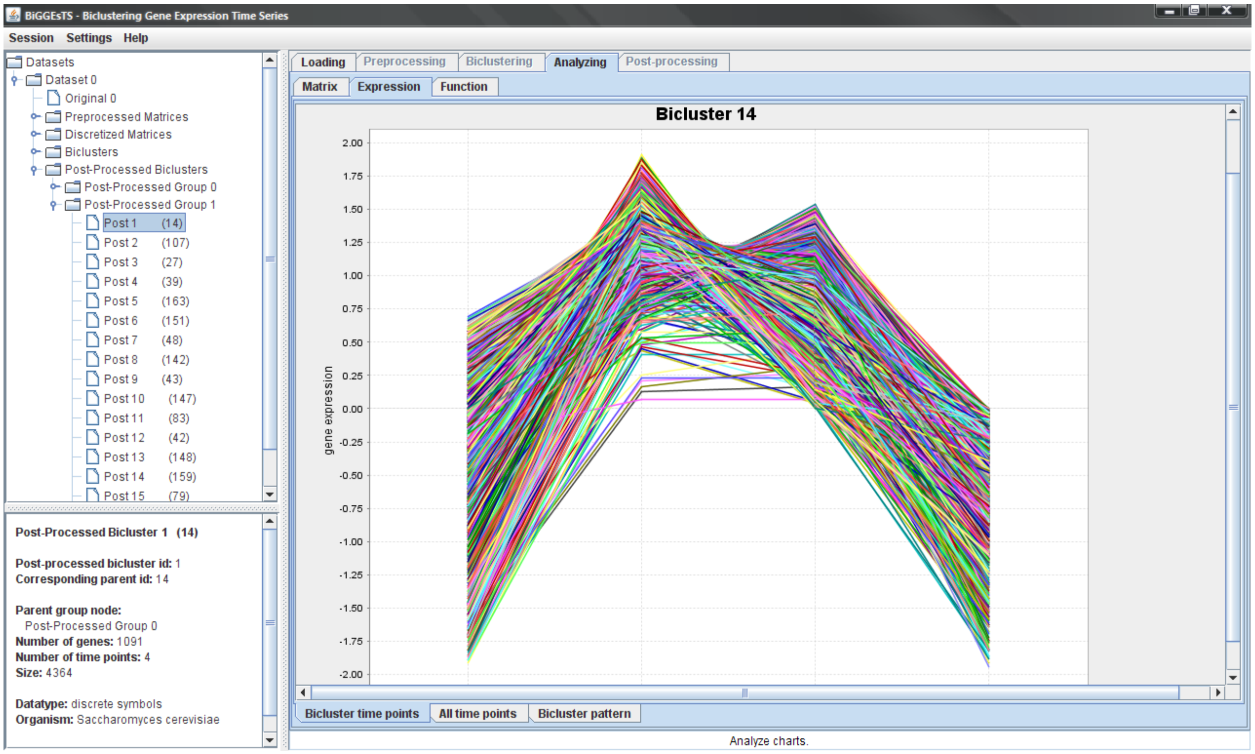

Figure 10: Bicluster 14: Expression chart.

Biological analysis

Figure 11 shows the result of the functional enrichment analysis performed for the biclusters in "Post-Processed Group 1". Note that even when there are no significant terms associated with a given bicluster, whose expression pattern was considered as statistically significant (Figure 11), the bicluster should be further analyzed since it might as well identify biologically relevant phenomena. This was in fact shown in [1], when performing the biological analysis of this group of biclusters.

Figure 12 shows the detailed functional enrichment analysis performed for bicluster 14, together with the set of genes annotated with the GO term "response to stress". Figure 13 displays the graph of enriched terms, highlighting the terms in the ontology "Biological Process", which are the relevant ones when the aim of the biological analysis is the discovery of transcriptional regulatory modules. A similar GO-based analysis can be performed for the other biclusters in "Post-Processed Group 1".

Focusing on bicluster 14, and on GO terms in the "Biological Process" ontology, the GO-based analysis reveals the occurrence of highly significant terms, including "carbohydrate metabolic process" or "energy derivation by oxidation of organic compounds", related to energy generation, and "response to stimulus" or "response to stress", related to the cellular response to heat shock. These terms are consistent with the induction of protein folding chaperones aiming at protecting against, and recovering from, protein unfolding with associated energetic expenses. The transcriptional induction of genes involved in alternative carbon source metabolism and respiration, in the presence of glucose, is considered a consequence of a sudden decrease in cellular ATP concentration, caused by ATP-consuming stress defense mechanisms [1,3].

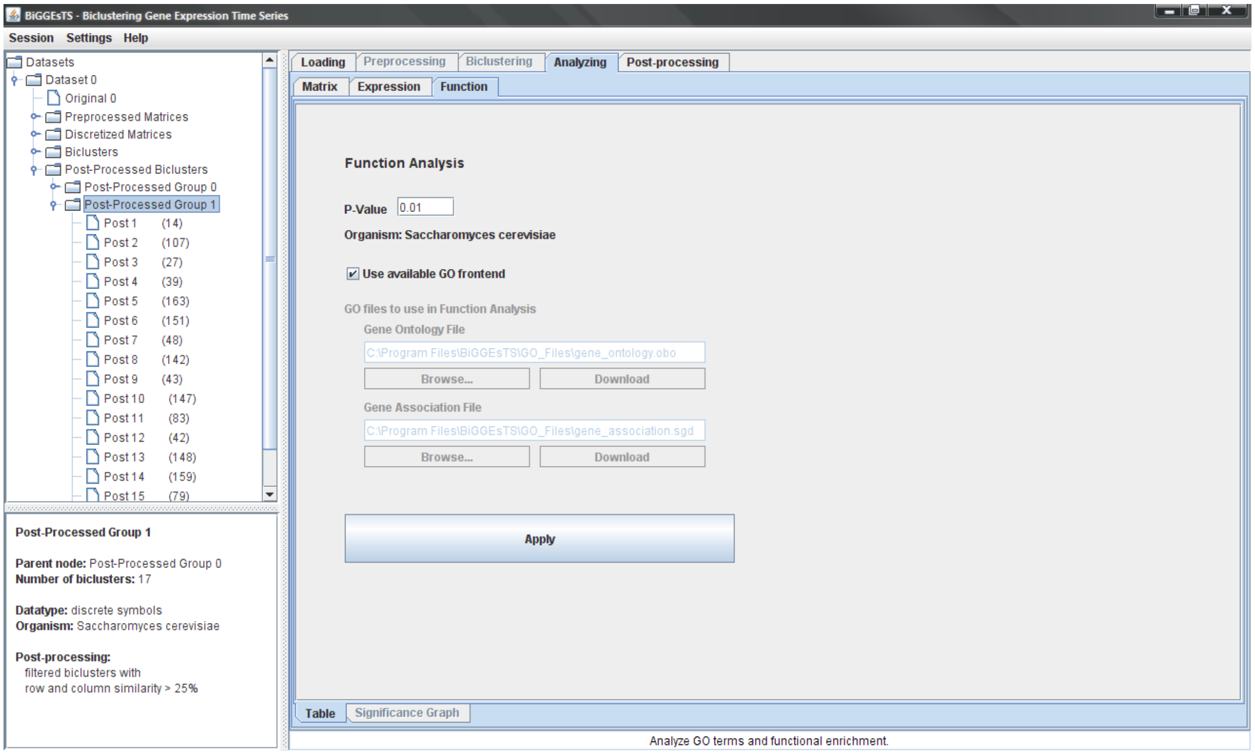

(a)

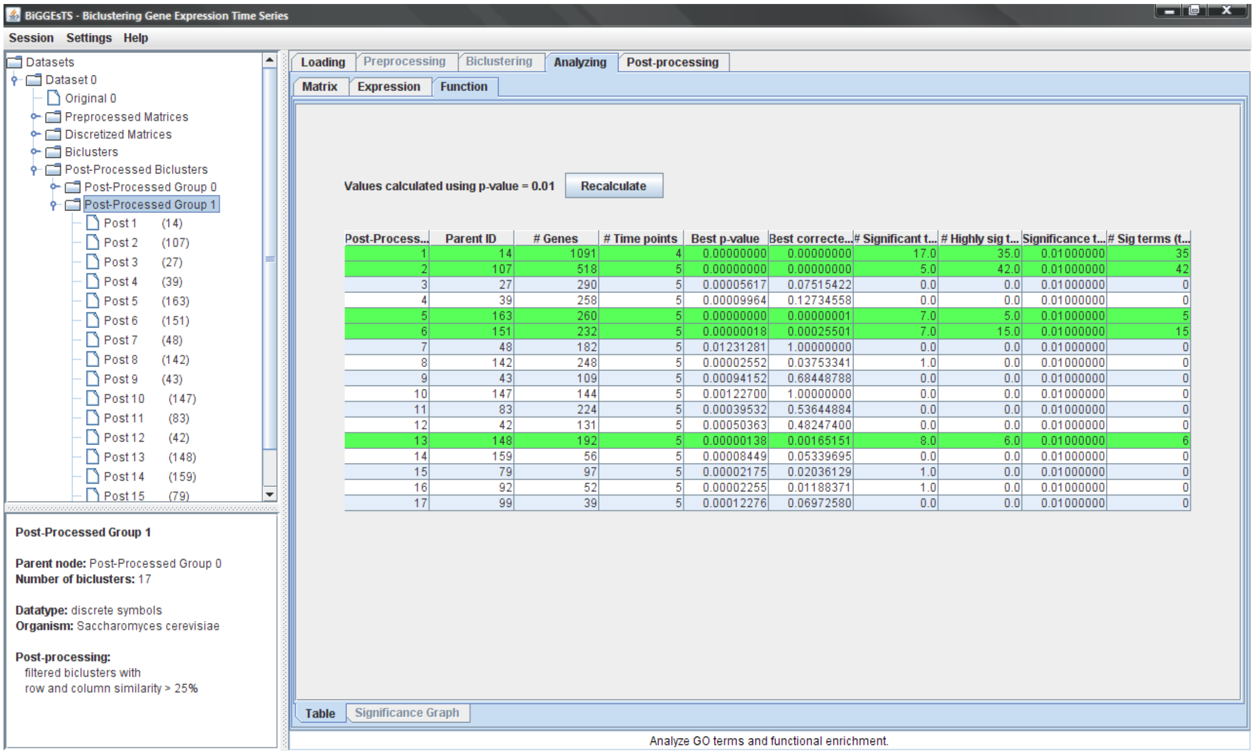

(b)

Figure 11: Biclusters: Functional enrichment.

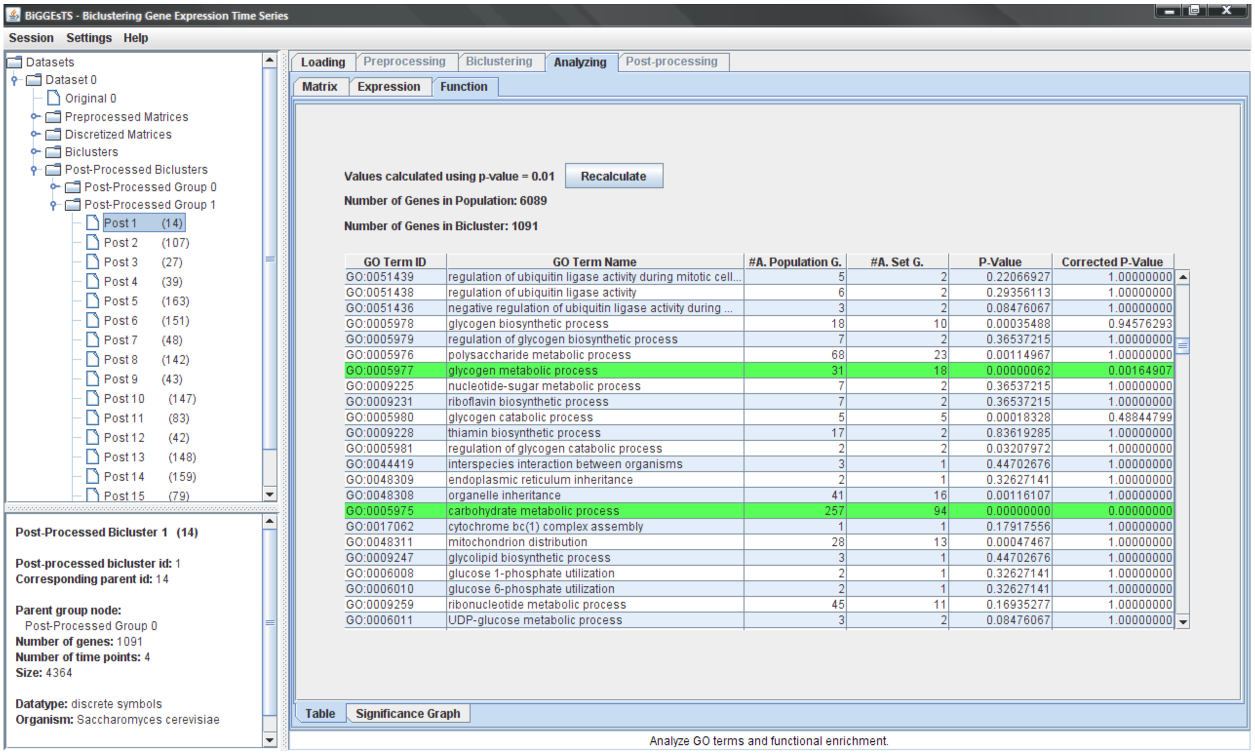

(a)

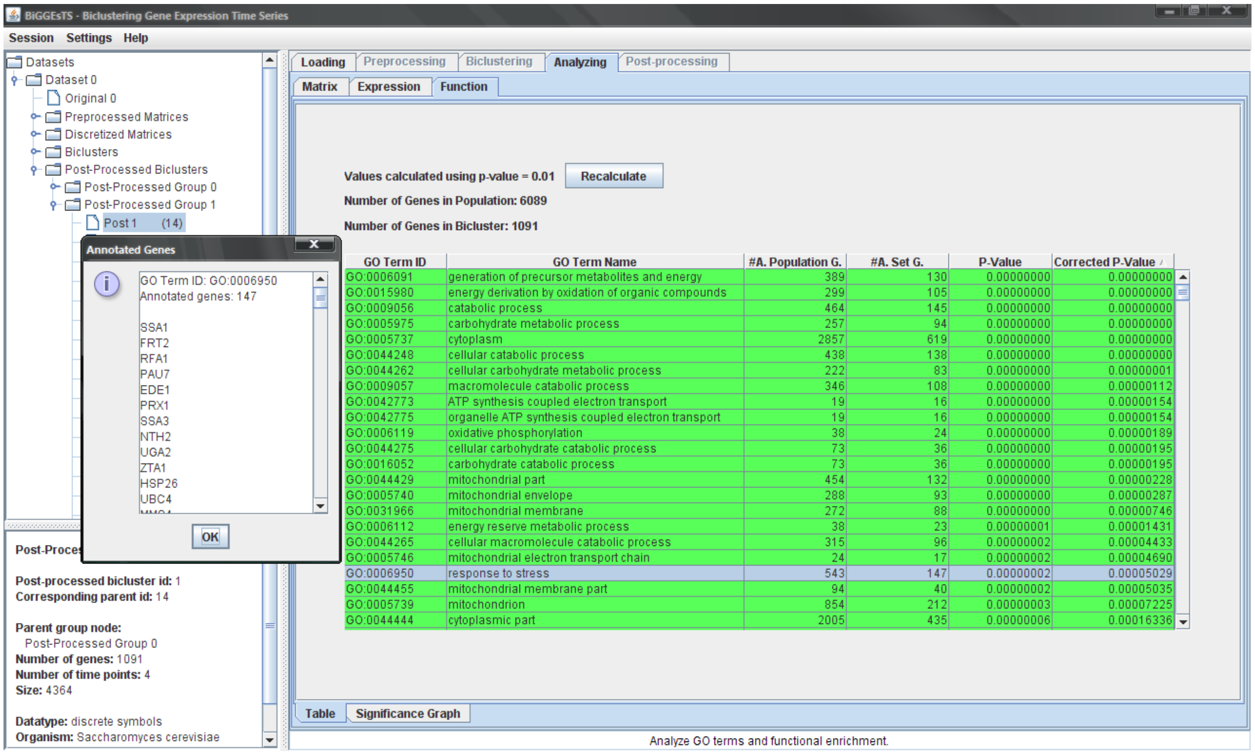

(b)

Figure 12: Bicluster 14: Functional enrichment.

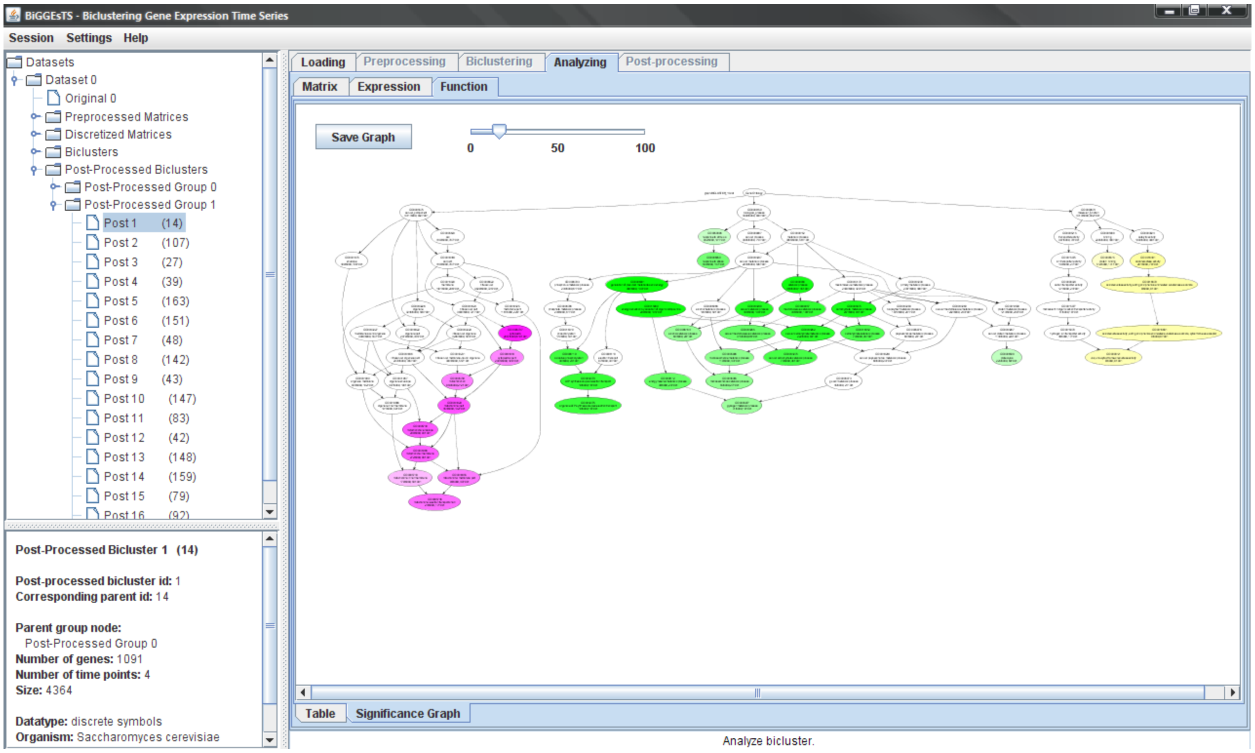

(a)

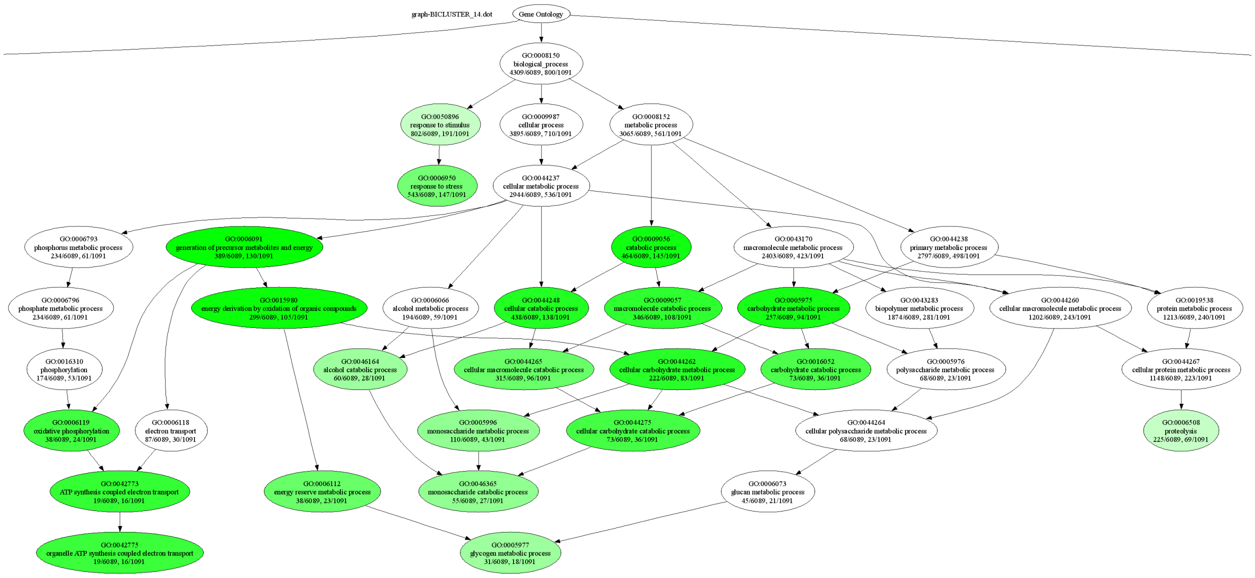

(b)

Figure 13: Bicluster 14: Graph of enriched terms.

Authors' contributions

SCM wrote the case study. All authors read and approved the final version.

References

1. Madeira SC, Teixeira MC, Sá-Correia I, Oliveira AL: **Identification of regulatory modules in time series gene expression data using a linear time biclustering algorithm**. *IEEE/ACM Transactions on Computational Biology and Bioinformatics* 2008, [http://doi.ieeecomputersociety.org/10.1109/TCBB.2008.34].

2. **CCC-Biclustering**. *http://kdbio.inesc-id.pt/software/ccc-biclustering/, [October 6, 2008].*

3. Gasch A, Spellman P, Kao C, Carmel-Harel O, Eisen M, Storz G, Botstein D, Brown P: **Genomic expression program in the response of yeast cells to environmental changes**. *Molecular Biology of the Cell* 2000, **11**(12):4241–4257.

4. Ji L, Tan K: **Identifying time-lagged gene clusters using gene expression data**. *Bioinformatics* 2005, **21**(4):509–516.
